# Higher handgrip strength is linked to higher salience ventral attention functional network segregation in older adults

**DOI:** 10.1038/s42003-024-05862-x

**Published:** 2024-02-21

**Authors:** Joanna Su Xian Chong, Kevin Yiqiang Chua, Kwun Kei Ng, Shin Wee Chong, Ruth L. F. Leong, Michael W. L. Chee, Woon Puay Koh, Juan Helen Zhou

**Affiliations:** 1 Centre for Sleep and Cognition & Centre for Translational Magnetic Resonance Research, Yong Loo Lin School of Medicine, National University of Singapore, Singapore, Singapore; 2 Integrative Sciences and Engineering Programme (ISEP), NUS Graduate School, National University of Singapore, Singapore, Singapore; 3 Healthy Longevity Translational Research Programme, Yong Loo Lin School of Medicine, National University of Singapore, Singapore, Singapore; 4 Singapore Institute for Clinical Sciences, Agency for Science Technology and Research (A*STAR), Singapore, Singapore; 5 Department of Electrical and Computer Engineering, National University of Singapore, Singapore, Singapore

**Keywords:** Cognitive ageing, Ageing

## Abstract

Converging evidence suggests that handgrip strength is linked to cognition in older adults, and this may be subserved by shared age-related changes in brain function and structure. However, the interplay among handgrip strength, brain functional connectivity, and cognitive function remains poorly elucidated. Hence, our study sought to examine these relationships in 148 community-dwelling older adults. Specifically, we examined functional segregation, a measure of functional brain organization sensitive to ageing and cognitive decline, and its associations with handgrip strength and cognitive function. We showed that higher handgrip strength was related to better processing speed, attention, and global cognition. Further, higher handgrip strength was associated with higher segregation of the salience/ventral attention network, driven particularly by higher salience/ventral attention intra-network functional connectivity of the right anterior insula to the left posterior insula/frontal operculum and right midcingulate/medial parietal cortex. Importantly, these handgrip strength-related inter-individual differences in salience/ventral attention network functional connectivity were linked to cognitive function, as revealed by functional decoding and brain-cognition association analyses. Our findings thus highlight the importance of the salience/ventral attention network in handgrip strength and cognition, and suggest that inter-individual differences in salience/ventral attention network segregation and intra-network connectivity could underpin the handgrip strength-cognition relationship in older adults.

## Abstract (web summary)

An fMRI study reveals that weaker handgrip strength is related to lower salience/ventral attention network segregation, driven particularly by anterior insula connectivity, which could lead to poorer cognition in community-dwelling older adults.

## Introduction

Handgrip strength, an indicator of skeletal muscle strength, is an important determinant of physical frailty and healthy ageing^1–3^. While several measures of muscle strength and physical frailty exist, handgrip strength is of particular interest because of its low cost, ease of administration^4^, good reliability^5^ and strong predictive value for health and cognitive outcomes^6,7^. Lower handgrip strength has been widely associated with reduced quality of life^8,9^, as well as disability and mortality in older age^10–14^. Converging evidence also suggests that lower handgrip strength is linked to cognitive impairment and lower cognitive function in older adults^15–19^. A coordinated analysis of nine longitudinal ageing studies, for example, has demonstrated consistent association between greater decline in handgrip strength and greater decline in cognitive function^15^. Further, strength and resistance training has been shown to improve cognition in older adults^20–22^, underscoring the potential of such physical interventions for preventing and slowing cognitive impairment. Given the close link between handgrip strength and cognition, it has been suggested that convergent age-related changes in brain function and structure may underlie this relationship between handgrip strength and cognitive function in older adults^23^. Understanding the neural mechanisms underlying the handgrip strength-cognition relationship is therefore of interest and could facilitate the development of new interventions aimed at mitigating age-related cognitive and physical decline.

Resting-state fMRI functional connectivity, which measures the statistical correlations between BOLD signal fluctuations of different regions under task-free settings, has been widely used to examine age-related changes in brain functional organization^24,25^ and could serve as a tool to understand the shared neural substrates underlying both handgrip strength and cognitive function. To date, only a handful of studies have examined associations between handgrip strength and functional connectivity, and little remains known about how such inter-individual differences in handgrip strength-related functional connectivity can be associated with cognition. One study reported a link between handgrip strength and somatomotor functional connectivity in healthy older adults, with higher handgrip strength and tapping frequency being associated with higher functional connectivity of the motor cortex to the putamen, cerebellum and insula^26^. Studies have linked other somatomotor measures (e.g., gait speed) and the physical frailty phenotype (e.g., Fried’s frailty phenotype) to inter-individual differences in functional connectivity of somatomotor regions or networks in older adults^27–29^ as well, indicating that handgrip strength and other somatomotor measures might specifically implicate functional brain networks that are involved in motor function. However, there is evidence suggesting that motor performance also requires attention control, which is a higher-order cognitive function involved in response inhibition and selection^30^. In a study examining motor performance and attention control in hemiparetic stroke and healthy participants, higher performance on force tracking and maximum-force grip tasks were associated with higher integrity of both the somatomotor network and the attention control (salience) network^30^. Further, performance on these tasks were linked to attention control (measured as distractor resistance)^30^. Thus, convergent disruptions in functional connectivity of the salience network – a network anchored in the anterior insula and dorsal anterior cingulate cortex that is involved in salience processing and attentional and cognitive control^31^—could potentially underlie concurrent decline in handgrip strength and cognitive function in ageing.

One key limitation of these studies is the lack of a whole-brain perspective when examining handgrip strength or other motor function-related inter-individual differences in functional connectivity. Most studies focused on the functional connectivity of specific regions or networks (particularly the somatomotor networks), which are limited in providing insight on how handgrip strength and other motor abilities are related to cognitive function. To this end, studying the functional segregation of the brain could shed light on the handgrip strength-cognition relationship. Functional segregation quantifies the ability for the brain to form networks that have strong intra-network but weak inter-network connections^32^, and reflects the functional specialization of networks supporting different processes^33^. A loss of segregation could hence indicate increased blurring across networks and decreased communication within networks^33,34^, leading to performance declines across multiple processes. Convergent findings across studies utilizing different analytical approaches to quantify functional segregation have demonstrated age-related declines in system-wide segregation of functional brain networks, particularly that of the higher-order associative networks^34–41^. Importantly, lower functional segregation has been associated with poorer cognitive performance in healthy older adults^34,37,42^, suggesting that loss of segregation of functional networks could drive cognitive decline in ageing. Further, higher segregation of somatomotor networks have been demonstrated to relate to better gross (including handgrip strength) and fine somatomotor performance, particularly in older adults^43,44^. Nevertheless, such studies have focused only on the segregation of somatomotor networks, and little is known whether and how functional segregation loss of diverse networks across the whole brain relates to handgrip strength and cognition.

The aim of the study is therefore to examine the association between handgrip strength and functional segregation of the various networks across the brain in community-dwelling older adults, and how inter-individual differences in the functional segregation of handgrip strength-related networks may be linked to inter-individual differences in cognitive function. Specifically, we hypothesized that higher handgrip strength would be related to higher brain network segregation in the somatomotor and salience networks. Further, we expect that individuals with higher functional segregation in these handgrip strength-related networks would have better cognitive performance. Finally, we hypothesized that these associations would remain even after accounting for the time difference between MRI scan and handgrip assessment, or the influence of other variables that have been reported to relate to handgrip strength, such as depressive symptoms, indices of obesity or the extent of cognitive impairment in participants.

## Results

### Higher handgrip strength is associated with better cognitive performance

We first tested the associations between handgrip strength and cognitive performance in 148 community-dwelling older adults (see Table 1 for participants’ cognitive and demographic characteristics). Higher handgrip strength in older adults was associated with better global cognition, processing speed and attention performance (FDR-adjusted *p* < 0.05, corrected for multiple comparisons across the five cognitive measures) (Table 2), supporting the link between handgrip strength and cognition. Handgrip strength was however not significantly associated with executive function or SM-MMSE scores, although there was a trend towards a positive association for these cognitive measures. These results remained after controlling for Geriatric Depression Scale scores, body mass index or waist-hip ratio. Additionally, the findings for global cognition, processing speed and attention performance remained after controlling for SM-MMSE scores (Supplementary Tables 1, 2).

**Table 1:** Participant demographic and cognitive characteristics

Characteristic	Participants (*n* = 148)
Age in years, mean (SD)	72.8 (3.9, 62.2–82.4)
Sex, female/male	83/65
Years of education, mean (SD)	7.28 (3.97, 0–16)
SM-MMSE score, mean (SD)	26.4 (2.4, 17–30)
Waist-hip ratio, mean (SD)	0.917 (0.068, 0.743–1.080)
Body mass index, mean (SD) (*n* = 146)	23.0 (3.4, 16.4–36.9)
GDS score, mean (SD)	2.33 (2.46, 0–14)
Cognitive domain scores, mean (SD)	
Global	50.0 (6.1, 31.9–65.6)
Processing speed	50.3 (8.3, 27.8–81.0)
Attention	49.6 (6.8, 25.9–71.7)
Executive function	50.1 (6.5, 38.3–68.0)
Physical frailty measures	
Handgrip strength (kg), mean (SD, range)	22.3 (7.0, 8.3–37.7)
Weight change (%)^a^, mean (SD, range)	-0.29 (7.38, −30.77–25.69)
Timed Up-And-Go time (s), mean (SD, range)	10.3 (4.0, 6–43)
Exhaustion^b^, yes/no	25/123

*SD* standard deviation, *SM-MMSE* Singapore-modified version of the mini-mental state examination, *GDS* geriatric depression scale.

^a^Weight change (%) measures the percent weight change since previous follow-up (mean duration = 7.03 (SD = 0.82, range = 4.45–8.65) years).

^b^For the exhaustion measure, participants were asked to indicate yes or no to the question “Do you feel full of energy?”.

**Table 2:** Associations between handgrip strength and cognitive measures (*n* = 148)

Cognitive measure	Coefficient	SE	t	Uncorr p	FDR-adj p
Global	0.251	0.076	3.30	0.0012*	0.0059*
Processing speed	0.327	0.106	3.09	0.0024*	0.0059*
Attention	0.237	0.098	2.41	0.0172*	0.0287*
Executive function	0.187	0.091	2.06	0.0412	0.0516
SM-MMSE	0.043	0.035	1.23	0.2196	0.2196

All models controlled for age, sex and education. Multiple comparison correction was performed across the five cognitive measures.

*SE* standard error, *Uncorr* uncorrected, *FDR-adj* false discovery rate-adjusted, *SM-MMSE* Singapore-modified version of the mini-mental state examination.

*indicates statistically significant effects (*p* < 0.05).

### Higher handgrip strength is associated with higher system segregation and intra-network functional connectivity of the salience/ventral attention network

**Fig. 1:**
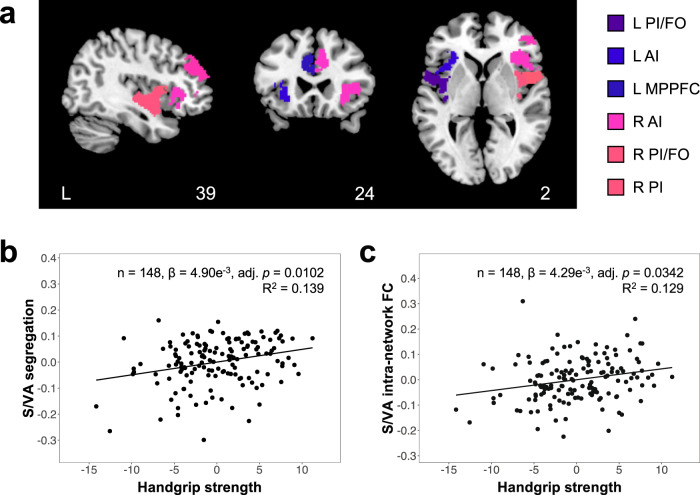
Higher handgrip strength is associated with higher salience/ventral attention network segregation, which is driven by higher salience/ventral attention intra-network connectivity. **a** Brain map depicts select brain regions of the salience/ventral attention network overlaid on the Montreal Neurological Institute-152 brain template. Scatterplots show associations between handgrip strength residuals and residuals of (**b**) salience/ventral attention segregation and (**c**) mean salience/ventral attention intra-network functional connectivity, after controlling for age, sex, education and total intracranial volumes. Higher handgrip strength was linked to higher segregation of the salience/ventral attention network. Further examination of handgrip strength associations with salience/ventral attention inter- and intra-network functional connectivity revealed an association between higher handgrip strength and higher mean salience/ventral intra-network connectivity, suggesting that handgrip strength-related inter-individual differences in segregation of the salience/ventral attention network were driven by its intra-network connections. adj. adjusted, S/VA salience/ventral attention, FC functional connectivity, R right, L left, PI posterior insula, FO frontal operculum, AI anterior insula, MPPFC medial posterior prefrontal cortex.

Next, we examined how handgrip strength was linked to global and network-level functional brain segregation in older adults. Higher handgrip strength was associated with higher segregation of the salience/ventral attention network (FDR-adjusted *p* < 0.05, corrected for multiple comparisons across the nine network-level segregation measures) (Fig. 1a, b), but not global segregation or segregation of the other eight networks (Table 3). To understand the contributions of inter- and intra-network connections to the association between handgrip strength and salience/ventral attention network segregation, we investigated associations of handgrip strength with mean salience/ventral attention intra-network connectivity and mean salience/ventral attention inter-network connectivity to each of the other networks. Higher handgrip strength was significantly associated with higher salience/ventral attention intra-network functional connectivity (FDR-adjusted *p* < 0.05, corrected for multiple comparisons across the nine salience/ventral attention inter- and intra-network functional connectivity measures) (Table 4, Fig. 1c). We also found higher handgrip strength to be associated with lower inter-network connectivity between the salience/ventral attention and default networks as well as higher inter-network connectivity between the salience/ventral attention and somatomotor networks, although these did not pass multiple comparison correction (Table 4). Our findings thus suggest that handgrip strength-related segregation inter-individual differences in the salience/ventral attention network were driven primarily by functional connectivity inter-individual differences within the salience/ventral attention network.

**Table 3:** Associations between handgrip strength and global and network-level system segregation (*n* = 148)

Measure	Coefficient	SE	t	Uncorr p	FDR-adj p
Global segregation	1.69e^−3^	8.81e^−4^	1.92	0.0569	0.0569
Network segregation					
Default	2.17e^−3^	1.53e^−3^	1.42	0.1587	0.3571
Control	7.02e^−4^	1.63e^−3^	0.43	0.6679	0.7514
Limbic	1.41e^−3^	1.82e^−3^	0.77	0.4398	0.6598
Salience/ventral attention	4.90e^−3^	1.47e^−3^	3.32	0.0011*	0.0102*
Dorsal attention	1.01e^−3^	1.91e^−3^	0.53	0.5982	0.7514
Somatomotor	2.89e^−3^	1.68e^−3^	1.72	0.0880	0.3465
Visual	2.59e^−4^	1.08e^−3^	0.24	0.8101	0.8101
Temporoparietal	5.40e^−3^	3.41e^−3^	1.58	0.1155	0.3465
Subcortical	1.08e^−3^	1.23e^−3^	0.88	0.3808	0.6598

All models controlled for age, sex, education and total intracranial volumes. Multiple comparison correction was performed across the nine network-level segregation measures.

*SE* standard error, *Uncorr* uncorrected, *FDR-adj* false discovery rate-adjusted.

*indicates statistically significant effects (*p* < 0.05).

**Table 4:** Associations between handgrip strength and mean salience/ventral attention inter- and intra-network functional connectivity (*n* = 148)

Measure	Coefficient	SE	*t*	Uncorr p	FDR-adj p
Intra-network S/VA	4.29e^−3^	1.46e^−3^	2.94	0.0038*	0.0342*
Inter-network					
S/VA – Default	−2.82e^−3^	1.40e^−3^	−2.02	0.0456*	0.1369
S/VA – Control	−7.65e^−4^	1.07e^−3^	−0.72	0.4752	0.5379
S/VA – Limbic	−8.88e^−4^	1.44e^−3^	−0.62	0.5379	0.5379
S/VA – Dorsal attention	1.14e^−3^	1.36e^−3^	0.84	0.4032	0.5379
S/VA – Somatomotor	3.35e^−3^	1.42e^−3^	2.35	0.0200*	0.0898
S/VA – Visual	2.38e^−3^	1.81e^−3^	1.32	0.1891	0.4256
S/VA – Temporoparietal	1.23e^−3^	1.93e^−3^	0.64	0.5226	0.5379
S/VA – Subcortical	9.42e^−4^	9.23e^−4^	1.02	0.3091	0.5379

All models controlled for age, sex, education and total intracranial volumes. Multiple comparison correction was performed across the nine salience/ventral attention inter- and intra-network functional connectivity measures.

*SE* standard error, *Uncorr* uncorrected, *FDR-adj* false discovery rate-adjusted, *S/VA* salience/ventral attention.

*indicates statistically significant effects (*p* < 0.05).

**Fig. 2:**
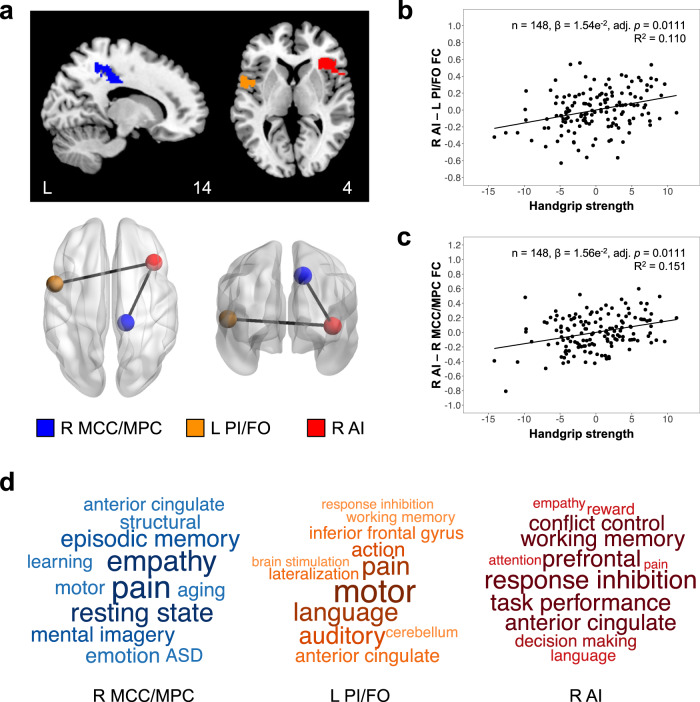
Higher handgrip strength is associated with higher right anterior insula functional connectivity to the left posterior insula/frontal operculum and right midcingulate/medial parietal cortex. **a** Brain map depicts the three brain regions of the salience/ventral attention network (blue: right midcingulate/medial parietal cortex; orange: left posterior insula/frontal operculum; red: right anterior insula) overlaid on the Montreal Neurological Institute-152 brain template. Glass brain displays regional connections between these three regions that show significant associations with handgrip strength. Scatterplots show associations between handgrip strength residuals and residuals of right anterior insula functional connectivity to (**b**) left posterior insula/frontal operculum and (**c**) right midcingulate/medial parietal cortex, after controlling for age, sex, education and total intracranial volumes. Within the salience/ventral attention network, higher handgrip strength was linked to higher functional connectivity of the right anterior insula to the left posterior insula/frontal operculum and right midcingulate/medial parietal cortex. **d** Word cloud displays the top topics from functional decoding analyses that were correlated with the right midcingulate/medial parietal cortex (blue), left posterior insula/frontal operculum (orange) and right anterior insula (red). Larger fonts and darker colours indicate stronger correlations. The right midcingulate/parietal medial cortex and left posterior insula/frontal operculum were associated with topics representing somatomotor functions (e.g., pain, motor) while the right anterior insula was associated with topics representing cognitive functions (e.g., response inhibition, task performance). adj. adjusted, FC functional connectivity, R right, L left, AI anterior insula, MCC midcingulate cortex, MPC medial parietal cortex, PI posterior insula, FO frontal operculum.

We then further sought to identify which brain regions contributed to handgrip strength-related inter-individual differences in segregation and intra-network connectivity of the salience/ventral attention network. Linear regression models examining associations between handgrip strength and each pair of regional connections (total: 231 pairs) within the salience/ventral attention network were conducted. Higher handgrip strength was significantly associated with higher functional connectivity of the right anterior insula to the left posterior insula/frontal operculum (Fig. 2b) and right midcingulate/medial parietal cortex (Fig. 2c) (all: FDR-adjusted *p* < 0.05, corrected for multiple comparisons across all 231 pairs of regional connections) (Table 5).

**Table 5:** Significant associations between handgrip strength and intra-network salience/ventral attention functional connections (*n* = 148)

Measure	Coefficient	SE	t	Uncorr p	FDR-adj p
Right AI – Left PI/FO	1.54e^−2^	3.82e^−3^	4.02	0.0001*	0.0111*
Right AI – Right MCC/MPC	1.56e^−2^	3.90e^−3^	4.01	0.0001*	0.0111*

All models controlled for age, sex, education and total intracranial volumes. Multiple comparison correction was performed across 231 pairs of regional connections within the salience/ventral attention network.

*SE* standard error, *Uncorr* uncorrected, *FDR-adj* false discovery rate-adjusted, *AI* anterior insula, *PI* posterior insula, *FO* frontal operculum, *MCC* midcingulate cortex, *MPC* medial parietal cortex.

*indicates statistically significant effects (*p* < 0.05).

All these findings remained after controlling for SM-MMSE scores, Geriatric Depression Scale scores, body mass index, waist-hip ratio, time interval between MRI scan and handgrip strength assessment, or total grey matter volumes (instead of total intracranial volumes) (Supplementary Data 1, 2, Supplementary Table 3).

### Functional decoding of salience/ventral attention network regions implicated in handgrip strength performance reveal links to cognitive and somatomotor functions

To examine the behavioural relevance of the three salience/ventral attention network regions (i.e., right anterior insula, left posterior insula/frontal operculum and right midcingulate/medial parietal cortex) whose regional connections were associated with handgrip strength, we performed meta-analytic functional decoding on the binarized brain maps of these regions (Fig. 2a). Functional decoding revealed that the right anterior insula was characterized by topics representing cognitive functions (e.g., response inhibition, task performance, prefrontal) (Fig. 2d, Supplementary Data 3), while the right midcingulate/medial parietal cortex and left posterior insula/frontal operculum were characterized by topics representing somatomotor functions (e.g., motor, pain) (Fig. 2d, Supplementary Data 4, 5).

### Handgrip strength-related inter-individual differences in salience/ventral attention network functional connectivity are associated with inter-individual differences in cognitive performance

**Fig. 3:**
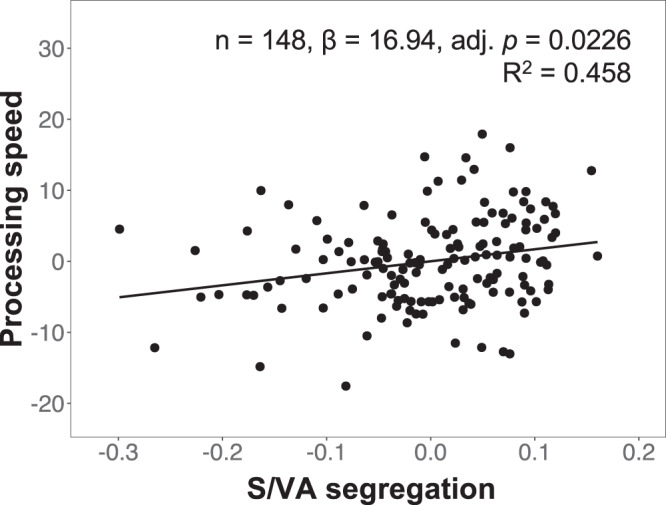
Higher salience/ventral attention network segregation is associated with better processing speed performance. Scatterplot depicts the association between processing speed residuals and residuals of salience/ventral attention network segregation after controlling for age, sex, education and total intracranial volumes. Higher salience/ventral attention network segregation was associated with better processing speed performance. adj. adjusted, S/VA salience/ventral attention.

Further, we investigated associations between inter-individual differences in handgrip strength-related functional connectivity and cognitive performance. Higher salience/ventral attention network segregation was associated with better performance in processing speed (FDR-adjusted *p* < 0.05, corrected for multiple comparisons across the five cognitive measures) (Fig. 3) and global cognition (uncorrected *p* < 0.05) (Table 6). Similarly, higher mean salience/ventral attention intra-network functional connectivity as well as higher right anterior insula connectivity to the left posterior insula/frontal operculum and right midcingulate/medial parietal cortex were associated with better processing speed (Supplementary Fig. 1) and global cognition performance (all: FDR-adjusted *p* < 0.05, corrected for multiple comparisons across the five cognitive measures) (Table 6). These findings remained after controlling for SM-MMSE scores, Geriatric Depression Scale scores, body mass index, waist-hip ratio, or total grey matter volumes (instead of total intracranial volumes) (Supplementary Data 6).

**Table 6:** Associations between cognitive performance and functional connectivity measures (*n* = 148)

FC measure	Cognitive measure	Coefficient	SE	t	Uncorr p	FDR-adj p
S/VA segregation	Global	9.08	4.29	2.12	0.0358*	0.0894
	Processing speed	16.94	5.87	2.88	0.0045*	0.0226*
	Attention	3.53	5.56	0.63	0.5266	0.6583
	Executive function	6.79	5.07	1.34	0.1825	0.3041
	SM-MMSE	0.77	1.96	0.39	0.6970	0.6970
Intra-network S/VA	Global	11.79	4.33	2.73	0.0072*	0.0180*
	Processing speed	19.25	5.94	3.24	0.0015*	0.0075*
	Attention	6.83	5.65	1.21	0.2285	0.2856
	Executive function	9.30	5.14	1.81	0.0724	0.1206
	SM-MMSE	1.49	2.00	0.74	0.4587	0.4587
R AI – L PI/FO	Global	4.20	1.61	2.61	0.0101*	0.0252*
	Processing speed	7.11	2.21	3.22	0.0016*	0.0081*
	Attention	2.49	2.10	1.19	0.2377	0.2972
	Executive function	3.01	1.92	1.57	0.1185	0.1974
	SM-MMSE	0.28	0.74	0.38	0.7068	0.7068
R AI – R MCC/MPC	Global	3.98	1.58	2.51	0.0131*	0.0328*
	Processing speed	5.82	2.19	2.66	0.0088*	0.0328*
	Attention	2.86	2.06	1.39	0.1667	0.2083
	Executive function	3.25	1.88	1.73	0.0850	0.1416
	SM-MMSE	0.91	0.73	1.26	0.2098	0.2098

All models controlled for age, sex, education and total intracranial volumes. For each functional connectivity measure, multiple comparison correction was performed across the five cognitive measures.

*FC* functional connectivity, *SE* standard error, *Uncorr* uncorrected, *FDR-adj* false discovery rate-adjusted, *SM-MMSE* Singapore-modified version of the mini-mental state examination, *S/VA* salience/ventral attention, *R* right, *L* left, *AI* anterior insula, *PI* posterior insula, *FO* frontal operculum, *MCC* midcingulate cortex, *MPC* medial parietal cortex.

*indicates statistically significant effects (*p* < 0.05).

### Mediation effect of salience/ventral attention network functional connectivity on the association between handgrip strength and cognitive performance

For functional connectivity measures that showed significant associations with both handgrip strength and cognitive performance, we additionally ran exploratory mediation analyses to examine if these functional connectivity measures mediated the relationship between handgrip strength and cognitive performance. We found a significant indirect effect of the right anterior insula functional connectivity to the left posterior insula/frontal operculum on the association between handgrip strength and processing speed (proportion of effect size mediated = 27.1%, *p* = 0.038) (Supplementary Data 7), indicating a partial mediation. Indirect effects of the other salience/ventral attention network functional connectivity measures (i.e., segregation, intra-network functional connectivity, and right anterior insula connectivity to the right midcingulate/medial parietal cortex) on the handgrip strength-processing speed association were also observed, however, these effects only showed a trend towards significance (proportion mediated = 20.4–21.5%, *p* = 0.063–0.102) (Supplementary Data 7). For the relationship between handgrip strength and global cognition, no significant indirect effects of the functional connectivity measures were found. These findings remained even after total grey matter volumes (instead of total intracranial volumes) were regressed out from the variables (Supplementary Data 8).

## Discussion

Our study sought to examine the relationships linking handgrip strength, brain functional connectivity and cognition in older adults. Unlike previous studies which focused on specific networks, we examined associations between handgrip strength and functional segregation in a whole-brain, network-agnostic manner, first identifying networks whose functional segregation were related to handgrip strength. Subsequently, for these identified networks, we sought to examine if handgrip strength-related inter-individual differences in functional segregation of the network were driven by specific inter- or intra-network functional connections. We demonstrated that higher handgrip strength was associated with better processing speed, global cognition and attention performance, as well as higher functional segregation of the salience/ventral attention network, but not other networks, in older adults. This higher handgrip strength-related segregation of the salience/ventral attention network was driven mainly by higher salience/ventral attention intra-network connectivity, particularly the functional connectivity of the right anterior insula to the left posterior insula/frontal operculum and right midcingulate/medial parietal cortex. Importantly, these inter-individual differences in functional connectivity that were related to handgrip strength also correlated with inter-individual differences in processing speed and global cognition performance. Further, the three brain regions which had functional connections associated with handgrip strength, i.e., the right anterior insula, left posterior insula/frontal operculum and right midcingulate/medial parietal cortex, were linked to topics representing cognitive and somatomotor functions. These findings remained even after additionally controlling for inter-individual differences in SM-MMSE scores, Geriatric Depression Scale scores, body mass index, waist-hip ratio or time interval between MRI scan and handgrip assessment. Moreover, exploratory analyses revealed some mediation effects of salience/ventral attention network functional connectivity, particularly the connectivity between the right anterior insula and the left posterior insula/frontal operculum, on the relationship between handgrip strength and processing speed. Our findings thus suggest that inter-individual differences in functional segregation and intra-network functional connectivity of the salience/ventral attention network could serve as the neural underpinning for the relationship between handgrip strength and cognition.

Our findings revealed an association between higher handgrip strength and higher salience/ventral attention functional network segregation in older adults that is driven primarily by higher intra-network functional connectivity of the right anterior insula. Previous studies examining motor or physical frailty-related inter-individual differences in functional connectivity have focused on the functional connectivity of specific regions or networks. In this study, we have instead examined how handgrip strength was related to both global and network-level functional segregation of all networks in the brain, which may provide greater insight on the neural basis of the relationship between handgrip strength and cognition. Functional segregation reflects the balance of dense connections between regions within a network and limited interaction with regions of other networks^34^, and provides a summary measure of the large-scale network organization of the brain^45^. Maintaining functional segregation of brain networks has been proposed to be important for brain function in several ways, including ensuring functional specialization of processing roles, conferring resilience and vulnerability to damage, and facilitating adaptation to processing demands^45^. Adding to the current literature on the neural correlates of handgrip strength, our findings suggest that inter-individual differences in handgrip strength in older adults were selectively related to functional connectivity inter-individual differences of specific networks (i.e., salience/ventral attention network), rather than global inter-individual differences across several functional networks. Specifically, our findings suggest that lower handgrip strength in older adults is related to a disruption in the balance of intra- and inter-network connections of the salience/ventral attention network, consequently resulting in lower functional specialization and poorer ability of the network to carry out its specialized function. Notably, by further examining handgrip strength-related inter-individual differences in intra- and inter-network connectivity, we revealed that this disruption in the balance between intra- and inter-network connections of the salience/ventral attention network was driven largely by disruptions in intra-network connectivity, along with some, albeit weaker (i.e., did not pass multiple comparison correction) disruptions of inter-network connectivity to default and somatomotor networks.

Motor performance has been suggested to require attention control^30^, which encompasses functions of response inhibition, distractor suppression and error monitoring^46^. To this end, the salience/ventral attention network, anchored in the anterior insula and dorsal anterior cingulate cortex, plays a key role in attention and cognitive control and contributes to a variety of brain functions (including motor functions) via the integration of cognitive, sensory and emotional information^47^. The anterior insula detects salient events through multiple sensory inputs and links with subcortical regions, and interacts with the posterior insula to integrate salient events with autonomic information^31,47–49^. The dorsal anterior cingulate cortex, on the other hand, enables response selection and motor response through its connections to the midcingulate cortex and other motor areas^31^. The salience/ventral attention network also acts as a switch between large-scale brain networks involved in internally oriented (i.e., default mode network) and externally oriented (i.e., central executive network) mental processes, guiding responses to salient stimuli by initiating appropriate control signals (through the anterior insula) that engage working memory, attentional and control processes while disengaging task-irrelevant systems^50^. Consistent with our finding showing the involvement of the salience/ventral attention functional network in handgrip strength, mean functional connectivity within the salience network and performance in an attention control task have been previously reported in an earlier study to relate to maximal grip force and tracking performance in both healthy and stroke participants^30^. Additionally, task-based fMRI studies have reported activation of salience network regions (e.g., insula and cingulate cortices) during grip tasks^51,52^. Moreover, we showed that higher regional connectivity of the right anterior insula to the left posterior insula/frontal operculum and right midcingulate/medial parietal cortex were specifically associated with higher handgrip strength. In line with the proposed role of the salience/ventral attention network in motor performance and attention control, functional decoding analyses revealed that this anterior insula region was linked to response inhibition and control, while the left posterior insula/frontal operculum and right midcingulate/medial parietal cortex regions were linked to somatomotor functions. Further, although preliminary at this juncture due to the cross-sectional nature of the study, we demonstrated that functional connectivity between the right anterior insula and the left posterior insula/frontal operculum significantly partially mediated the relationship between handgrip strength and processing speed, even after accounting for variations in total grey matter volume. Given that the mediation effects of other salience/ventral attention network functional connectivity measures also showed a trend towards significance, these findings indicate that inter-individual differences in salience/ventral attention network connectivity may possibly underpin the relationship between handgrip strength and cognition, although future longitudinal studies with larger sample sizes are required to confirm these mediation effects (particularly those with smaller effect sizes). Taken together, the findings therefore suggest that the functional integrity of the salience/ventral attention network and its attention/cognitive control function may be important for handgrip strength, and could serve as a neural mechanism underlying inter-individual differences in both handgrip strength and cognition in older individuals.

In particular, performance of an isometric handgrip exercise concurrently elicits spontaneous pupil diameter fluctuations^53,54^, which have been shown to track vigilance^55,56^. Vigilance, or tonic alertness, describes the intrinsic capacity to sustain attention for a period of time^57^ and is part of the alerting system, one of three systems (the other two being orienting and executive control) proposed by Posner and Petersen to be involved in attention^46,58^. Notably, tonic alertness has been proposed to be selectively subserved by the salience/ventral attention network and not the other task-positive networks such as the control or dorsal attention networks^57^. Additionally, a vigilance network has previously been identified, comprising regions from salience/ventral attention, somatomotor and default networks^59,60^. These findings align with our findings of handgrip strength associations with salience/ventral attention intra-network connectivity and to a lesser extent, salience/ventral attention inter-network connectivity to both the default and somatomotor networks, and hence suggest a possible link between handgrip strength and vigilance/tonic alertness that is mediated by changes in salience/ventral attention network connectivity. Furthermore, we did not find any associations between handgrip strength and salience/ventral attention inter-network connectivity to other networks, indicating that handgrip strength may be less related to other aspects of attention such as executive control, which involves both the salience/ventral attention network and other task-positive networks (e.g., control and dorsal attention networks)^46^. These conjectures are however preliminary, and more research needs to be done to examine the relationships between handgrip strength and various aspects of attention as well as the neural mechanisms underlying these relationships.

Importantly, our findings demonstrated that both handgrip strength and salience/ventral attention network functional connectivity were related to cognitive performance, highlighting the links between handgrip strength, cognition and salience/ventral attention network connectivity. Specifically, we found both handgrip strength and salience/ventral attention network functional connectivity to be associated with general cognition (an average measure of the three cognitive domains) and processing speed, but showed either weaker or no association with the other cognitive domains (i.e., attention and executive function). Processing speed, which measures the time taken to perform a mental task^61^, is among the strongest predictors of performance across various cognitive tasks^62^, and has been demonstrated to explain a large proportion of age-related variance in a wide range of cognitive tasks^63^. In a study examining handgrip strength associations with 30 behavioural outcomes in >40,000 participants from the UK Biobank, processing speed (measured as reaction time) was found to show the most robust association with handgrip strength^64^, which the authors partly attributed to the strong dependence of the reaction time task on motor speed and dexterity (both closely associated with the muscular function of the hands)^65^. Several studies have also demonstrated that short bouts of isometric handgrip exercises can temporarily improve reaction times in an auditory oddball task^54^, an auditory n-back task^53^ and a Go/No-Go task^66^, underscoring the link between handgrip strength and processing speed, as well as raising the possibility of training handgrip strength to improve processing speed. As for the salience/ventral attention network, lower salience/ventral attention network functional connectivity has been found to relate to age-related decreases in visual processing speed (i.e., the rate of visual information uptake)^67^. Interestingly, visual processing speed has been proposed to be closely linked to tonic alertness^68^. In a follow-up study, alertness training in healthy older adults was found to improve visual processing speed, with pre-training salience/ventral attention network functional connectivity correlated with the extent of training-induced change in visual processing speed^69^. These findings thus provide further support for the role of salience/ventral attention network functional connectivity and tonic alertness on both processing speed and handgrip strength. Collectively, these findings also raise the possibility that interventions targeting handgrip strength could potentially improve cognitive performance in older adults via modifications of salience/ventral attention network functional connectivity, although further intervention studies need to be done to evaluate the long-term effects of handgrip strength training on functional connectivity and cognitive performance. Notwithstanding, other studies have also reported performance in other cognitive domains (e.g., working memory, executive function) to be related to handgrip strength^15–19^ and salience/ventral attention network functional connectivity^70,71^. As such, more studies are needed to shed light on the neural mechanisms underlying the associations between handgrip strength and performance in various cognitive domains. It is possible that the more robust associations with processing speed are also partly explained by age-related changes in processing speed, given that processing speed is highly sensitive to ageing and has been widely demonstrated to show robust age-related declines^63,72,73^. Indeed, exploratory analyses revealed that age was most strongly associated with both processing speed and global cognition (Supplementary Table 4), suggesting possible influences of age on these associations. In our study, we have attempted to account for the influence of age in all our analyses, however future longitudinal studies might be more equipped to disentangle the influence of age on the handgrip strength-brain-cognition associations from other non-age-related sources.

Surprisingly, although previous studies have demonstrated associations between somatomotor network functional connectivity and handgrip strength^26,43,44^, we found only a trend towards a positive association between handgrip strength and somatomotor network segregation. The discrepancy in findings could likely be attributed to either differences in the type of functional connectivity measure examined (functional segregation in our study versus functional connectivity of specific networks in the previous study)^26^ or differences in the handgrip strength measure used^43,44^. With regards to the latter, although Cassady et al. reported an association between somatomotor network segregation and a handgrip strength factor (identified using factor analysis on several somatomotor measures), the handgrip strength factor examined in their studies included not only contributions from handgrip strength but also measures of endurance^43,44^. When handgrip strength was instead examined as a standalone measure, Cassady et al. similarly reported only a trend towards a positive relationship between somatomotor network segregation and handgrip strength^44^. Given that other studies have also found associations between somatomotor network functional connectivity and other somatomotor measures^27–29,43,44^, these findings collectively indicate a possibility that handgrip strength might be related to neural mechanisms that differ from that of other somatomotor functions. Further studies are needed to examine the differential brain functional and structural changes associated with changes in various somatomotor functions.

This study is limited in some aspects. First, while we demonstrated that functional connectivity of the salience/ventral attention network was associated with both handgrip strength and cognitive function, we could not ascertain the temporal or causal relationships between these factors. This is due to the cross-sectional design of our study, which utilized data of handgrip strength, cognition and brain scans measured in close proximity to one another in time. Currently, there is uncertainty regarding the directionality of the relationship between handgrip strength and cognition. Although several studies have shown a longitudinal effect of handgrip strength on future or rate of cognitive decline^6,18,64,74^, others have also reported bidirectional associations between handgrip strength and cognitive performance^75,76^, particularly processing speed^18,64^. Future longitudinal studies may be better equipped to clarify the directionality of the handgrip strength-cognition relationship, enable further investigations into how neuroimaging measures might mediate this relationship, as well as understand the effects of ageing on both handgrip strength and cognition. Second, we have focused only on handgrip strength and its associations with functional connectivity and cognition in this study. There are however several other measures of physical function, such as gait speed and timed-up-and-go tests, and studies have suggested that different physical function measures may have differential associations with cognitive performance in older adults^74,77,78^. Future studies examining the neural correlates of various physical functioning measures and their subsequent links to cognitive function could provide a more in-depth insight into the relationship between physical and cognitive function.

In conclusion, higher handgrip strength was related to higher cognitive performance and higher functional segregation of the salience/ventral attention network in older adults, driven primarily by higher salience/ventral attention intra-network connectivity to the right anterior insula. These inter-individual differences in functional connectivity that were related to handgrip strength were, in turn, linked to inter-individual differences in cognitive function, as demonstrated by meta-analytic functional decoding and brain-cognition association analyses. Our findings thus suggest that shared inter-individual differences in salience/ventral attention network segregation and intra-network functional connectivity could underlie inter-individual differences in handgrip strength and cognition in older adults.

## Methods

### Participants

We included 148 older Chinese adults from the third follow-up interviews of the prospective, population-based Singapore Chinese Health Study^79^ who had complete data from neuroimaging scans, neuropsychological assessments, and measurements of physical frailty (including handgrip strength). All participants were free of the following conditions: (a) current psychiatric conditions within the past 2 years, (b) hypertension (above 140/90), (c) diabetes (HbA1c > 9%), (d) history of central nervous system diseases (e.g., brain injury, stroke, dementia), (e) neoplastic condition, (f) organ disease (e.g., renal or liver impairment), or (g) self-reported sleep disorders, including insomnia and sleep apnea/ frequent snoring. All participants provided written informed consent at the start of the study, and the studies were approved by the Institutional Review Board of the National University of Singapore (NUS).

### Neuropsychological assessment

All participants completed the Singapore-modified version of the Mini-Mental State Examination (SM-MMSE)^80,81^, Geriatric Depression Scale (GDS) as well as a neuropsychological test battery within 3 months of the neuroimaging scan. The test battery comprised three cognitive domains using the following tests: (1) attention: Wechsler Memory Scale-Third Edition (WMS-III) Digit Span and Spatial Span tests^82^, (2) processing speed: Symbol Digit Modalities Test^83^, WMS-III Symbol Search Task^82^ and Trail Making Test A^84^, as well as (3) executive function: Categorical Verbal Fluency Test^85^, Delis–Kaplan Executive Function System (D-KEFS) Design Fluency Test^86^, and Trail Making Test B^84^. For each test, individual scores were first z-transformed (using mean and standard deviation across all participants’ scores) and then subsequently converted to T-scores with a mean of 50 and standard deviation of 10. A participant who achieves a T-score of 60 on a cognitive test thus scored higher than approximately 84% of the participants in the study (equivalent to one standard deviation above the mean score across all participants). Composite scores for each of the three domains (attention, processing speed and executive function) were then obtained by averaging T-scores across all tests within each domain. Additionally, global cognition scores were obtained for each participant by averaging across the composite scores of the three domains.

### Handgrip strength assessment

Physical frailty measurements, including handgrip strength, were collected within 18 months of the neuroimaging and neuropsychological assessments (detailed protocol is described in previous work^10,87^). Handgrip strength measurements were taken by trained staff in the homes of the participants using the Takei hand grip dynamometer (Model TKK5401 Grip D) in accordance with standard protocols^10^. Participants first stood upright and held the handgrip dynamometer with their arms let down naturally, then subsequently squeezed the dynamometer with full force. Measurements were recorded to the nearest 0.1 kg. Two trials were performed for each hand, and only the highest value obtained from each hand was taken. Overall handgrip strength was calculated as the average of the measurements from the left and right hands.

### Image acquisition

Brain MRI scans on participants were performed at the Centre for Cognitive Neuroscience, Duke-NUS Medical School, Singapore using a 20-channel head coil on a 3-Tesla Siemens Magnetom Prisma Fit scanner. The scan protocol included a T1-weighted scan (magnetization prepared rapid gradient echo (MPRAGE) sequence, repetition time = 2300 ms, echo time = 2.98 ms, inversion time = 900 ms, flip angle = 9°, field of view = 256 × 256 mm^2^, voxel size = 1.0 × 1.0 × 1.0 mm^3^), was well as an 8-min T2*-weighted resting state fMRI scan (echo planar sequence, 36 axial slices, repetition time = 2000 ms, echo time = 30 ms, flip angle = 90°, field of view = 192 × 192 mm^2^, voxel size = 3.0 × 3.0 × 3.0 mm^3^, interleaved collection) where participants were instructed to fixate at a crosshair in the centre of the screen.

### Functional image preprocessing

Resting state fMRI images were preprocessed using the FMRIB (Oxford Centre for Functional MRI of the Brain) Software Library (FSL)^88^ and Analysis of Functional NeuroImages software^89^ following previous procedures^42^. The preprocessing steps were as follows: (1) removal of first five volumes for magnetic field stabilization; (2) motion and slice timing correction; (3) time series despiking; (4) grand mean scaling; (5) spatial smoothing with a 6 mm Gaussian kernel, (6) temporal band-pass frequency filtering in the range of 0.009 to 0.1 Hz; (7) removal of linear and quadratic trends; (8) co-registration of T1-weighted image using boundary based registration, followed by non-linear registration of fMRI image to Montreal Neurological Institute 152 (MNI152) space using FNIRT; and (9) regression of nine nuisance signals (global signal, white matter, cerebrospinal fluid and six motion parameters) from the preprocessed fMRI images. All images met satisfactory motion criteria (maximum relative displacement ≤1 mm and maximum absolute displacement ≤4 mm).

### Structural image preprocessing

Voxelwise grey matter volume probability maps were derived from T1-weighted images using voxel-based morphometry (VBM). VBM was performed using the computational anatomy toolbox (CAT12 Structural Brain Mapping Group; http://www.neuro.uni-jena.de/cat/) for Statistical Parametric Mapping (SPM12; Wellcome Trust Centre for Neuroimaging; http://www.fil.ion.ucl.ac.uk/spm/software/spm12/). The steps were as follows: (1) bias correction and affine registration of T1-weighted images; (2) initial segmentation of images into grey matter, white matter and cerebrospinal fluid using unified SPM segmentation^90^, followed by segmentation using the adaptive Maximum A Posterior^91^ method which eliminates the need for tissue priors; (3) study-specific template creation using DARTEL (Diffeomophic Anatomical Registration Through Exponentiated Lie Algebra) registration of the affine-registered grey and white matter segmented images^92^; (4) registration of grey and white matter probability maps to the study-specific template in MNI152 space; (5) modulation of voxel values with both linear and non-linear components of the Jacobian determinant to allow for comparison of absolute tissue volumes; and finally (6) smoothing of the modulated normalized grey matter images using an isotropic 8 mm Gaussian kernel.

### Derivation of functional connectivity matrices

Weighted, undirected functional connectivity matrices were derived for each participant. Mean BOLD time series of 144 regions-of-interest (ROIs) (114 cortical regions^93^, 30 subcortical regions^94,95^) were first extracted from individual preprocessed fMRI images in MNI152 space. Two cortical ROIs were subsequently dropped due to poor brain coverage, resulting in a total of 142 ROIs (see Supplementary Data 9 for the list of ROIs). The remaining 142 regions were grouped into nine networks: default, control, limbic, salience/ventral attention, dorsal attention, somatomotor, visual, temporoparietal and subcortical. Functional connectivity matrices (142 × 142 ROIs) were then obtained by computing the Pearson correlation between the time courses of each pair of ROIs, and transforming the correlation values to z-scores using Fisher’s r-to-z-transformation.

### Assessment of functional segregation

We computed global and network-level measures of system segregation introduced by ref. ^34^, which quantifies differences in within-network connections relative to between-network connections. First, all diagonal elements and negative connections in the functional connectivity matrices were set to 0. Next, for each network, we computed the mean within-network connectivity ($\bar{{{\mbox{Z}}}}$_w_$,\,$_network_) (e.g., for control network, this refers to the average of z-scores between all control network regions and all other control network regions) as well as the mean between-network connectivity ($\bar{{{\mbox{Z}}}}$_b_$,\,$_network_) (e.g., for control network, this refers to the average of z-scores between all control network regions and all other regions in the brain). Global mean within-network connectivity ($\bar{{{\mbox{Z}}}}$_w_$,$_global_) (average of z-scores of all within-network connections across networks) and mean between-network connectivity ($\bar{{{\mbox{Z}}}}$_b_$,\,$_global_) (average of z-scores of all between-network connections across networks) were also obtained. Finally, we calculated functional segregation at the global and network-level as follows:

$${{{{{\rm{Segregation}}}}}}=\frac{{\bar{{{{{\mathrm{Z}}}}}}}_{{{{{\mathrm{w}}}}}}-{\bar{{{{{\mathrm{Z}}}}}}}_{{{{{\mathrm{b}}}}}}}{{\bar{{{{{\mathrm{Z}}}}}}}_{{{{{\mathrm{w}}}}}}}$$

### Associations of handgrip strength with cognitive performance and measures of functional connectivity

Associations of handgrip strength with cognitive performance (SM-MMSE scores as well as composite scores for global cognition, processing speed, attention and executive function) and system segregation (global and nine network-level measures (pertaining to each of the nine brain networks)) were examined using linear regression models with handgrip strength as predictor and cognitive or segregation measure as outcome variable. To examine the contributions of intra- and inter-network connections to handgrip strength-related inter-individual differences in segregation, we additionally ran linear regression models with handgrip strength as predictor and intra-network/inter-network functional connectivity as outcome variable. For models examining handgrip strength-cognition associations, age, sex and education were included as nuisance covariates, while for models examining functional connectivity-cognition associations, age, sex, education, and total intracranial volumes (to account for total brain sizes) were included as nuisance covariates. The linear regression analyses were performed using R 4.04^96^ on Rstudio^97^. *P*-values were corrected for multiple comparisons using false discovery rate (FDR).

### Functional decoding of brain regions implicated in handgrip strength

To understand the cognitive and behavioural relevance of brain regions whose functional connections showed associations with handgrip strength, we performed topic-based meta-analytic functional decoding on the binarized map of each brain region using a set of 50 topics extracted with latent Dirichlet allocation topic modelling on the abstracts of all articles in the Neurosynth database^98,99^. This resulted in a total of three functional decoding analyses performed in the study, with separate analyses done for each of the three salience/ventral attention regions whose regional connections showed correlations with handgrip strength: right anterior insula, left posterior insula/frontal operculum and right midcingulate/medial parietal cortex. Specifically, we used the Neurosynth ROI association approach on NiMARE v0.0.12 package for Python^100,101^, which correlates the mean modelled activation values within the binarized ROI with the term weights of all 50 topics in the Neurosynth dataset. Visualization of these correlation values as word clouds were then performed using the wordcloud package on R^102^.

### Associations of functional connectivity measures with cognitive performance

For measures of functional connectivity that were found to be associated with handgrip strength, we further examined their associations with cognitive performance (SM-MMSE scores as well as composite scores for global cognition, processing speed, attention and executive function). Linear regression models were performed in the same manner as the associations with handgrip strength. For these models, we used cognitive score as outcome variable, functional connectivity measure as predictor, and age, sex, education, and total intracranial volumes (to account for total brain sizes) as nuisance covariates. *P*-values were corrected for multiple comparisons using FDR.

### Mediation effects of functional connectivity on the association between handgrip strength and cognitive performance

As an exploratory analysis, for functional connectivity measures that showed significant associations with both handgrip strength and cognitive performance, we examined if such functional connectivity measures mediated the relationship between handgrip strength and cognitive performance. Given that several studies have previously reported a longitudinal effect of handgrip strength on future or rate of cognitive decline^6,18,64,74^, mediation analyses were conducted with functional connectivity measure as the mediating variable, cognitive score as the dependent variable and handgrip strength as the independent variable. Age, sex, education, and total intracranial volume were first regressed out from the mediating, independent and dependent variables, and the residuals of these variables were subsequently used in the mediation analyses. Additionally, given that grey matter volumes have been associated with handgrip strength^64^, we repeated the mediation analyses with total grey matter volumes (instead of total intracranial volumes) regressed out from the mediating, dependent and independent variables. All mediation analyses were conducted using the ‘lavaan’ package on R^103^, and the significance of the indirect effects was evaluated using bootstrapped (5000 bootstrap iterations) confidence intervals.

### Validation analyses

Validation analyses were also conducted to determine the robustness of the findings (Supplementary Table 1). To ensure that the findings were not influenced by the extent of cognitive impairment in participants, we repeated the analyses with SM-MMSE scores included as an additional covariate. Additionally, handgrip strength has been shown to be associated with depressive symptoms^104,105^ as well as measures of obesity such as body mass index^106–109^. To ensure that the findings were not influenced by depressive symptoms or indices of obesity, we repeated the analyses with Geriatric Depression Scale scores, body mass index or waist-hip ratio included as an additional covariate. Further, handgrip strength assessments were found to be conducted on average 276.56 (SD = 156.64, range = −20–547) days after the MRI scans (Supplementary Fig. 2). Given this time difference between the MRI scan and handgrip assessment, we repeated the handgrip strength-functional connectivity association analyses with time interval (days) between the MRI scan and handgrip strength assessment included as an additional covariate. Lastly, to ensure that functional connectivity associations with handgrip strength and cognition were not influenced by variations in total grey matter volumes, we repeated the functional connectivity analyses with total grey matter volumes (instead of total intracranial volumes) included as a covariate.

### Statistics and reproducibility

Linear regression models were performed to examine associations of handgrip strength with cognitive performance and functional connectivity, as well as associations of functional connectivity with cognitive performance (*n* = 148). *P*-values were corrected for multiple comparisons using false discovery rate. Mediation analyses were also conducted to examine the mediation effects of functional connectivity on the handgrip strength-cognition association, with the significance of the indirect effects evaluated using bootstrapped (5000 bootstrap iterations) confidence intervals (*n* = 148). To examine the reproducibility of the results, we repeated the analyses: (1) controlling for SM-MMSE scores, (2) controlling for Geriatric Depression Scale scores, (3) controlling for body mass index, (4) controlling for waist-hip ratio, (5) controlling for the time interval between the MRI scan and handgrip strength assessment, and (6) controlling for total grey matter volumes (instead of total intracranial volumes).

### Reporting summary

Further information on research design is available in the Nature Portfolio Reporting Summary linked to this article.

### Supplementary information

Peer Review File

Supplementary Information

Description of Additional Supplementary Files

Supplementary Data 1

Supplementary Data 2

Supplementary Data 3

Supplementary Data 4

Supplementary Data 5

Supplementary Data 6

Supplementary Data 7

Supplementary Data 8

Supplementary Data 9

Reporting Summary

## Data Availability

The data that support the findings of this study are available from the authors but restrictions apply to the availability of these data due to Institutional Review Board stipulations and so are not publicly available. Data are, however, available from the authors upon reasonable request and with permission from National University of Singapore.

## Code availability

Custom Matlab, R and Python code supporting this manuscript can be assessed at https://github.com/hzlab/2023_Chong_CommunBiol_Gripstrength_FC and 10.5281/zenodo.10500670^110^.
