# Supplementary material for: Higher handgrip strength is linked to higher salience ventral attention functional network segregation in older adults
Source: Commun Biol. 2024 Feb 21;7:214. doi: 10.1038/s42003-024-05862-x (PMC10881588; doi:10.1038/s42003-024-05862-x)
Supplement: Supplementary file 2 — Supplementary Information [file 42003_2024_5862_MOESM2_ESM.pdf]

Supplementary Figure 1: Higher salience/ventral attention intra-network functional connectivity, particularly that of the right anterior insula, is associated with better processing speed performance.

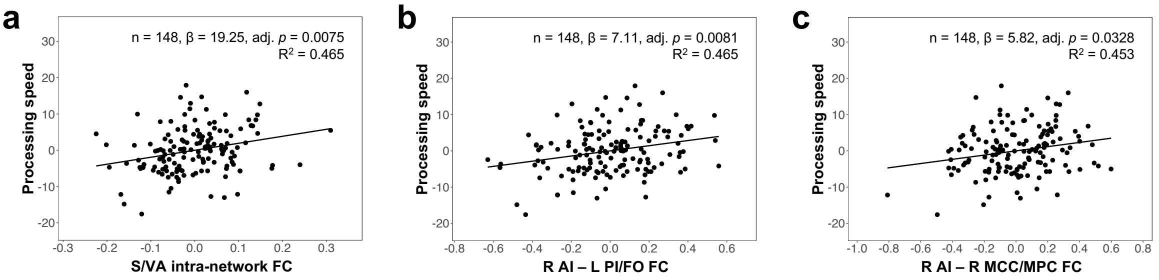

Scatterplots show associations between processing speed residuals and residuals of (a) mean salience/ventral attention intra-network functional connectivity, as well as right anterior insula functional connectivity to the (b) left posterior insula/frontal operculum and (c) right midcingulate/medial parietal cortex, after controlling for age, sex, education and total intracranial volumes. Better processing speed performance was associated with higher mean salience/ventral attention intra-network functional connectivity, as well as higher right anterior insula functional connectivity to the left posterior insula/frontal operculum and right midcingulate/medial parietal cortex. Abbreviations: adj., adjusted; S/VA, salience/ventral attention; FC, functional connectivity; R, right; L, left; AI, anterior insula; MCC, midcingulate cortex; MPC, medial parietal cortex; PI, posterior insula; FO, frontal operculum.

Supplementary Figure 2: Time interval between date of MRI scan and date of handgrip strength assessments.

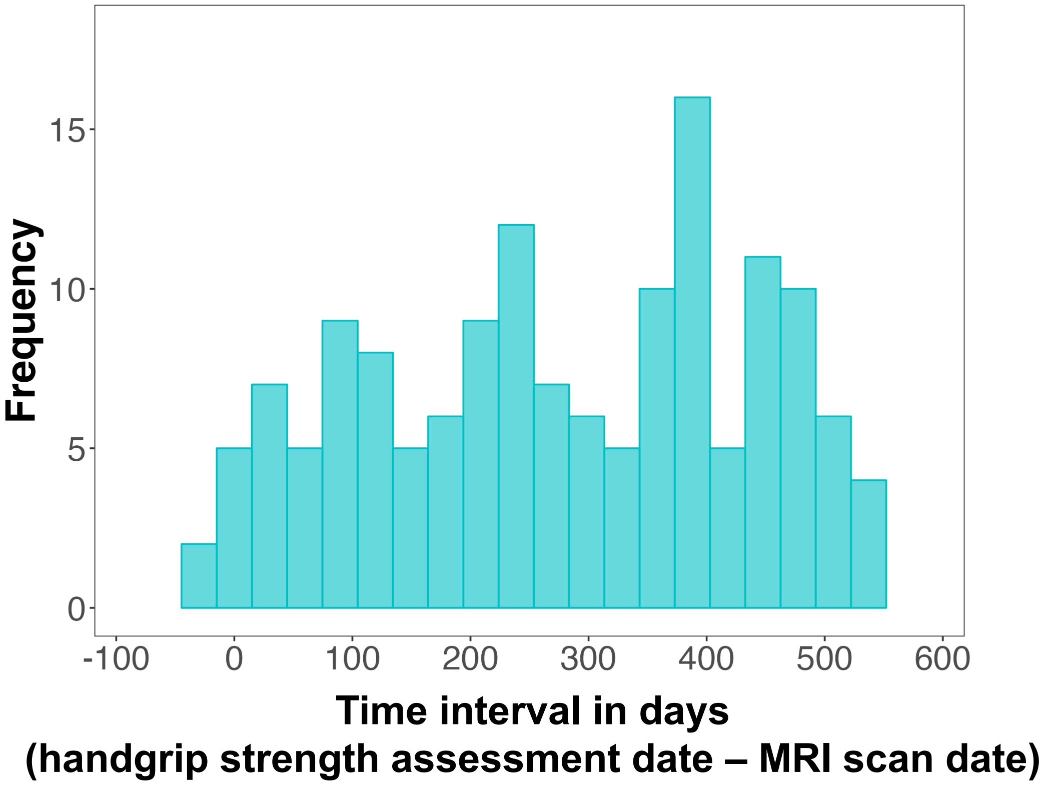

Histogram displays the time interval (in days) between the MRI scan and handgrip strength assessment (handgrip strength assessment date – MRI scan date). On average, handgrip strength assessments were performed 276.56 (SD = 156.64, range = -20 – 547) days after the MRI scans.

Supplementary Table 1: List of validation analyses performed

Analysis	Dataset	Nuisance covariates included
1	Original dataset (n = 148)	Age, sex, education, TIV and SM-MMSE
2	Original dataset (n = 148)	Age, sex, education, TIV and waist-hip ratio
3	Original dataset, excluding 2 subjects with missing body mass index values (n = 146)	Age, sex, education, TIV and body mass index
4	Original dataset (n = 148)	Age, sex, education, TIV and GDS scores
5[#]	Original dataset (n = 148)	Age, sex, education, TIV and time interval between MRI scan and handgrip strength measurement
6*	Original dataset (n = 148)	Age, sex, education and total grey matter volumes

[#]For analysis 5, only associations between handgrip strength and functional connectivity measures were examined. *For analysis 6, only associations between handgrip strength and functional connectivity measures, and associations between functional connectivity measures and cognitive performance were examined. Abbreviations: TIV, total intracranial volumes; SM-MMSE, Singapore-modified version of the mini-mental state examination; GDS, geriatric depression scale.

Supplementary Table 2: Validation analyses: associations between handgrip strength and cognitive measures

Cognitive measure	Coefficient	SE	*t*	Uncorr *p*	FDR-adj *p*
**Analysis 1: Analysis additionally controlling for SM-MMSE (n = 148)**					
Global	0.222	0.073	3.04	0.0028*	0.0102*
Processing speed	0.293	0.103	2.85	0.0051*	0.0102*
Attention	0.208	0.096	2.16	0.0327*	0.0436*
Executive function	0.165	0.090	1.83	0.0689	0.0689
**Analysis 2: Analysis additionally controlling for waist-hip ratio (n = 148)**					
Global	0.258	0.077	3.37	0.0010*	0.0049*
Processing speed	0.314	0.106	2.95	0.0037*	0.0093*
Attention	0.250	0.099	2.53	0.0126*	0.0211*
Executive function	0.210	0.091	2.31	0.0221*	0.0277*
SM-MMSE	0.045	0.036	1.25	0.2126	0.2126
**Analysis 3: Analysis additionally controlling for body mass index (n = 146)**					
Global	0.250	0.082	3.05	0.0027*	0.0135*
Processing speed	0.305	0.113	2.70	0.0078*	0.0195*
Attention	0.271	0.106	2.56	0.0117*	0.0195*
Executive function	0.175	0.098	1.79	0.0761	0.0952
SM-MMSE	0.048	0.037	1.27	0.2061	0.2061
**Analysis 4: Analysis additionally controlling for GDS scores (n = 148)**					
Global	0.248	0.076	3.27	0.0014*	0.0065*
Processing speed	0.322	0.105	3.06	0.0026*	0.0065*
Attention	0.237	0.099	2.40	0.0178*	0.0297*
Executive function	0.185	0.091	2.03	0.0440*	0.0550
SM-MMSE	0.042	0.035	1.20	0.2308	0.2308

All models controlled for age, sex and education. Multiple comparison correction was performed across the five cognitive measures. * indicates statistically significant effects ($p < 0.05$). Abbreviations: SE, standard error; Uncorr, uncorrected; FDR-adj, false discovery rate-adjusted; GDS, geriatric depression scale; SM-MMSE, Singapore-modified version of the mini-mental state examination.

Supplementary Table 3: Validation analyses: significant associations between handgrip strength and intra-network salience/ventral attention functional connections

Measure	Coefficient	SE	*t*	Uncorr *p*	FDR-adj *p*
**Analysis 1: Analysis additionally controlling for SM-MMSE (n = 148)**					
Right AI – Left PI/FO	$1.54e^{-2}$	$3.86e^{-3}$	3.99	0.0001*	0.0177*
Right AI – Right MCC/MPC	$1.53e^{-2}$	$3.92e^{-3}$	3.89	0.0002*	0.0177*
**Analysis 2: Analysis additionally controlling for waist-hip ratio (n = 148)**					
Right AI – Left PI/FO	$1.62e^{-2}$	$3.81e^{-3}$	4.24	<0.0001*	0.0046*
Right AI – Right MCC/MPC	$1.66e^{-2}$	$3.87e^{-3}$	4.30	<0.0001*	0.0046*
**Analysis 3: Analysis additionally controlling for body mass index (n = 146)**					
Right AI – Left PI/FO	$1.57e^{-2}$	$4.09e^{-3}$	3.84	0.0002*	0.0191*
Right AI – Right MCC/MPC	$1.58e^{-2}$	$4.11e^{-3}$	3.84	0.0002*	0.0191*
**Analysis 4: Analysis additionally controlling for GDS scores (n = 148)**					
Right AI – Left PI/FO	$1.53e^{-2}$	$3.83e^{-3}$	4.00	0.0001*	0.0126*
Right AI – Right MCC/MPC	$1.55e^{-2}$	$3.90e^{-3}$	3.98	0.0001*	0.0126*
**Analysis 5: Analysis additionally controlling for time interval between MRI scan and handgrip strength measurement (n = 148)**					
Right AI – Left PI/FO	$1.44e^{-2}$	$3.89e^{-3}$	3.70	0.0003*	0.0358*
Right AI – Right MCC/MPC	$1.51e^{-2}$	$3.98e^{-3}$	3.79	0.0002*	0.0358*
**Analysis 6: Analysis controlling for total grey matter volumes instead of TIV (n = 148)**					
Right AI – Left PI/FO	$1.43e^{-2}$	$3.96e^{-3}$	3.62	0.0004*	0.0468*
Right AI – Right MCC/MPC	$1.74e^{-2}$	$4.07e^{-3}$	4.27	<0.0001*	0.0084*

Multiple comparison correction was performed across 231 pairs of regional connections within the salience/ventral attention network. * indicates statistically significant effects ($p < 0.05$). Abbreviations: SE, standard error; Uncorr, uncorrected; FDR-adj, false discovery rate-adjusted; SM-MMSE, Singapore-modified version of the mini-mental state examination; GDS, geriatric depression scale; AI, anterior insula; PI, posterior insula; FO, frontal operculum; MCC, midcingulate cortex; MPC, medial parietal cortex; TIV, total intracranial volumes.

Supplementary Table 4: Associations between age and cognitive measures (n = 148)

Cognitive measure	Coefficient	SE	*t*	Uncorr *p*	FDR-adj *p*
Global	-0.502	0.103	-4.86	<0.0001*	<0.0001*
Processing speed	-0.665	0.143	-4.65	<0.0001*	<0.0001*
Attention	-0.349	0.132	-2.65	0.0090*	0.0112*
Executive function	-0.493	0.121	-4.08	0.0001*	0.0001*
SM-MMSE	-0.110	0.046	-2.37	0.0192*	0.0192*

All models controlled for sex and education. Multiple comparison correction was performed across the five cognitive measures. * indicates statistically significant effects ($p < 0.05$). Abbreviations: SE, standard error; Uncorr, uncorrected; FDR-adj, false discovery rate-adjusted; SM-MMSE, Singapore-modified version of the mini-mental state examination.
