# Supplementary material for: Higher handgrip strength is linked to higher salience ventral attention functional network segregation in older adults
Source: Commun Biol. 2024 Feb 21;7:214. doi: 10.1038/s42003-024-05862-x (PMC10881588; doi:10.1038/s42003-024-05862-x)
Supplement: Supplementary file 3 — Description of Additional Supplementary Files [file 42003_2024_5862_MOESM3_ESM.pdf]

Description of Additional Supplementary Files

File name: Supplementary Data 1
Description: Regions-of-interest used to compute functional connectivity measures.

File name: Supplementary Data 2
Description: Validation analyses: associations between handgrip strength and global and network-level system segregation.

File name: Supplementary Data 3
Description: Validation analyses: associations between handgrip strength and mean salience/ventral attention inter- and intra-network functional connectivity.

File name: Supplementary Data 4
Description: Correlation values of the right anterior insula with the term weights of 50 topics in the Neurosynth database.

File name: Supplementary Data 5
Description: Correlation values of the right midcingulate/medial parietal cortex with the term weights of 50 topics in the Neurosynth database.

File name: Supplementary Data 6
Description: Correlation values of the left posterior insula/frontal operculum with the term weights of 50 topics in the Neurosynth database.

File name: Supplementary Data 7
Description: Validation analyses: associations between cognitive performance and functional connectivity measures.

File name: Supplementary Data 8
Description: Mediation effects of functional connectivity measures on the association between handgrip strength and cognitive performance (controlling for total intracranial volumes).

File name: Supplementary Data 9
Description: Mediation effects of functional connectivity measures on the association between handgrip strength and cognitive performance (controlling for total grey matter volumes).
