# Supplementary material for: Higher handgrip strength is linked to higher salience ventral attention functional network segregation in older adults
Source: Commun Biol. 2024 Feb 21;7:214. doi: 10.1038/s42003-024-05862-x (PMC10881588; doi:10.1038/s42003-024-05862-x)
Supplement: Supplementary file 13 — Reporting Summary [file 42003_2024_5862_MOESM13_ESM.pdf]

nature portfolio

Corresponding author(s): Helen Juan Zhou

Last updated by author(s): Jan 15, 2024

Reporting Summary

Nature Portfolio wishes to improve the reproducibility of the work that we publish. This form provides structure for consistency and transparency in reporting. For further information on Nature Portfolio policies, see our Editorial Policies and the Editorial Policy Checklist.

Statistics

For all statistical analyses, confirm that the following items are present in the figure legend, table legend, main text, or Methods section.

n/a	Confirmed	
☐	☒	The exact sample size (*n*) for each experimental group/condition, given as a discrete number and unit of measurement
☐	☒	A statement on whether measurements were taken from distinct samples or whether the same sample was measured repeatedly
☐	☒	The statistical test(s) used AND whether they are one- or two-sided<br>*Only common tests should be described solely by name; describe more complex techniques in the Methods section.*
☐	☒	A description of all covariates tested
☐	☒	A description of any assumptions or corrections, such as tests of normality and adjustment for multiple comparisons
☐	☒	A full description of the statistical parameters including central tendency (e.g. means) or other basic estimates (e.g. regression coefficient) AND variation (e.g. standard deviation) or associated estimates of uncertainty (e.g. confidence intervals)
☐	☒	For null hypothesis testing, the test statistic (e.g. *F*, *t*, *r*) with confidence intervals, effect sizes, degrees of freedom and *P* value noted<br>*Give P values as exact values whenever suitable.*
☒	☐	For Bayesian analysis, information on the choice of priors and Markov chain Monte Carlo settings
☒	☐	For hierarchical and complex designs, identification of the appropriate level for tests and full reporting of outcomes
☐	☒	Estimates of effect sizes (e.g. Cohen's *d*, Pearson's *r*), indicating how they were calculated

Our web collection on statistics for biologists contains articles on many of the points above.

Software and code

Policy information about availability of computer code

Data collection	No software was used.
Data analysis	Resting state fMRI images were preprocessed using the FMRIB (Oxford Centre for Functional MRI of the Brain) Software Library (FSL) and Analysis of Functional NeuroImages software.<br>Voxel based morphometry on T1-weighted MRI images was performed using the computational anatomy toolbox (CAT12 Structural Brain Mapping Group; http://www.neuro.uni-jena.de/cat/) for Statistical Parametric Mapping on MATLAB.<br>Functional connectivity measures were calculated using custom scripts on MATLAB.<br>Statistical analyses (linear regression models and mediation models) were performed using R 4.04 on Rstudio. The lavaan library in R was used to run the mediation models.<br>Functional decoding of brain regions were performed using the NiMARE v0.012 package on Python.<br>For the figures, scatterplots were created using the ggplot2 library on R, word cloud was created using wordcloud library on R, brain maps were visualized using both MRIcron software and the BrainNet Viewer toolbox on MATLAB.

For manuscripts utilizing custom algorithms or software that are central to the research but not yet described in published literature, software must be made available to editors and reviewers. We strongly encourage code deposition in a community repository (e.g. GitHub). See the Nature Portfolio guidelines for submitting code & software for further information.

Data

Policy information about availability of data

All manuscripts must include a data availability statement. This statement should provide the following information, where applicable:

- Accession codes, unique identifiers, or web links for publicly available datasets
- A description of any restrictions on data availability
- For clinical datasets or third party data, please ensure that the statement adheres to our policy

The data supporting this manuscript is available upon request from the corresponding author. The data is not publicly available due to institute policy.

Research involving human participants, their data, or biological material

Policy information about studies with human participants or human data. See also policy information about sex, gender (identity/presentation), and sexual orientation and race, ethnicity and racism.

Reporting on sex and gender	Sex of participants was determined by self-report. Sex is included as a covariate in the data analyses. In total, there were 83 females and 65 males included in the study.
Reporting on race, ethnicity, or other socially relevant groupings	Ethnicity of participants was determined by self-report. Participants in this study are all Chinese and were recruited as part of the Singapore Chinese Health Study, a population-based prospective epidemiology cohort study in Singapore.
Population characteristics	Age, years of education, and sex were collected and included as covariates in the data analyses.
Recruitment	Participants were recruited based on the following exclusion criteria: a) current psychiatric conditions within the past two years, b) hypertension (above 140/90), c) diabetes (HbA1c > 9%), d) history of central nervous system diseases (e.g., brain injury, stroke, dementia), e) neoplastic condition, f) organ disease (e.g., renal or liver impairment), or g) self-reported sleep disorders, including insomnia and sleep apnea/ frequent snoring.
Ethics oversight	Institutional Review Board of the National University of Singapore

Note that full information on the approval of the study protocol must also be provided in the manuscript.

Field-specific reporting

Please select the one below that is the best fit for your research. If you are not sure, read the appropriate sections before making your selection.

☒ Life sciences ☐ Behavioural & social sciences ☐ Ecological, evolutionary & environmental sciences

For a reference copy of the document with all sections, see nature.com/documents/nr-reporting-summary-flat.pdf

Life sciences study design

All studies must disclose on these points even when the disclosure is negative.

Sample size	Sample sizes of previous studies reporting associations between functional connectivity and the physical frailty phenotype (Fried's frailty phenotype) or its components (e.g., handgrip strength, gait speed) ranged from 22 to 193 participants (Cassady et al., 2019; Seidler et al., 2015; Yuan et al., 2015; Hirsiger et al., 2016; Lammers et al., 2020). Additionally, based on a pilot sample of 20 subjects, a sample size of 130 was calculated to achieve a power of 90% at a significance level of α=0.05 (two-sided), given an effect size of 0.082 in a linear regression model of handgrip strength against salience/ventral attention network segregation.
Data exclusions	200 participants underwent MRI scans. Of the 200 participants, 52 were excluded from the current study due to poor quality MRI scans or missing neuropsychological assessments or handgrip strength data.
Replication	Replication of this study's findings is currently ongoing.
Randomization	Randomization is not relevant to our study as this is an observational study of community-dwelling older adults and participants were not allocated into experimental groups.
Blinding	Blinding is not relevant to our study as as this is an observational study of community-dwelling older adults and participants were not allocated into experimental groups.

Reporting for specific materials, systems and methods

We require information from authors about some types of materials, experimental systems and methods used in many studies. Here, indicate whether each material, system or method listed is relevant to your study. If you are not sure if a list item applies to your research, read the appropriate section before selecting a response.

Materials & experimental systems

n/a	Involved in the study
☒	☐ Antibodies
☒	☐ Eukaryotic cell lines
☒	☐ Palaeontology and archaeology
☒	☐ Animals and other organisms
☒	☐ Clinical data
☒	☐ Dual use research of concern
☒	☐ Plants

Methods

n/a	Involved in the study
☒	☐ ChIP-seq
☒	☐ Flow cytometry
☐	☒ MRI-based neuroimaging

Magnetic resonance imaging

Experimental design

Design type: Resting state

Design specifications: Each participant underwent one 8-min resting state fMRI scan, where they were instructed to fixate at a crosshair in the centre of the screen.

Behavioral performance measures: Given that this is a resting state fMRI scan, no behavioral measures were collected during the scan.

Acquisition

Imaging type(s): Functional

Field strength: 3 Tesla

Sequence & imaging parameters: T2*-weighted, echo planar sequence, 36 axial slices, repetition time = 2000 ms, echo time = 30 ms, flip angle = 90°, field of view = 192 × 192 mm2, voxel size = 3.0 × 3.0 × 3.0 mm3, interleaved collection

Area of acquisition: Whole brain scan

Diffusion MRI: ☐ Used ☒ Not used

Preprocessing

Preprocessing software: Resting state fMRI images were preprocessed using the FMRIB (Oxford Centre for Functional MRI of the Brain) Software Library (FSL) and Analysis of Functional NeuroImages software. The preprocessing steps were as follows: 1) removal of first five volumes for magnetic field stabilization; 2) motion and slice timing correction; 3) time series despiking; 4) grand mean scaling; 5) spatial smoothing with a 6 mm Gaussian kernel, 6) temporal band-pass frequency filtering in the range of 0.009 to 0.1 Hz; 7) removal of linear and quadratic trends; 8) co-registration of T1-weighted image using boundary based registration, followed by non-linear registration of fMRI image to Montreal Neurological Institute 152 (MNI152) space using FNIRT; and 9) regression of nine nuisance signals (global signal, white matter, cerebrospinal fluid and six motion parameters) from the preprocessed fMRI images.

Normalization: Co-registration of T1-weighted image using boundary based registration, followed by non-linear registration of fMRI image to Montreal Neurological Institute 152 (MNI152) space using FNIRT

Normalization template: Montreal Neurological Institute 152 (MNI152) template

Noise and artifact removal: Nine nuisance signals (global signal, white matter, cerebrospinal fluid and six motion parameters) were regressed from the preprocessed fMRI images. Prior to preprocessing, images that did not meet motion criteria were not included in the analyses (all images included in study had maximum relative displacement ≤ 1 mm and maximum absolute displacement ≤ 4). mm).

Volume censoring: Volume censoring was not performed in this study.

Statistical modeling & inference

Model type and settings: Associations of handgrip strength with cognitive performance (global cognition, processing speed, attention, executive function and SM-MMSE scores) and functional connectivity measures (global and network-level measures of system segregation, intra-/inter-network functional connectivity) were examined using separate linear regression models (mass univariate) with handgrip strength as predictor and cognitive or functional connectivity measure as outcome variable. Associations of functional connectivity measures implicated in handgrip strength with cognitive performance were also

examined using separate linear regression models with functional connectivity measure as predictor and cognitive performance as outcome variable. Finally, exploratory mediation effects of functional connectivity measures (implicated in both handgrip strength and cognitive performance) on the relationship between handgrip strength and cognitive performance were examined using separate mediation analyses with functional connectivity measure as mediator, handgrip strength as predictor and cognitive performance as outcome variable.

Effect(s) tested

Effect of handgrip strength on cognitive performance or functional connectivity measure was tested using linear regression models. Effect of various functional connectivity measures on cognitive performance was tested using linear regression models. Mediation effect of various functional connectivity measures on the association between handgrip strength and cognitive performance were performed using mediation models.

Specify type of analysis: ☐ Whole brain ☐ ROI-based ☒ Both

Anatomical location(s)

BOLD time series were first extracted from 114 cortical regions (Yeo et al., 2011) and 30 subcortical regions (Choi et al., 2012; Tzourio-Mazoyer et al., 2002) in MNI152 space. Two cortical regions were subsequently dropped due to poor brain coverage, resulting in a total of 142 regions.

Statistic type for inference

(See Eklund et al. 2016)

Region-wise or network-wise inference was performed

Correction

False discovery rate correction was performed to correct for multiple comparisons. For associations with handgrip strength, correction was performed either across 5 cognitive measures (when examining associations with cognition), 9 network-level system segregation measures (when examining associations with network-level system segregation), 9 salience/ventral attention inter- and intra-network functional connectivity measures (when examining associations with salience/ventral attention inter- and intra-network functional connectivity), or across 231 pairs of regional connections within the salience/ventral attention network (when examining associations with regional connections within salience/ventral attention network). For associations between functional connectivity and cognitive performance, correction was performed for each functional connectivity measure of interest across 5 cognitive measures.

Models & analysis

n/a	Involved in the study
☐	☒ Functional and/or effective connectivity
☐	☒ Graph analysis
☒	☐ Multivariate modeling or predictive analysis

Functional and/or effective connectivity

Functional connectivity matrices were obtained by computing Pearson correlation between the time courses of each pair of regions, and transforming the correlation values to z-scores using Fisher's r-to-z-transformation.

Graph analysis

We computed subject-level global and network-level measures of system segregation introduced by Chan et al., 2014, which quantifies differences in within-network connections relative to between-network connections. For computation of segregation measures, all diagonal elements and negative connections in the functional connectivity matrices were set to 0.
