# Supplementary material for: Higher handgrip strength is linked to higher salience ventral attention functional network segregation in older adults
Source: Commun Biol. 2024 Feb 21;7:214. doi: 10.1038/s42003-024-05862-x (PMC10881588; doi:10.1038/s42003-024-05862-x)
Supplement: Supplementary file 1 — Peer Review File [file 42003_2024_5862_MOESM1_ESM.pdf]

nature portfolio

Peer Review File

Higher handgrip strength is linked to higher salience ventral attention functional network segregation in older adults

Open Access This file is licensed under a Creative Commons Attribution 4.0 International License, which permits use, sharing, adaptation, distribution and reproduction in any medium or format, as long as you give appropriate credit to the original author(s) and the source, provide a link to the Creative Commons license, and indicate if changes were made. In the cases where the authors are anonymous, such as is the case for the reports of anonymous peer reviewers, author attribution should be to 'Anonymous Referee' followed by a clear attribution to the source work. The images or other third party material in this file are included in the article's Creative Commons license, unless indicated otherwise in a credit line to the material. If material is not included in the article's Creative Commons license and your intended use is not permitted by statutory regulation or exceeds the permitted use, you will need to obtain permission directly from the copyright holder. To view a copy of this license, visit http://creativecommons.org/licenses/by/4.0/.

Reviewers' comments:

Reviewer #1 (Remarks to the Author):

Higher handgrip strength is linked to higher salience/ventral attention functional network segregation in older adults

Summary: This study investigated the association between cognitive function and motor control and resting-state functional segregation in older adults. Resting-state functional segregation for each network was determined by a network segregation score, which takes into account intra- and inter-network connectivity. Consistent with previous studies, the authors found that hand-grip strength was associated with cognitive function. Higher hand-grip strength was also associated with greater segregation of the salience network; secondary analysis revealed that this relationship was primarily driven by the intra-network connectivity in the salience network. Greater salience network segregation was also associated with processing speed and global cognitive function.
While the paper is of interest and is well written, I have some concerns.

Throughout the paper, inter-individual variability is referred to as "differences." I think this may confuse readers as "differences" often imply between groups or between timepoints. Consider using "variability" or "inter-individual differences" instead.

INTRODUCTION:

Throughout the introduction, the authors reference the importance of looking at brain network segregation with physical frailty as a justification for the study. For example, pg 6 ln 118 "Nevertheless, little is known whether and how brain network segregation loss relates to physical frailty and cognition." I agree that this is important, but this does not accurately justify this study as motor performance/strength (i.e., hand grip strength, which is used in this study) is just one symptom for the physical frailty phenotype/syndrome. The introduction can benefit from elaboration of why hand-grip strength is important (vs. other motor performance measures), and real-life, clinical applications.

I found the justification for the novelty in the introduction and discussion section for the study underwhelming. The authors claim there is a lack of studies looking at motor performance and functional connectivity, which is true; however, there are studies that looked at network segregation (not only the sensorimotor network) with aging. In addition, as sensorimotor network segregation has already been demonstrated to be associated with sensorimotor performance, it is not clear from the introduction why this should be re-examined.
Some suggestion for references:
Cassady et al., 2020. Neuroimage. PMID: 32109601
Crowell et al., 2020. Neuroimage. PMID: 32442638
Grady et al., 2016. Neurobiology of Aging. PMID: 270103529

Moreover, the introduction criticizes region of interest / network approaches to functional connectivity, but here they only investigate sensorimotor and salience networks, matching limitations of previous studies. The introduction also needs further justification for selection of these two networks… For example, why not look at the dorsal attention network or the default mode network?

METHODS:

Related to the comment above for the introduction section, this study used community-dwelling adults to look for associations in older adults. It would be helpful to see if any other characteristics of physical frailty (e.g., walking speed, fatigue, level of physical activity) were collected, and if so to include it in the participant demographics table.

In addition, can authors provide the range for the age and main outcome variables (hand-grip strength and cognitive performance) in the participant tables? At least for the age, the SD seems to be small and this information will help to inform future studies.

For the cognition performance, it will be helpful for readers if the minimum and maximum possible score and how to interpret these scores.

I realize the authors refer to references for the ROIs used, but it will be helpful to have the size of the ROIs used in the main text and their locations illustrated in a figure. The locations could also be provided in a supplementary table or figure.

The methods state that measures of handgrip strength were collected within 18 months of the MRI scan. This is quite a long time. Please present data on this distribution. Was the time lag / lead used as a covariate in analyses?

Multiple tests were performed for each cognitive domain. It's not clear whether the authors created a composite score for each domain for the correlations with network segregation and if so, how.

Why was TICV included as a covariate for the functional connectivity analyses?

The results says that other networks were not associated with handgrip strength. Which other networks? The introduction and methods imply that only sensorimotor and salience networks were examined. Even though the analyses were FDR corrected, hundreds of correlations were performed. This seems rather excessive given the sample size.

RESULTS

Overall results were clear and well-presented. Can authors replace Figure 1A with the location of the nodes used for the salience network? This will help readers for the methods as well.

Although the correlations between network segregation and hand grip strength are significant, they are quite small in terms of percent variance accounted for. This should be discussed; is this really a meaningful relationship? Moreover, the results are largely driven by within network connectivity. This is not segregation per se but rather connectivity strength within a given network, which has been extensively examined.

The authors refer to both sensorimotor and somatomotor networks throughout the paper; are these meant to be the same network?

DISCUSSION

Pg 16, second paragraph is repetitive of the first paragraph, and re-summarizes the findings. It would be beneficial to expand on the cognition x motor association and its implications.

This study used several measures of cognitive performance, but it does not explain or discuss the outcomes and implications for the different measures used. This is touched upon very briefly in pg 17, but a clear paragraph on why global cognition and processing speed but not others were associated with functional connectivity, and implications for the findings will be helpful.

I believe one of the unique parts of this study is that the authors delved deeper into the segregation score to determine what part of the segregation score was associated to motor and cognitive function. Some of it is addressed on pg 17, but can the authors expand on why the associations were driven by intra-network connectivity? Again, this is not really network segregation in this case but just within network connectivity strength.

Similar to the comment for the introduction, in the last paragraph in pg 18, the authors indicate that they did not find associations with the sensorimotor network segregation due to the functional connectivity measure used. However, in Cassady et al., 2020 (Neuroimage, PMID 32109601), and Cassady et al., 2019 (Neuroimage, PMID: 30414983) sensorimotor network segregation is associated with sensorimotor performance that includes grip-strength. Can the authors expand on why sensorimotor network segregation score was not associated with motor (and cognitive) function?

Minor comments:

Pg 18, ln 418, please cite these studies

Reviewer #2 (Remarks to the Author):

This study aims to better understand the relationships among handgrip strength, brain functional connectivity and cognition in aging. The authors find that connectivity within a salience/ventral attention networks correlates with grip strength and cognition. The study is very clearly written. However, the results are largely descriptive and the paper only establishes bivariate cross-sectional relationships in line with other studies (they are acknowledged in the introduction). It is my opinion that the novel contribution of the study is better appreciated by a more specialised audience. Listed below are some other points to consider.

1. The authors establish associations of functional connectivity with cognition and grip strength but the three variables are never included in the same model to test, for example, whether the variance shared between functional connectivity and grip strength is also shared by cognition (whether one mediates the other). Additional analyses that put the 3 variables of interest in direct relationship with each other should be included to help interpretation and novel conclusions that can be drawn from the data.

2. The authors acknowledge that the cross-sectional nature of the study is a limitation. Are there any relevant variables in the study that have been collected longitudinally and could be incorporated to better understand sequences of events?

3. The time between grip strength assessment and MRI is reported to be within 18 months. Specify why the gap is so long, whether the results hold when time between measures is included as a covariate and whether one assessment systematically preceded the other.

4. Specify what frequencies were included in the bandpass filter.

5. Line 241 specify how many tests are performed.

6. Line 274, the control analyses should be foreshadowed in the introduction and methods. Their motivation and relevance is unclear.

Responses to reviewers:

We would like to thank the editor and both reviewers for their insightful comments and constructive suggestions. We have conducted new analyses and revised the manuscript to address the comments raised by the reviewers. We believe that the revised manuscript has been improved and the questions from the reviewers have been well addressed.

Detailed point-to-point responses to reviewers are as follows:

Reviewer #1:

1. Throughout the paper, inter-individual variability is referred to as "differences." I think this may confuse readers as "differences" often imply between groups or between timepoints. Consider using "variability" or "inter-individual differences" instead.

Response: We thank the reviewer for the comment. As suggested by the reviewer, we have amended our use of the term "differences" to "inter-individual differences" throughout the manuscript.

INTRODUCTION:

2. Throughout the introduction, the authors reference the importance of looking at brain network segregation with physical frailty as a justification for the study. For example, pg 6 ln 118 "Nevertheless, little is known whether and how brain network segregation loss relates to physical frailty and cognition." I agree that this is important, but this does not accurately justify this study as motor performance/strength (i.e., hand grip strength, which is used in this study) is just one symptom for the physical frailty phenotype/syndrome. The introduction can benefit from elaboration of why hand-grip strength is important (vs. other motor performance measures), and real-life, clinical applications.

Response: We thank the reviewer for this important comment. As recommended, we have revised the Introduction (pp. 4, lines 65-81) to specifically emphasize the importance of handgrip strength and its potential clinical applications, as follows (amendments are in bold):

"Handgrip strength, an indicator of skeletal muscle strength, is an important determinant of physical frailty and healthy ageing[1-3]. **While several measures of muscle strength and physical frailty exist, handgrip strength is of particular interest because of its low cost, ease of administration[4], good reliability[5] and strong predictive value for health and cognitive outcomes[6,7].** Lower handgrip strength has been widely associated with reduced quality of life[8,9], as well as disability

and mortality in older age[10-14]. Converging evidence also suggests that lower handgrip strength is linked to cognitive impairment and lower cognitive function in older adults[15-19]. A coordinated analysis of nine longitudinal ageing studies, for example, has demonstrated consistent association between greater decline in handgrip strength and greater decline in cognitive function[15]. **Further, strength and resistance training has been shown to improve cognition in older adults[20-22], underscoring the potential of such physical interventions for preventing and slowing cognitive impairment.** Given the close link between handgrip strength and cognition, it has been suggested that convergent age-related changes in brain function and structure may underlie this relationship between handgrip strength and cognitive function in older adults[23]. Understanding the neural mechanisms underlying the handgrip strength-cognition relationship is therefore of interest and could facilitate the development of new interventions aimed at mitigating age-related cognitive and physical decline."

Further, we have reduced emphasis on physical frailty, instead focusing on handgrip strength in the manuscript. The revised sections (amendments are in bold) in the Introduction (pp. 5-6, lines 92-96, 107-109, 125-128) are as follows:

"Studies have linked **other somatomotor measures (e.g., gait speed) and the physical frailty phenotype (e.g., Fried's frailty phenotype)** to inter-individual differences in functional connectivity of somatomotor regions or networks in older adults[24-26] as well, indicating that **handgrip strength and other somatomotor measures** might specifically implicate functional brain networks that are involved in motor function."

"One key limitation of these studies is the lack of a whole-brain perspective when examining **handgrip strength or other motor function-related** inter-individual differences in functional connectivity."

"Nevertheless, such studies have focused only on segregation of somatomotor networks, and little is known whether and how functional segregation loss of diverse networks across the whole brain relates to **handgrip strength and cognition**."

3. I found the justification for the novelty in the introduction and discussion section for the study underwhelming. The authors claim there is a lack of studies looking at motor performance and functional connectivity, which is true; however, there are studies that looked at network segregation (not only the sensorimotor network) with aging. In addition, as sensorimotor network segregation has already been demonstrated to be associated with sensorimotor performance, it is not clear from the introduction why this should be re-examined.
Some suggestion for references:
Cassady et al., 2020. Neuroimage. PMID: 32109601
Crowell et al., 2020. Neuroimage. PMID: 32442638

Grady et al., 2016. Neurobiology of Aging. PMID: 270103529

Response: We thank the reviewer for the useful comment and references. We would like to clarify that our study examined associations between handgrip strength and functional segregation of all 9 networks across the brain (default, control, limbic, salience/ventral attention, dorsal attention, somatomotor, visual, temporoparietal and subcortical regions). This comprehensive characterization has never been done before. Previous studies have only focused on one brain network or one network measure. To better reflect the novelty of our study in the manuscript, we have amended the Introduction (pp. 6, lines 117-132), Methods (pp. 11-12, lines 250-254) and Results (pp. 14, lines 315-319). Specifically, we included the studies by Crowell et al.[27] and Grady et al.[28] as references supporting age-related loss in functional segregation (Introduction: pp. 6, lines 117-120), as well as highlighted the previous studies by Cassady et al.[29,30] (Introduction: pp. 6, lines 123-125) demonstrating associations between somatomotor network segregation and somatomotor performance. The revised sections are provided below (amendments are in bold).

<u>Introduction (pp. 6, lines 117-132)</u>

"Convergent findings across studies utilizing different analytical approaches to quantify functional segregation have demonstrated age-related declines in system-wide segregation of functional brain networks, particularly that of the higher-order associative networks**[27,28,31-36].** Importantly, lower functional segregation has been associated with poorer cognitive performance in healthy older adults[31,34,37], suggesting that loss of segregation of functional networks could drive cognitive decline in ageing. **Further, higher segregation of somatomotor networks have been demonstrated to relate to better gross (including handgrip strength) and fine somatomotor performance, particularly in older adults[29,30]. Nevertheless, such studies have focused only on the segregation of somatomotor networks, and little is known whether and how functional segregation loss of diverse networks across the whole brain relates to handgrip strength and cognition.**

The aim of the study is therefore to examine the association between handgrip strength and **functional segregation of the various networks across the brain** in community-dwelling older adults, and how inter-individual differences in the functional segregation of handgrip strength-related networks may be linked to inter-individual differences in cognitive function."

<u>Methods (pp. 11-12, lines 250-254)</u>

"Associations of handgrip strength with cognitive performance (SM-MMSE scores as well as composite scores for global cognition, processing speed, attention and executive function) and system segregation **(global and nine network-level measures (pertaining to each of the nine brain networks))** were examined using linear regression models with handgrip strength as outcome variable and cognitive or segregation measure as predictor."

Results (pp. 14, lines 315-319)

"Next, we examined how handgrip strength was linked to **global and network-level** functional brain segregation in older adults. Higher handgrip strength was associated with higher segregation of the salience/ventral attention network (FDR-adjusted $p < 0.05$, corrected for multiple comparisons across the nine network-level segregation measures) (Fig. 1b), but not global segregation or segregation of **the other eight networks** (Table 3)."

4. Moreover, the introduction criticizes region of interest / network approaches to functional connectivity, but here they only investigate sensorimotor and salience networks, matching limitations of previous studies. The introduction also needs further justification for selection of these two networks… For example, why not look at the dorsal attention network or the default mode network?

Response: We thank the reviewer for the comment. As mentioned in our response to the previous comment (comment #3), we have examined the associations between handgrip strength and functional segregation of all 9 networks across the brain (including the whole cerebral cortex and subcortical regions). We did not only focus on somatomotor and salience/ventral attention networks. We have amended the introduction accordingly (Introduction: pp. 6 lines 117-132; amendments are described in our response to comment #3 above) to better reflect this.

METHODS:

5. Related to the comment above for the introduction section, this study used community-dwelling adults to look for associations in older adults. It would be helpful to see if any other characteristics of physical frailty (e.g., walking speed, fatigue, level of physical activity) were collected, and if so to include it in the participant demographics table.

Response: As suggested, we have now included other measures of physical frailty in the revised Table 1 (see below). These include percent weight change since previous follow-up (mean duration = 7.03 (SD = 0.82, range = 4.45 – 8.65) years), timing for the timed up-and-go (TUG) test, and a measure of self-report exhaustion, in which participant indicated yes or no to the question "Do you feel full of energy?".

Table 1. Participant demographic, cognitive and physical characteristics

Characteristic	Participants (n = 148)
Age in years, mean (SD, range)	72.8 (3.9, 62.2 – 82.4)
Sex, female/male	83/65
Years of education, mean (SD, range)	7.28 (3.97, 0 – 16)
SM-MMSE score, mean (SD, range)	26.4 (2.4, 17 – 30)

Waist-hip ratio, mean (SD, range)	0.917 (0.068, 0.743 – 1.080)
Body mass index, mean (SD, range) (n = 146)	23.0 (3.4, 16.4 – 36.9)
GDS score, mean (SD, range)	2.33 (2.46, 0 – 14)
Cognitive domain scores, mean (SD, range)	
Global	50.0 (6.1, 31.9 – 65.6)
Processing speed	50.3 (8.3, 27.8 – 81.0)
Attention	49.6 (6.8, 25.9 – 71.7)
Executive function	50.1 (6.5, 38.3 – 68.0)
Physical frailty measures	
Handgrip strength (kg), mean (SD, range)	22.3 (7.0, 8.3 – 37.7)
Weight change (%)[1], mean (SD, range)	-0.29 (7.38, -30.77 – 25.69)
Timed Up-And-Go time (s), mean (SD, range)	10.3 (4.0, 6 – 43)
Exhaustion[2], yes/no	25/123

[1]Weight change (%) measures the percent weight change since previous follow-up (mean duration = 7.03 (SD = 0.82, range = 4.45 – 8.65) years). [2]For the exhaustion measure, participants were asked to indicate yes or no to the question "Do you feel full of energy?". Abbreviations: SD, standard deviation; SM-MMSE, Singapore-modified version of the mini-mental state examination; GDS, geriatric depression scale.

The Methods (pp. 8, lines 173-175) have also correspondingly been revised as follows:

"**Physical frailty measurements, including handgrip strength**, were collected within 18 months of the neuroimaging and neuropsychological assessments **(detailed protocol is described in previous work[10,38]).**"

6. In addition, can authors provide the range for the age and main outcome variables (hand-grip strength and cognitive performance) in the participant tables? At least for the age, the SD seems to be small and this information will help to inform future studies.

Response: As suggested by the reviewer, we have included the range for all continuous variables (including cognitive performance and handgrip strength) in Table 1 (see our response to comment #5 above).

7. For the cognition performance, it will be helpful for readers if the minimum and maximum possible score and how to interpret these scores.

Response: We thank the reviewer for the suggestion. To clarify, the cognitive scores for the three domains (processing speed, attention, executive function) are composite scores obtained by first converting z-transformed (using mean and standard deviation across all participants' scores) individual test scores to T-scores (with a mean of 50 and standard deviation of 10), then averaging T-scores across all tests within each domain. The global cognition score is taken as the average of these three composite scores. As these are T-scores, there are no bound for these scores, and the scores can go from $-\infty$ to ∞. If a participant had a T-score of 60 on a cognitive test, then their

score is higher than approximately 84% of the participants in the study (equivalent to one standard deviation above the mean score across all participants).

We have amended our Methods (pp. 8, lines 162-171) to provide some clarity on the interpretation of these cognitive scores. In particular, we have amended the Methods section as follows:

“For each test, individual scores were first z-transformed (using mean and standard deviation across all participants’ scores) and then subsequently converted to T-scores with a mean of 50 and standard deviation of 10. A participant who achieves a T-score of 60 on a cognitive test thus scored higher than approximately 84% of the participants in the study (equivalent to one standard deviation above the mean score across all participants). Composite scores for each of the three domains were then obtained by averaging T-scores across all tests within each domain. Additionally, global cognition scores were obtained for each participant by averaging across the composite scores of the three domains.”

8. I realize the authors refer to references for the ROIs used, but it will be helpful to have the size of the ROIs used in the main text and their locations illustrated in a figure. The locations could also be provided in a supplementary table or figure.

Response: We thank the reviewer for the comment. As a 2D figure is not sufficient to display all ROIs used in the study, we have instead shared the 3D nifti image on our github repository (https://github.com/hzlab/2023_Chong_CommunBiol_Gripstrength_FC/blob/main/Parcellation_142ROIs.nii; link is provided as a footnote in Supplementary Table 1). Additionally, we have provided the ROIs for the salience/ventral attention network in Figure 1 (please see our response to comment #13).

Further, we have included a new supplementary table listing the location and size (in mm) of all brain ROIs (Supplementary Table 1). The supplementary table is provided below.

Supplementary Table 1. Regions-of-interest used to compute functional connectivity measures

S/N	Network	Region	MNI coordinates			Size (mm)
			x (mm)	y (mm)	z (mm)	
1	Default	LH inferior parietal lobule	-48	-76	34	6160
2		LH dorsal prefrontal cortex	-18	28	42	4136
3		LH precuneus posterior cingulate cortex	-2	-48	18	8728
4		LH medial prefrontal cortex	-2	46	-4	11160
5		LH temporal cortex	-56	-38	-6	18280
6		LH inferior parietal lobule	-58	-52	26	4368
7		LH dorsal prefrontal cortex	-8	22	56	12640
8		LH lateral prefrontal cortex	-42	12	52	2272

9		LH ventral prefrontal cortex	-44	36	-12	11784
10		LH inferior parietal lobule	-44	-72	24	320
11		LH retrosplenial cortex	-12	-60	12	1384
12		LH parahippocampal cortex	-22	-38	-14	3760
13		RH temporal cortex	62	-8	-16	2024
14		RH inferior parietal lobule	58	-64	30	5568
15		RH dorsal prefrontal cortex	28	38	42	3368
16		RH precuneus posterior cingulate cortex	8	-48	20	5688
17		RH medial prefrontal cortex	4	52	-6	13760
18		RH temporal cortex	64	-34	-2	1976
19		RH anterior temporal cortex	52	8	-40	4264
20		RH dorsal prefrontal cortex	8	22	62	6616
21		RH ventral prefrontal cortex	52	30	-12	4216
22		RH inferior parietal lobule	56	-68	20	960
23		RH retrosplenial cortex	14	-56	12	1480
24		RH parahippocampal cortex	24	-32	-22	2352
25	Control	LH temporal cortex	-62	-62	-8	2584
26		LH intraparietal sulcus	-42	-44	44	6336
27		LH dorsal prefrontal cortex	-26	4	54	896
28		LH lateral prefrontal cortex	-34	2	32	6432
29		LH lateral ventral prefrontal cortex	-40	30	14	3120
30		LH mid-cingulate cortex	-2	6	28	176
31		LH temporal cortex	-62	-44	-16	1896
32		LH inferior parietal lobule	-52	-52	54	2936
33		LH dorsal prefrontal cortex	-30	12	58	344
34		LH lateral prefrontal cortex	-40	26	38	2184
35		LH lateral ventral prefrontal cortex	-36	56	2	5336
36		LH medial posterior prefrontal cortex	-4	28	42	464
37		LH precuneus	-4	-76	36	4560
38		LH posterior cingulate cortex	-4	-36	24	792
39		RH temporal cortex	56	-44	-12	1112
40		RH intraparietal sulcus	40	-46	38	4968
41		RH dorsal prefrontal cortex	30	6	54	480
42		RH lateral prefrontal cortex	48	34	12	9928
43		RH mid-cingulate cortex	8	4	28	184
44		RH temporal cortex	62	-32	-16	3392
45		RH inferior parietal lobule	48	-52	54	5736
46		RH lateral dorsal prefrontal cortex	40	22	34	10320
47		RH lateral ventral prefrontal cortex	36	56	-6	10152
48		RH medial posterior prefrontal cortex	6	38	40	1304
49		RH precuneus	12	-76	40	5304
50		RH posterior cingulate cortex	8	-36	24	1040
51	Limbic	LH temporal pole	-26	-8	-38	20392
52		LH orbital frontal cortex	-8	38	-24	15640
53		RH temporal pole	38	-14	-42	21064
54		RH orbital frontal cortex	8	36	-24	14160
55	Salience/ventral	LH parietal operculum	-58	-34	20	4960
56	attention	LH posterior insula/frontal operculum	-40	4	10	2816
57		LH posterior insula	-36	2	10	4384
58		LH midcingulate/medial parietal cortex	-8	-20	36	2248
59		LH medial frontal cortex	-6	8	32	4872
60		LH inferior parietal lobule	-64	-46	36	688

61		LH lateral prefrontal cortex	-32	54	20	8944
62		LH anterior insula	-38	16	-8	2736
63		LH orbital frontal cortex	-30	40	-18	328
64		LH medial posterior prefrontal cortex	-12	24	26	3528
65		RH parietal operculum	58	-32	28	5288
66		RH precentral gyrus	52	-2	50	504
67		RH posterior insula/frontal operculum	60	8	2	2880
68		RH posterior insula	38	6	6	4432
69		RH midcingulate/medial parietal cortex	10	-22	36	2280
70		RH medial frontal cortex	8	6	32	6112
71		RH inferior parietal lobule	60	-36	32	1448
72		RH lateral prefrontal cortex	30	44	16	8104
73		RH lateral ventral prefrontal cortex	52	38	-2	904
74		RH anterior insula	46	18	0	4552
75		RH medial posterior prefrontal cortex	6	22	40	3128
76		RH anterior cingulate sulcus	2	18	22	200
77	Dorsal attention	LH temporal occipital cortex	-50	-68	-8	5560
78		LH parietal occipital cortex	-28	-72	28	2768
79		LH superior parietal lobule	-18	-68	42	8656
80		LH temporal occipital cortex	-56	-60	6	560
81		LH post central gyrus	-54	-24	40	10576
82		LH frontal eye fields	-26	-14	52	5056
83		LH precentral ventral gyrus	-54	4	36	312
84		RH temporal occipital cortex	58	-64	-12	6232
85		RH parietal occipital cortex	42	-84	24	4584
86		RH superior parietal lobule	22	-66	42	12008
87		RH temporal occipital cortex	62	-60	2	416
88		RH post central gyrus	56	-24	36	10312
89		RH frontal eye fields	20	-12	60	3744
90		RH precentral ventral gyrus	60	8	26	664
91	Somatomotor	LH primary somatosensory cortex	-14	-38	74	32824
92		LH central sulcus	-62	0	20	6184
93		LH secondary somatosensory cortex	-60	-6	6	6512
94		LH posterior insula	-32	-24	4	216
95		LH auditory cortex	-54	-10	4	9232
96		RH primary somatosensory cortex	4	-38	62	28880
97		RH central sulcus	64	2	18	5520
98		RH secondary somatosensory cortex	62	-6	10	5184
99		RH posterior insula	36	-24	2	280
100		RH auditory cortex	56	-8	0	8656
101	Visual	LH striate cortex	-20	-104	-12	2944
102		LH extrastriate cortex	-32	-88	-4	31792
103		LH striate calcarine cortex	-16	-64	0	5112
104		LH inferior extrastriate cortex	-8	-56	-4	5640
105		LH superior extrastriate cortex	-18	-66	8	9288
106		RH striate cortex	14	-104	-4	2320
107		RH extrastriate cortex	38	-90	0	31920
108		RH striate calcarine cortex	18	-60	0	4736
109		RH inferior extrastriate cortex	20	-50	-4	7304
110		RH superior extrastriate cortex	16	-62	10	9568
111	Temporoparietal	LH temporoparietal junction	-58	-48	12	8904
112		RH temporoparietal junction	58	-36	4	12560

113	Subcortical	LH hippocampus	-28	-14	-18	7208
114		RH hippocampus	24	-14	-18	7272
115		LH amygdala	-22	-4	-16	1936
116		RH amygdala	22	-4	-14	2024
117		LH striatum	-30	-10	0	1528
118		LH striatum	-24	8	4	2096
119		LH striatum	-12	10	-10	1080
120		LH striatum	-24	6	-6	2896
121		LH striatum	-12	18	-2	3128
122		RH striatum	28	-12	-8	8
123		RH striatum	30	-6	-6	1256
124		RH striatum	32	-14	-6	88
125		RH striatum	24	10	0	2424
126		RH striatum	12	10	-10	992
127		RH striatum	14	8	14	3752
128		RH striatum	12	18	-2	1816
129		LH thalamus	-20	-30	4	1768
130		LH thalamus	-14	-22	4	1384
131		LH thalamus	-16	-28	12	272
132		LH thalamus	-6	-16	-2	3464
133		LH thalamus	-6	-10	-8	832
134		LH thalamus	-10	-6	8	1808
135		LH thalamus	-4	-8	8	2480
136		RH thalamus	18	-30	2	1952
137		RH thalamus	14	-22	2	1136
138		RH thalamus	18	-26	12	336
139		RH thalamus	6	-16	2	3960
140		RH thalamus	6	-10	-8	736
141		RH thalamus	8	-6	10	2544
142		RH thalamus	10	-30	6	1400

The brain map (nifti file) containing all regions-of-interest is provided here: https://github.com/hzlab/2023_Chong_CommunBiol_Gripstrength_FC/blob/main/Parcellation_142ROIs.nii. Abbreviations: LH, left hemisphere; RH, right hemisphere.

9. The methods state that measures of handgrip strength were collected within 18 months of the MRI scan. This is quite a long time. Please present data on this distribution. Was the time lag / lead used as a covariate in analyses?

Response: We thank the reviewer for this insightful comment. The distribution of the time interval between the MRI scan and handgrip strength assessment (i.e., scan date – handgrip strength assessment date) is given below. On average, the handgrip strength measurements were taken 276.56 (SD = 156.64, range = -20 – 547) days after the MRI scans.

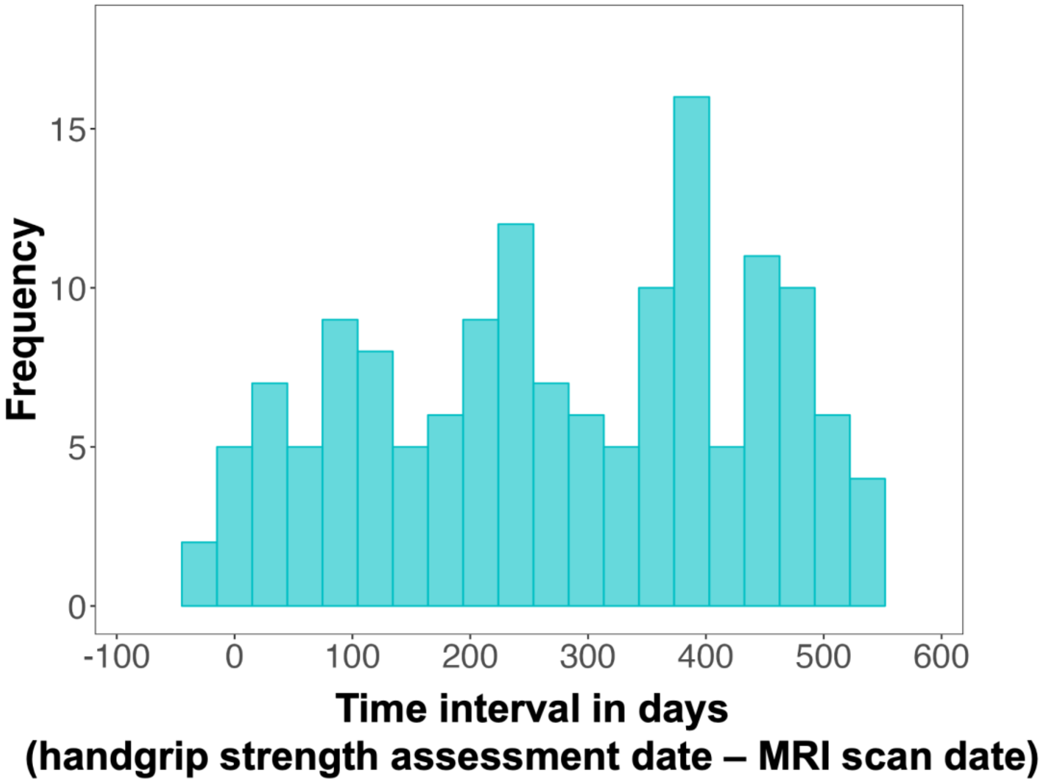

Supplementary Figure 1. Time interval between date of MRI scan and date of handgrip strength assessments. Histogram displays the time interval (in days) between the MRI scan and handgrip strength assessment (handgrip strength assessment date – MRI scan date). On average, handgrip strength assessments were performed 276.56 (SD = 156.64, range = -20 – 547) days after the MRI scans.

We have also repeated our analyses (i.e., associations between handgrip strength and functional connectivity measures) with time difference between MRI scan and handgrip strength assessment included as an additional covariate. Our results remained, suggesting little influence of the time interval between MRI scan and handgrip strength assessment on our key findings. These results (both the histogram and validation analysis controlling for the time interval) have been included in the following sections: Methods: pp. 13-14, lines 295-300, Results: pp. 15, lines 343-345, Supplementary Figure 1 (see above figure), and Supplementary Tables 4-6. The revised sections and supplementary tables are also provided below (amendments are in bold):

Methods (pp. 13-14, lines 295-300)

"**Lastly, handgrip strength assessments were found to be conducted on average 276.56 (SD = 156.64, range = -20 – 547) days after the MRI scans (Supplementary Fig. 1). Given this time difference between the MRI scan and handgrip assessment, we repeated the handgrip strength-functional connectivity association analyses with time interval (days) between the MRI scan and handgrip strength assessment included as an additional covariate.**"

Results (pp. 15, lines 343-345)

"All these findings remained after controlling for SM-MMSE scores, Geriatric Depression Scale scores, body mass index, waist-hip ratio **or time interval between MRI scan and handgrip strength assessment** (Supplementary Tables 4-6)."

Supplementary Table 4: Validation analyses: associations between handgrip strength and global and network-level system segregation

Measure	Coefficient	SE	*t*	Uncorr *p*	FDR-adj *p*
**Analysis 1: Analysis additionally controlling for SM-MMSE (n = 148)**					
Global segregation	$1.80e^{-3}$	$8.86e^{-4}$	2.03	0.0439*	0.0439*
Network segregation					
Default	$2.41e^{-3}$	$1.53e^{-3}$	1.57	0.1187	0.2916
Control	$6.78e^{-4}$	$1.65e^{-3}$	0.41	0.6815	0.7667
Limbic	$1.25e^{-3}$	$1.83e^{-3}$	0.68	0.4971	0.6541
Salience/ventral attention	$4.89e^{-3}$	$1.49e^{-3}$	3.29	0.0013*	0.0115*
Dorsal attention	$1.27e^{-3}$	$1.92e^{-3}$	0.66	0.5088	0.6541
Somatomotor	$2.89e^{-3}$	$1.70e^{-3}$	1.70	0.0909	0.2916
Visual	$2.89e^{-4}$	$1.09e^{-3}$	0.27	0.7905	0.7905
Temporoparietal	$5.26e^{-3}$	$3.45e^{-3}$	1.52	0.1296	0.2916
Subcortical	$1.25e^{-3}$	$1.24e^{-3}$	1.01	0.3137	0.5646
**Analysis 2: Analysis additionally controlling for waist-hip ratio (n = 148)**					
Global segregation	$1.68e^{-3}$	$8.90e^{-4}$	1.89	0.0607	0.0607
Network segregation					
Default	$2.34e^{-3}$	$1.54e^{-3}$	1.52	0.1298	0.2920
Control	$8.55e^{-4}$	$1.65e^{-3}$	0.52	0.6041	0.6796
Limbic	$1.22e^{-3}$	$1.83e^{-3}$	0.67	0.5056	0.6796
Salience/ventral attention	$5.01e^{-3}$	$1.48e^{-3}$	3.37	0.0010*	0.0086*
Dorsal attention	$1.11e^{-3}$	$1.93e^{-3}$	0.57	0.5665	0.6796
Somatomotor	$2.84e^{-3}$	$1.70e^{-3}$	1.68	0.0960	0.2920
Visual	$1.44e^{-4}$	$1.08e^{-3}$	0.13	0.8946	0.8946
Temporoparietal	$5.46e^{-3}$	$3.44e^{-3}$	1.59	0.1145	0.2920
Subcortical	$9.79e^{-4}$	$1.24e^{-3}$	0.79	0.4319	0.6796
**Analysis 3: Analysis additionally controlling for body mass index (n = 146)**					
Global segregation	$1.68e^{-3}$	$9.45e^{-4}$	1.78	0.0774	0.0774
Network segregation					
Default	$2.00e^{-3}$	$1.64e^{-3}$	1.22	0.2254	0.4550
Control	$7.16e^{-4}$	$1.73e^{-3}$	0.41	0.6803	0.7654
Limbic	$1.26e^{-3}$	$1.95e^{-3}$	0.65	0.5183	0.6664
Salience/ventral attention	$4.88e^{-3}$	$1.56e^{-3}$	3.12	0.0022*	0.0199*
Dorsal attention	$2.31e^{-3}$	$2.01e^{-3}$	1.15	0.2528	0.4550
Somatomotor	$2.77e^{-3}$	$1.80e^{-3}$	1.54	0.1262	0.3787
Visual	$2.84e^{-4}$	$1.15e^{-3}$	0.25	0.8062	0.8062
Temporoparietal	$6.37e^{-3}$	$3.48e^{-3}$	1.83	0.0693	0.3121
Subcortical	$1.13e^{-3}$	$1.32e^{-3}$	0.86	0.3937	0.5906
**Analysis 4: Analysis additionally controlling for GDS scores (n = 148)**					

Global segregation	$1.66e^{-3}$	$8.80e^{-4}$	1.89	0.0613	0.0613
Network segregation					
Default	$2.10e^{-3}$	$1.52e^{-3}$	1.38	0.1704	0.3835
Control	$6.93e^{-4}$	$1.64e^{-3}$	0.42	0.6730	0.7571
Limbic	$1.43e^{-3}$	$1.82e^{-3}$	0.78	0.4343	0.6515
Salience/ventral attention	$4.87e^{-3}$	$1.48e^{-3}$	3.30	0.0012*	0.0112*
Dorsal attention	$1.04e^{-3}$	$1.92e^{-3}$	0.54	0.5897	0.7571
Somatomotor	$2.83e^{-3}$	$1.68e^{-3}$	1.69	0.0939	0.3726
Visual	$2.21e^{-4}$	$1.07e^{-3}$	0.21	0.8372	0.8372
Temporoparietal	$5.22e^{-3}$	$3.38e^{-3}$	1.55	0.1242	0.3726
Subcortical	$1.04e^{-3}$	$1.23e^{-3}$	0.84	0.4007	0.6515
**Analysis 5: Analysis additionally controlling for time interval between MRI scan and handgrip strength measurement (n = 148)**					
**Global segregation**	**$1.52e^{-3}$**	**$8.99e^{-4}$**	**1.69**	**0.0934**	**0.0934**
**Network segregation**					
**Default**	**$2.49e^{-3}$**	**$1.56e^{-3}$**	**1.60**	**0.1127**	**0.3203**
**Control**	**$-4.20e^{-5}$**	**$1.64e^{-3}$**	**-0.03**	**0.9796**	**0.9796**
**Limbic**	**$1.36e^{-3}$**	**$1.86e^{-3}$**	**0.73**	**0.4675**	**0.7344**
**Salience/ventral attention**	**$4.33e^{-3}$**	**$1.49e^{-3}$**	**2.91**	**0.0042***	**0.0378***
**Dorsal attention**	**$1.10e^{-3}$**	**$1.96e^{-3}$**	**0.56**	**0.5761**	**0.7406**
**Somatomotor**	**$3.31e^{-3}$**	**$1.71e^{-3}$**	**1.94**	**0.0549**	**0.2472**
**Visual**	**$-4.08e^{-4}$**	**$1.06e^{-3}$**	**-0.38**	**0.7015**	**0.7892**
**Temporoparietal**	**$5.16e^{-3}$**	**$3.50e^{-3}$**	**1.48**	**0.1424**	**0.3203**
**Subcortical**	**$8.72e^{-4}$**	**$1.26e^{-3}$**	**0.69**	**0.4896**	**0.7344**

Multiple comparison correction was performed across the nine network-level segregation measures. * indicates statistically significant effects ($p < 0.05$). Abbreviations: SE, standard error; Uncorr, uncorrected; FDR-adj, false discovery rate-adjusted; SM-MMSE, Singapore-modified version of the mini-mental state examination; GDS, geriatric depression scale scores.

Supplementary Table 5: Validation analyses: associations between handgrip strength and mean salience/ventral attention inter- and intra-network functional connectivity

**Measure**	**Coefficient**	**SE**	***t***	**Uncorr *p***	**FDR-adj *p***
**Analysis 1: Analysis additionally controlling for SM-MMSE (n = 148)**					
Intra-network S/VA	$4.21e^{-3}$	$1.47e^{-3}$	2.87	0.0048*	0.0428*
Inter-network					
S/VA – Default	$-3.04e^{-3}$	$1.40e^{-3}$	-2.18	0.0312*	0.0935
S/VA – Control	$-9.52e^{-4}$	$1.07e^{-3}$	-0.89	0.3740	0.4927
S/VA – Limbic	$-8.40e^{-4}$	$1.45e^{-3}$	-0.58	0.5636	0.5636
S/VA – Dorsal attention	$1.20e^{-3}$	$1.37e^{-3}$	0.87	0.3832	0.4927
S/VA – Somatomotor	$3.34e^{-3}$	$1.44e^{-3}$	2.32	0.0215*	0.0935
S/VA – Visual	$2.72e^{-3}$	$1.81e^{-3}$	1.50	0.1347	0.3031
S/VA – Temporoparietal	$1.32e^{-3}$	$1.94e^{-3}$	0.68	0.4991	0.5615
S/VA – Subcortical	$9.88e^{-4}$	$9.31e^{-4}$	1.06	0.2902	0.4927
**Analysis 2: Analysis additionally controlling for waist-hip ratio (n = 148)**					
Intra-network S/VA	$4.47e^{-3}$	$1.46e^{-3}$	3.05	0.0027*	0.0243*
Inter-network					
S/VA – Default	$-3.14e^{-3}$	$1.39e^{-3}$	-2.26	0.0253*	0.0758
S/VA – Control	$-9.73e^{-4}$	$1.07e^{-3}$	-0.91	0.3632	0.5067

S/VA – Limbic	$-8.59e^{-4}$	$1.45e^{-3}$	-0.59	0.5550	0.5550
S/VA – Dorsal attention	$1.57e^{-3}$	$1.33e^{-3}$	1.18	0.2414	0.4345
S/VA – Somatomotor	$3.45e^{-3}$	$1.44e^{-3}$	2.40	0.0176*	0.0758
S/VA – Visual	$2.66e^{-3}$	$1.81e^{-3}$	1.46	0.1452	0.3266
S/VA – Temporoparietal	$1.47e^{-3}$	$1.94e^{-3}$	0.76	0.4504	0.5067
S/VA – Subcortical	$7.69e^{-4}$	$9.23e^{-4}$	0.83	0.4062	0.5067
**Analysis 3: Analysis additionally controlling for body mass index (n = 146)**					
Intra-network S/VA	$4.37e^{-3}$	$1.56e^{-3}$	2.81	0.0057*	0.0510
Inter-network					
S/VA – Default	$-2.48e^{-3}$	$1.47e^{-3}$	-1.68	0.0946	0.2839
S/VA – Control	$-3.66e^{-4}$	$1.13e^{-3}$	-0.32	0.7475	0.7475
S/VA – Limbic	$-6.59e^{-4}$	$1.54e^{-3}$	-0.43	0.6683	0.7475
S/VA – Dorsal attention	$1.20e^{-3}$	$1.42e^{-3}$	0.84	0.4018	0.6925
S/VA – Somatomotor	$3.06e^{-3}$	$1.52e^{-3}$	2.01	0.0461*	0.2075
S/VA – Visual	$2.07e^{-3}$	$1.93e^{-3}$	1.07	0.2845	0.6401
S/VA – Temporoparietal	$1.15e^{-3}$	$2.04e^{-3}$	0.56	0.5743	0.7384
S/VA – Subcortical	$7.18e^{-4}$	$9.73e^{-4}$	0.74	0.4617	0.6925
**Analysis 4: Analysis additionally controlling for GDS scores (n = 148)**					
Intra-network S/VA	$4.30e^{-3}$	$1.46e^{-3}$	2.94	0.0038*	0.0345*
Inter-network					
S/VA – Default	$-2.74e^{-3}$	$1.39e^{-3}$	-1.98	0.0499*	0.1496
S/VA – Control	$-7.28e^{-4}$	$1.07e^{-3}$	-0.68	0.4961	0.5425
S/VA – Limbic	$-8.81e^{-4}$	$1.44e^{-3}$	-0.61	0.5425	0.5425
S/VA – Dorsal attention	$1.08e^{-3}$	$1.36e^{-3}$	0.80	0.4261	0.5425
S/VA – Somatomotor	$3.32e^{-3}$	$1.43e^{-3}$	2.33	0.0214*	0.0963
S/VA – Visual	$2.38e^{-3}$	$1.81e^{-3}$	1.31	0.1910	0.4298
S/VA – Temporoparietal	$1.33e^{-3}$	$1.91e^{-3}$	0.70	0.4879	0.5425
S/VA – Subcortical	$9.51e^{-4}$	$9.26e^{-4}$	1.03	0.3061	0.5425
**Analysis 5: Analysis additionally controlling for time interval between MRI scan and handgrip strength measurement (n = 148)**					
**Intra-network S/VA**	**$3.97e^{-3}$**	**$1.48e^{-3}$**	**2.67**	**0.0084***	**0.0753**
**Inter-network**					
**S/VA – Default**	**$-2.65e^{-3}$**	**$1.43e^{-3}$**	**-1.85**	**0.0658**	**0.1975**
**S/VA – Control**	**$-7.25e^{-4}$**	**$1.09e^{-3}$**	**-0.66**	**0.5081**	**0.5716**
**S/VA – Limbic**	**$-1.21e^{-3}$**	**$1.47e^{-3}$**	**-0.82**	**0.4117**	**0.5294**
**S/VA – Dorsal attention**	**$1.19e^{-3}$**	**$1.39e^{-3}$**	**0.85**	**0.3943**	**0.5294**
**S/VA – Somatomotor**	**$3.05e^{-3}$**	**$1.45e^{-3}$**	**2.10**	**0.0372***	**0.1674**
**S/VA – Visual**	**$2.59e^{-3}$**	**$1.85e^{-3}$**	**1.40**	**0.1623**	**0.3653**
**S/VA – Temporoparietal**	**$7.60e^{-4}$**	**$1.96e^{-3}$**	**0.39**	**0.6986**	**0.6986**
**S/VA – Subcortical**	**$1.11e^{-3}$**	**$9.42e^{-4}$**	**1.18**	**0.2399**	**0.4319**

Multiple comparison correction was performed across the nine salience/ventral attention inter- and intra-network functional connectivity measures. * indicates statistically significant effects (p < 0.05). Abbreviations: SE, standard error; Uncorr, uncorrected; FDR-adj, false discovery rate-adjusted; S/VA, salience/ventral attention; SM-MMSE, Singapore-modified version of the mini-mental state examination; GDS, geriatric depression scale scores.

Supplementary Table 6: Validation analyses: significant associations between handgrip strength and intra-network salience/ventral attention functional connections

Measure	Coefficient	SE	*t*	Uncorr *p*	FDR-adj *p*
**Analysis 1: Analysis additionally controlling for SM-MMSE (n = 148)**					
Right AI – Left PI/FO	$1.54e^{-2}$	$3.86e^{-3}$	3.99	0.0001*	0.0177*
Right AI – Right MCC/MPC	$1.53e^{-2}$	$3.92e^{-3}$	3.89	0.0002*	0.0177*
**Analysis 2: Analysis additionally controlling for waist-hip ratio (n = 148)**					
Right AI – Left PI/FO	$1.62e^{-2}$	$3.81e^{-3}$	4.24	<0.0001*	0.0046*
Right AI – Right MCC/MPC	$1.66e^{-2}$	$3.87e^{-3}$	4.30	<0.0001*	0.0046*
**Analysis 3: Analysis additionally controlling for body mass index (n = 146)**					
Right AI – Left PI/FO	$1.57e^{-2}$	$4.09e^{-3}$	3.84	0.0002*	0.0191*
Right AI – Right MCC/MPC	$1.58e^{-2}$	$4.11e^{-3}$	3.84	0.0002*	0.0191*
**Analysis 4: Analysis additionally controlling for GDS scores (n = 148)**					
Right AI – Left PI/FO	$1.53e^{-2}$	$3.83e^{-3}$	4.00	0.0001*	0.0126*
Right AI – Right MCC/MPC	$1.55e^{-2}$	$3.90e^{-3}$	3.98	0.0001*	0.0126*
**Analysis 5: Analysis additionally controlling for time interval between MRI scan and handgrip strength measurement (n = 148)**					
**Right AI – Left PI/FO**	**$1.44e^{-2}$**	**$3.89e^{-3}$**	**3.70**	**0.0003***	**0.0358***
**Right AI – Right MCC/MPC**	**$1.51e^{-2}$**	**$3.98e^{-3}$**	**3.79**	**0.0002***	**0.0358***

Multiple comparison correction was performed across 231 pairs of regional connections within the salience/ventral attention network. * indicates statistically significant effects ($p < 0.05$). Abbreviations: SE, standard error; Uncorr, uncorrected; FDR-adj, false discovery rate-adjusted; SM-MMSE, Singapore-modified version of the mini-mental state examination; GDS, geriatric depression scale scores; AI, anterior insula; PI, posterior insula; FO, frontal operculum; MCC, midcingulate cortex; MPC, medial parietal cortex.

10. Multiple tests were performed for each cognitive domain. It's not clear whether the authors created a composite score for each domain for the correlations with network segregation and if so, how.

Response: We thank the reviewer for the comment. All associations with cognition were performed using composite scores for each cognitive domain. We have provided further clarification for this in the Methods (pp. 8 lines 162-171, pp. 11 lines 250-251, pp. 13 lines 280-282). Specifically, we have amended our description to describe how these composite scores were calculated (Methods: pp. 8 lines 162-171, also see our earlier response to comment #7), as well as state that measures of cognitive performance included "SM-MMSE scores as well as composite scores for global cognition, processing speed, attention and executive function" when examining both associations between handgrip strength and cognitive performance and associations between functional connectivity measures and cognitive performance (Methods: pp. 11 lines 250-251, pp. 13 lines 280-282).

11. Why was TICV included as a covariate for the functional connectivity analyses?

Response: We have included total intracranial volumes (TIV) as a covariate to account for the influence of brain sizes in the analyses. TIV has been demonstrated to decline with age in older adults[39], and previous functional connectivity studies in older adults have also controlled for TIV in their analyses[40-44]. We have included a short description ("to account for total brain sizes") in the Methods (pp. 12 lines 260-261, pp. 13 line 285) to explain why we controlled for TIV in our analyses.

Additionally, given that the associations between handgrip strength and cognitive measures do not involve brain measures, we have revised Table 2 to reflect the associations between handgrip strength and cognitive performance after controlling for age, sex and education (but not TIV). The handgrip strength-cognition associations were similar with or without controlling for TIV. The revised table and corresponding revised description in the Methods (pp. 12, lines 258-261) is provided below:

Table 2: Associations between handgrip strength and cognitive measures

Cognitive measure	Coefficient	SE	*t*	Uncorr *p*	FDR-adj *p*
Global	0.251	0.076	3.30	0.0012*	0.0059*
Processing speed	0.327	0.106	3.09	0.0024*	0.0059*
Attention	0.237	0.098	2.41	0.0172*	0.0287*
Executive function	0.187	0.091	2.06	0.0412	0.0516
SM-MMSE	0.043	0.035	1.23	0.2196	0.2196

All models controlled for age, sex and education. Multiple comparison correction was performed across the five cognitive measures. * indicates statistically significant effects ($p < 0.05$). Abbreviations: SE, standard error; Uncorr, uncorrected; FDR-adj, false discovery rate-adjusted; SM-MMSE, Singapore-modified version of the mini-mental state examination.

Methods (pp. 12, lines 258-261)

"For models examining handgrip strength-cognition associations, age, sex and education were included as nuisance covariates, while for models examining functional connectivity-cognition associations, age, sex, education, and total intracranial volumes (to account for total brain sizes) were included as nuisance covariates."

12. The results says that other networks were not associated with handgrip strength. Which other networks? The introduction and methods imply that only sensorimotor and salience networks were examined. Even though the analyses were FDR corrected, hundreds of correlations were performed. This seems rather excessive given the sample size.

Response: We thank the reviewer for the comment. As clarified in our response to the earlier comments (comments #3 and #4), we have examined associations

between handgrip strength and functional segregation of each of nine brain networks (default, control, limbic, salience/ventral attention, dorsal attention, somatomotor, visual, temporoparietal and subcortical). Relevant sections in the Introduction (pp. 6, lines 117-132), Methods (pp. 11-12, lines 250-254) and Results (pp. 14, lines 315-319) have been revised to address this (provided in our response to comments #3).

In addition, we have revised the Results and Tables (Results: pp. 14 lines 305-306, 317-318, pp. 15 lines 325-327, 341-342, pp. 16 lines 363, 368-369, Tables 2-6, Supplementary Tables 3-6, 10) to indicate how multiple comparison correction was performed for each model. Our associations with salience/ventral attention network functional connectivity survived multiple comparison correction. Examples of the amendments in the Results and Tables are provided below (amendments are in bold):

Results (pp. 14, lines 303-307) (example)

"Higher handgrip strength in older adults was associated with better global cognition, processing speed and attention performance (FDR-adjusted $p < 0.05$, **corrected for multiple comparisons across the five cognitive measures**)"

Table 2 (example)

Table 2: Associations between handgrip strength and cognitive measures

Cognitive measure	Coefficient	SE	*t*	Uncorr *p*	FDR-adj *p*
Global	0.251	0.076	3.30	0.0012*	0.0059*
Processing speed	0.327	0.106	3.09	0.0024*	0.0059*
Attention	0.237	0.098	2.41	0.0172*	0.0287*
Executive function	0.187	0.091	2.06	0.0412	0.0516
SM-MMSE	0.043	0.035	1.23	0.2196	0.2196

All models controlled for age, sex and education. **Multiple comparison correction was performed across the five cognitive measures.** * indicates statistically significant effects ($p < 0.05$). Abbreviations: SE, standard error; Uncorr, uncorrected; FDR-adj, false discovery rate-adjusted; SM-MMSE, Singapore-modified version of the mini-mental state examination.

For convenience, a summary of the multiple comparison correction performed for the various analyses is provided below:

Multiple comparison correction in different analyses

Analysis	Number of multiple comparisons corrected for
Associations between handgrip strength and cognitive performance	Five cognitive measures<br>• SM-MMSE scores, composite scores of global cognition, processing speed, attention and executive function
Associations between handgrip strength and network-level system segregation measures	Nine network-level segregation measures<br>• Segregation measures for default, control, limbic, salience/ventral attention, dorsal attention, somatomotor, visual, temporoparietal and subcortical networks

Associations between handgrip strength and S/VA intra-/inter-network functional connectivity	Nine intra- and inter-network functional connectivity measures<br>• S/VA intra-network, S/VA inter-network connectivity to default, control, limbic, dorsal attention, somatomotor, visual, temporoparietal and subcortical networks
Associations between handgrip strength and regional connections within S/VA	231 pairs of regional connections within S/VA
Associations between functional connectivity measures and cognitive performance	Five cognitive measures<br>• SM-MMSE scores, composite scores of global cognition, processing speed, attention and executive function

Abbreviations: SM-MMSE, Singapore-modified version of the mini-mental state examination; S/VA, salience/ventral attention network.

RESULTS

13. Overall results were clear and well-presented. Can authors replace Figure 1A with the location of the nodes used for the salience network? This will help readers for the methods as well.

Response: We thank the reviewer for the suggestion. As mentioned in our response to comment #8, we have included the location and size of all nodes used in our analyses as a supplementary table (Supplementary Table 1). To provide more clarity on the location of the various nodes within the salience/ventral attention network, we have additionally replaced the brain map in Figure 1a with a new map displaying different colours for each region of the salience/ventral attention network. The amended figure is provided below.

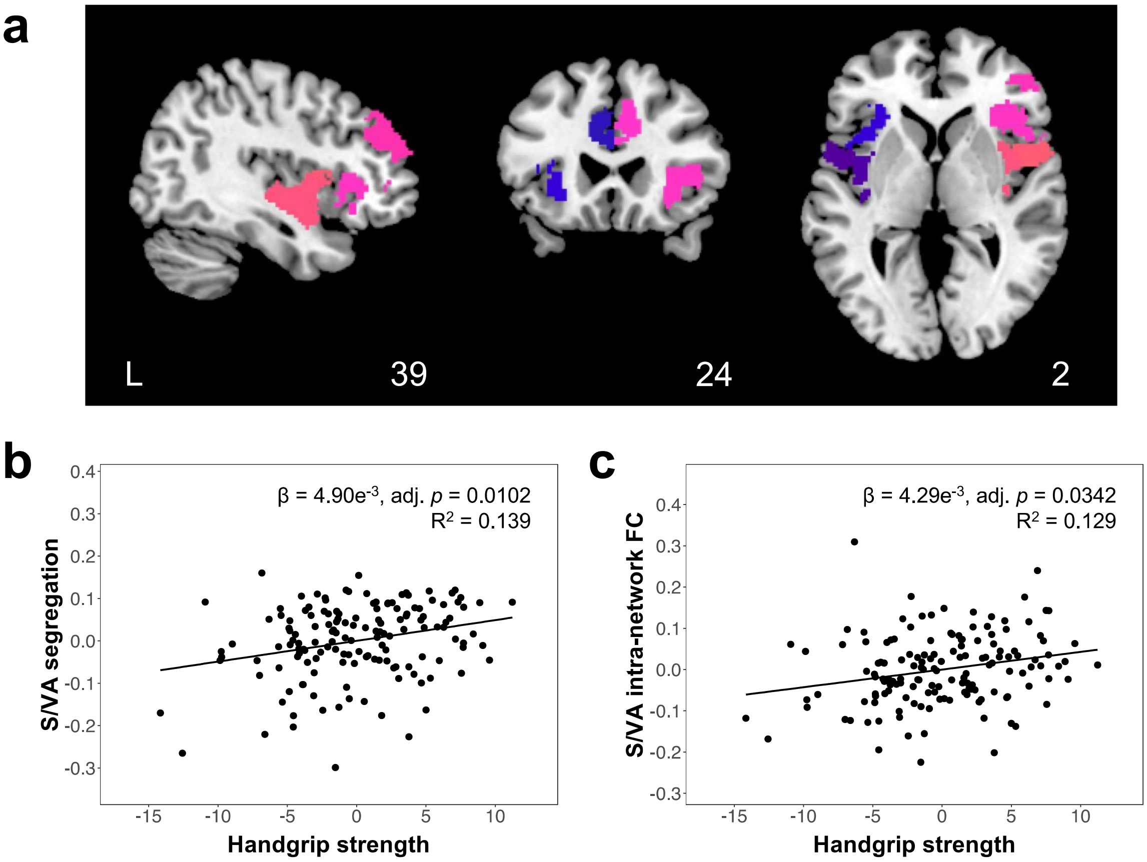

Figure 1. Higher handgrip strength is associated with higher salience/ventral attention network segregation, which is driven by higher salience/ventral attention intra-network connectivity. (a) Brain map depicts brain regions of the salience/ventral attention network overlaid on the Montreal Neurological Institute-152 brain template. Each region within the network is represented by a different colour. (b-c) Scatterplots show associations between handgrip strength residuals and residuals of (b) salience/ventral attention segregation and (c) mean salience/ventral attention intra-network functional connectivity, after controlling for age, sex, education and total intracranial volumes. Higher handgrip strength was linked to higher segregation of the salience/ventral attention network. Further examination of handgrip strength associations with salience/ventral attention inter- and intra-network functional connectivity revealed an association between higher handgrip strength and higher mean salience/ventral intra-network connectivity, suggesting that handgrip strength-related inter-individual differences in segregation of the salience/ventral attention network were driven by its intra-network connections. Abbreviations: adj., adjusted; S/VA, salience/ventral attention; FC, functional connectivity.

14. Although the correlations between network segregation and hand grip strength are significant, they are quite small in terms of percent variance accounted for. This should be discussed; is this really a meaningful relationship? Moreover, the results are largely driven by within network connectivity. This is not segregation per se but rather connectivity strength within a given network, which has been extensively examined.

Response: We appreciate this insightful point from the reviewer. Our study's approach was to examine associations between handgrip strength and functional

segregation in a top-down manner. First, we examined relationships between handgrip strength and functional segregation in a network-agnostic manner (i.e., we examined segregation of all networks across the brain). Subsequently, for networks whose segregation showed significant associations with handgrip strength, we then sought to examine if inter- or intra-network functional connections (both mean connectivity as well as regional connections of the network) of the network were driving the handgrip strength-related variability in segregation. Our study thus differed from other studies in that we did not restrict our analyses to specific networks (e.g., somatomotor network) or to particular network measures (e.g., intra-network connectivity).

Using this approach, we showed that salience/ventral attention network segregation but not segregation of other networks was associated with handgrip strength. Additionally, we demonstrated that salience/ventral attention network segregation was associated with processing speed. As such, we believe that our findings are meaningful because it highlighted the particular involvement of the salience network, but not other networks, in both handgrip strength and cognition.

Further, we revealed that the handgrip strength-related inter-individual differences in salience/ventral attention network segregation were mainly driven by inter-individual differences in intra-network connectivity. While a previous study has also demonstrated an association between salience network integrity and performance on a maximum-force grip task[45], the study only focused on intra-network connectivity. By examining both intra- and inter-network connections of the salience/ventral attention network, our study rigorously demonstrated that it is the salience/ventral attention intra-network connections, but not the inter-network connections, that largely drives the association with handgrip strength.

Moreover, we identified handgrip strength-related inter-individual differences in specific regional connections of the salience/ventral attention network – the right anterior insula connectivity to the left posterior insula/frontal operculum and the right anterior insula connectivity to the right midcingulate/medial parietal cortex – that were found using functional decoding to link to cognitive and somatomotor functions. This finding thus underscores the possibility that the salience/ventral attention network could serve as a neural mechanism underlying inter-individual differences in both handgrip strength and cognition in older individuals.

While we demonstrated that the salience/ventral attention intra-network connections largely drives the association between handgrip strength and salience/ventral attention network segregation, we also observed some associations between handgrip strength and the inter-network connectivity between the salience/ventral attention network and the default and somatomotor network. Although these associations did not pass multiple comparison correction, the results suggest that handgrip strength may relate as well to the balance between intra- and inter-network connectivity of the salience/ventral attention network.

Lastly, we acknowledge that the variance explained is not that high. This is pretty common for functional connectivity studies[46-48]. Further studies are needed to examine the contributions of various functional and structural brain measures to handgrip strength. This is however beyond the scope of the current study.

We have revised the Discussion in the manuscript (pp. 17 lines 373-382, pp. 18-19 lines 400-405, 411-424) to emphasize the advantages of our approach. The revised sections are provided below (amendments are in bold):

Discussion (pp. 17, lines 373-382)

"Our study sought to examine the relationships linking handgrip strength, brain functional connectivity and cognition in older adults. **Unlike previous studies which focused on specific networks, we examined associations between handgrip strength and functional segregation in a whole-brain, network-agnostic manner, first identifying networks whose functional segregation were related to handgrip strength. Subsequently, for these identified networks, we sought to examine if handgrip strength-related inter-individual differences in functional segregation of the network were driven by specific inter- or intra-network functional connections.** We demonstrated that higher handgrip strength was associated with better processing speed, global cognition and attention performance, as well as higher functional segregation of the salience/ventral attention network, **but not other networks**, in older adults."

Discussion (pp. 18-19, lines 400-405, 411-424)

"Previous studies examining motor or physical frailty-related inter-individual differences in functional connectivity have focused on the functional connectivity of specific regions or networks. In this study, we have instead examined how **handgrip strength was related to both global and network-level functional segregation of all networks in the brain**, which may provide greater insight on the neural basis of the relationship between handgrip strength and cognition."

"Adding to the current literature on the neural correlates of handgrip strength, our findings suggest that inter-individual differences in handgrip strength in older adults **were selectively related** to functional connectivity inter-individual differences of specific networks (i.e., salience/ventral attention network), rather than global inter-individual differences across several functional networks. Specifically, our findings suggest that lower handgrip strength in older adults is related to a disruption in the balance of intra- and inter-network connections of the salience/ventral attention network, consequently resulting in lower functional specialization and poorer ability of the network to carry out its specialized function. **Notably, by further examining handgrip strength-related inter-individual differences in intra- and inter-network connectivity, we revealed that this disruption in the balance between intra- and inter-network connections of the salience/ventral attention network was driven largely by disruptions in intra-network connectivity, along with some, albeit weaker (i.e., did not pass multiple comparison correction) disruptions of inter-network connectivity to default and somatomotor networks.**"

15. The authors refer to both sensorimotor and somatomotor networks throughout the paper; are these meant to be the same network?

Response: We thank the reviewer for the comment. The somatomotor and sensorimotor networks are meant to refer to the same network. As such, we have amended the manuscript to standardize the term for this network to “somatomotor network”.

DISCUSSION

16. Pg 16, second paragraph is repetitive of the first paragraph, and re-summarizes the findings. It would be beneficial to expand on the cognition x motor association and its implications.

Response: We thank the reviewer for the useful feedback. As recommended, we have extensively revised the manuscript to provide a more comprehensive discussion about the associations between handgrip strength, salience/ventral attention network functional connectivity and cognitive performance. In particular, we focused on the associations with processing speed, which was more strongly related to both handgrip strength and salience/ventral attention network functional connectivity than the other two cognitive domains (executive function and attention). We cited previous studies that supported the salience/ventral attention network-processing speed and handgrip strength-processing speed associations, as well as highlighted the possibility of training handgrip strength to improve cognitive performance. Additionally, we discussed the potential influence of age on our associations. The revised Discussion (pp. 21-23, lines 477-521) is provided below:

“Importantly, our findings demonstrated that both handgrip strength and salience/ventral attention network functional connectivity were related to cognitive performance, highlighting the links between handgrip strength, cognition and salience/ventral attention network connectivity. Specifically, we found both handgrip strength and salience/ventral attention network functional connectivity to be associated with general cognition (an average measure of the three cognitive domains) and processing speed, but showed either weaker or no association with the other cognitive domains (i.e., attention and executive function). Processing speed, which measures the time taken to perform a mental task[49], is among the strongest predictors of performance across various cognitive tasks[50], and has been demonstrated to explain a large proportion of age-related variance in a wide range of cognitive tasks[51]. In a study examining handgrip strength associations with 30 behavioural outcomes in >40,000 participants from the UK Biobank, processing speed (measured as reaction time) was found to show the most robust association with handgrip strength[52], which the authors partly attributed to the strong dependence of the reaction time task on motor speed and dexterity (both closely associated with the muscular function of the hands)[53]. Several studies have also demonstrated that short

bouts of isometric handgrip exercises can temporarily improve reaction times in an auditory oddball task[54], an auditory n-back task[55] and a Go/No-Go task[56], underscoring the link between handgrip strength and processing speed, as well as raising the possibility of training handgrip strength to improve processing speed. As for the salience/ventral attention network, lower salience/ventral attention network functional connectivity has been found to relate to age-related decreases in visual processing speed (i.e., the rate of visual information uptake)[57]. Interestingly, visual processing speed has been proposed to be closely linked to tonic alertness[58]. In a follow-up study, alertness training in healthy older adults was found to improve visual processing speed, with pre-training salience/ventral attention network functional connectivity correlated with the extent of training-induced change in visual processing speed[59]. These findings thus provide further support for the role of salience/ventral attention network functional connectivity and tonic alertness on both processing speed and handgrip strength. Collectively, these findings also raise the possibility that interventions targeting handgrip strength could potentially improve cognitive performance in older adults via modifications of salience/ventral attention network functional connectivity, although further intervention studies need to be done to evaluate the long-term effects of handgrip strength training on functional connectivity and cognitive performance. Notwithstanding, other studies have also reported performance in other cognitive domains (e.g., working memory, executive function) to be related to handgrip strength[15-19] and salience/ventral attention network functional connectivity[60,61]. As such, more studies are needed to shed light on the neural mechanisms underlying the associations between handgrip strength and performance in various cognitive domains. It is possible that the more robust associations with processing speed are also partly explained by age-related changes in processing speed, given that processing speed is highly sensitive to ageing and has been widely demonstrated to show robust age-related declines[51,62,63]. Indeed, exploratory analyses revealed that age was most strongly associated with both processing speed and global cognition (Supplementary Table 11), suggesting possible influences of age on these associations. In our study, we have attempted to account for the influence of age in all our analyses, however future longitudinal studies might be more equipped to disentangle the influence of age on the handgrip strength-brain-cognition associations from other non-age-related sources."

Supplementary Table 11: Associations between age and cognitive measures

Cognitive measure	Coefficient	SE	*t*	Uncorr *p*	FDR-adj *p*
Global	-0.502	0.103	-4.86	<0.0001*	<0.0001*
Processing speed	-0.665	0.143	-4.65	<0.0001*	<0.0001*
Attention	-0.349	0.132	-2.65	0.0090*	0.0112*
Executive function	-0.493	0.121	-4.08	0.0001*	0.0001*
SM-MMSE	-0.110	0.046	-2.37	0.0192*	0.0192*

All models controlled for sex and education. Multiple comparison correction was performed across the five cognitive measures. * indicates statistically significant effects ($p < 0.05$). Abbreviations: SE, standard error;

Uncorr, uncorrected; FDR-adj, false discovery rate-adjusted; SM-MMSE, Singapore-modified version of the mini-mental state examination.

17. This study used several measures of cognitive performance, but it does not explain or discuss the outcomes and implications for the different measures used. This is touched upon very briefly in pg 17, but a clear paragraph on why global cognition and processing speed but not others were associated with functional connectivity, and implications for the findings will be helpful.

Response: We thank the reviewer for this important comment. As mentioned in our response to comment #16 above, our revised manuscript (pp. 21-23, lines 477-521) has included a more comprehensive discussion of the associations between handgrip strength, salience/ventral attention network functional connectivity and cognitive performance. We focused on the association with processing speed instead of global cognition, because global cognition reflects the average performance across the three cognitive domains and includes contributions from processing speed which shows the most robust age-related decline. We highlighted previous evidence linking salience/ventral attention network functional connectivity to processing speed, and also discussed the implications of these findings. Further, we discussed the possible influence of age on the cognitive associations. The revised Discussion (pp. 21-23, lines 477-521) is provided in our response to the previous comment (comment #16).

18. I believe one of the unique parts of this study is that the authors delved deeper into the segregation score to determine what part of the segregation score was associated to motor and cognitive function. Some of it is addressed on pg 17, but can the authors expand on why the associations were driven by intra-network connectivity? Again, this is not really network segregation in this case but just within network connectivity strength.

Response: We thank the reviewer for this comment. As suggested by the reviewer, we have provided a more in-depth discussion (Discussion, pp. 20-21, lines 456-476) addressing the observations that the handgrip strength-segregation associations were largely driven by inter-individual differences in salience/ventral attention intra-network connectivity. In particular, we highlighted the link between vigilance (or tonic alertness) and handgrip strength, as well as the selective involvement of the salience/ventral attention network in tonic alertness. Interestingly, a vigilance network has previously been identified, comprising regions from salience/ventral attention, somatomotor and default networks[64,65]. These findings are consistent with our findings of handgrip strength associations with salience/ventral attention intra-network connectivity and to a lesser extent, salience/ventral attention inter-network connectivity to both the default and somatomotor networks. Additionally, the lack of handgrip strength associations with salience/ventral attention inter-network connectivity to other networks (such as control and dorsal attention networks) suggests that handgrip strength may be less related to other aspects of attention (e.g., executive control) which involves both the

salience/ventral attention network and other task-positive networks[66]. Our findings thus indicate a possible association between handgrip strength and vigilance/tonic alertness that is mediated by changes in salience/ventral attention network connectivity. Such conjectures are however preliminary, and more research needs to be done to explore the relationships between handgrip strength and various aspects of attention as well as the neural mechanisms underlying these relationships. The revised Discussion (pp. 20-21, lines 456-476) is provided below:

"In particular, performance of an isometric handgrip exercise concurrently elicits spontaneous pupil diameter fluctuations[54,55], which have been shown to track vigilance[67,68]. Vigilance, or tonic alertness, describes the intrinsic capacity to sustain attention for a period of time[69] and is part of the alerting system, one of three systems (the other two being orienting and executive control) proposed by Posner and Petersen to be involved in attention[66,70]. Notably, tonic alertness has been proposed to be selectively subserved by the salience/ventral attention network and not the other task-positive networks such as the control or dorsal attention networks[69]. Additionally, a vigilance network has previously been identified, comprising regions from salience/ventral attention, somatomotor and default networks[64,65]. These findings align with our findings of handgrip strength associations with salience/ventral attention intra-network connectivity and to a lesser extent, salience/ventral attention inter-network connectivity to both the default and somatomotor networks, and hence suggest a possible link between handgrip strength and vigilance/tonic alertness that is mediated by changes in salience/ventral attention network connectivity. Furthermore, we did not find any associations between handgrip strength and salience/ventral attention inter-network connectivity to other networks, indicating that handgrip strength may be less related to other aspects of attention such as executive control, which involves both the salience/ventral attention network and other task-positive networks (e.g., control and dorsal attention networks)[66]. These conjectures are however preliminary, and more research needs to be done to examine the relationships between handgrip strength and various aspects of attention as well as the neural mechanisms underlying these relationships."

19. Similar to the comment for the introduction, in the last paragraph in pg 18, the authors indicate that they did not find associations with the sensorimotor network segregation due to the functional connectivity measure used. However, in Cassady et al., 2020 (Neuroimage, PMID 32109601), and Cassady et al., 2019 (Neuroimage, PMID: 30414983) sensorimotor network segregation is associated with sensorimotor performance that includes grip-strength. Can the authors expand on why sensorimotor network segregation score was not associated with motor (and cognitive) function?

Response: We thank the reviewer for the comment. While Cassady and colleagues have reported an association between somatomotor network segregation and a handgrip strength factor (identified using factor analysis on several somatomotor

measures)[29,30], the handgrip strength factor examined in both their studies included contributions from measures of endurance in addition to handgrip strength. When handgrip strength was instead examined as a standalone measure (same as our current study), Cassady and colleagues similarly reported only a trend towards a positive relationship between somatomotor network segregation and handgrip strength (refer to Table S10 in their paper)[30]. Given that previous studies have also demonstrated associations between somatomotor network functional connectivity and other somatomotor measures[24-26,29,30], these findings collectively raise the possibility that handgrip strength might be related to neural mechanisms that differ from that of other somatomotor measures. However, further studies are needed to examine the differential brain functional and structural brain changes associated with changes in various somatomotor functions. We have updated the revised manuscript to include these points in the Discussion (pp. 23, lines 522-540, provided below).

"Surprisingly, although previous studies have demonstrated associations between somatomotor network functional connectivity and handgrip strength[29,30,71], we found only a trend towards a positive association between handgrip strength and somatomotor network segregation. The discrepancy in findings could likely be attributed to either differences in the type of functional connectivity measure examined (functional segregation in our study versus functional connectivity of specific networks in the previous study)[71] or differences in the handgrip strength measure used[29,30]. With regards to the latter, although Cassady and colleagues reported an association between somatomotor network segregation and a handgrip strength factor (identified using factor analysis on several somatomotor measures), the handgrip strength factor examined in their studies included not only contributions from handgrip strength but also measures of endurance[29,30]. When handgrip strength was instead examined as a standalone measure, Cassady and colleagues similarly reported only a trend towards a positive relationship between somatomotor network segregation and handgrip strength[30]. Given that other studies have also found associations between somatomotor network functional connectivity and other somatomotor measures[24-26,29,30], these findings collectively indicate a possibility that handgrip strength might be related to neural mechanisms that differ from that of other somatomotor functions. Further studies are needed to examine the differential brain functional and structural changes associated with changes in various somatomotor functions."

Minor comments:

20. Pg 18, ln 418, please cite these studies

Response: As suggested by the reviewer, we have included and cited these studies in the Discussion (pp. 23, lines 522-540) (refer to our response to comment #19 above).

Reviewer #2 (Remarks to the Author):

1. The authors establish associations of functional connectivity with cognition and grip strength but the three variables are never included in the same model to test, for example, whether the variance shared between functional connectivity and grip strength is also shared by cognition (whether one mediates the other).

Response: We thank the reviewer for the insightful comment. As described in the Limitations (pp. 23-24, lines 541-553), we did not perform any mediation analyses in the study due to the cross-sectional nature of our study, which does not allow us to ascertain the temporal/causal relationships between handgrip strength and cognition and consequently determine the dependent and independent variables in the mediation model. However, we are currently in the midst of collecting longitudinal neuroimaging, cognitive and physical performance data for a new cohort of community-dwelling older adults, which would enable us to examine the directionality of the association between handgrip strength and cognition, as well as the mediation effect of functional connectivity on these handgrip strength-cognition associations.

Despite these limitations, we believe that our current work continues to provide valuable insight on the potential neural underpinnings of the relationship between handgrip strength and cognition in older adults. Unlike previous studies which have focused only on specific networks (e.g., somatomotor network), our study examined associations between handgrip strength and functional segregation in a whole-brain, network-agnostic manner. We demonstrated that higher handgrip strength was selectively associated with higher functional segregation of the salience/ventral attention network, but not the other networks. Further, we showed that this higher handgrip strength-related segregation of the salience/ventral attention network was driven mainly by higher salience/ventral attention intra-network connectivity. Importantly, these handgrip strength-related inter-individual differences in salience/ventral attention network functional connectivity were linked to cognitive function, highlighting the particular importance of the salience/ventral attention network in both handgrip strength and cognition.

2. The authors acknowledge that the cross-sectional nature of the study is a limitation. Are there any relevant variables in the study that have been collected longitudinally and could be incorporated to better understand sequences of events?

Response: We thank the reviewer for the comment. Unfortunately, we do not have the longitudinal data required to investigate the sequence of events (i.e., whether declines in handgrip strength precede cognitive decline and vice versa). However, as mentioned in our response to the previous comment (comment #1), we are currently collecting new longitudinal data in older adults and we hope to be able to examine the

directionality of the handgrip strength-cognition relationship in the new cohort once the data is ready.

3. The time between grip strength assessment and MRI is reported to be within 18 months. Specify why the gap is so long, whether the results hold when time between measures is included as a covariate and whether one assessment systematically preceded the other.

Response: We thank the reviewer for the comment. As addressed in our response to reviewer #1 (comment #9), we have plotted the distribution of the time interval between the MRI scan and handgrip strength assessment (i.e., handgrip strength measurement date – MRI scan date), as well as re-ran the analyses including the time interval as a covariate. MRI scans generally preceded the handgrip strength assessments, with handgrip strength assessments conducted on average 276.56 (SD = 156.64, range = -20 – 547) days after the MRI scans.

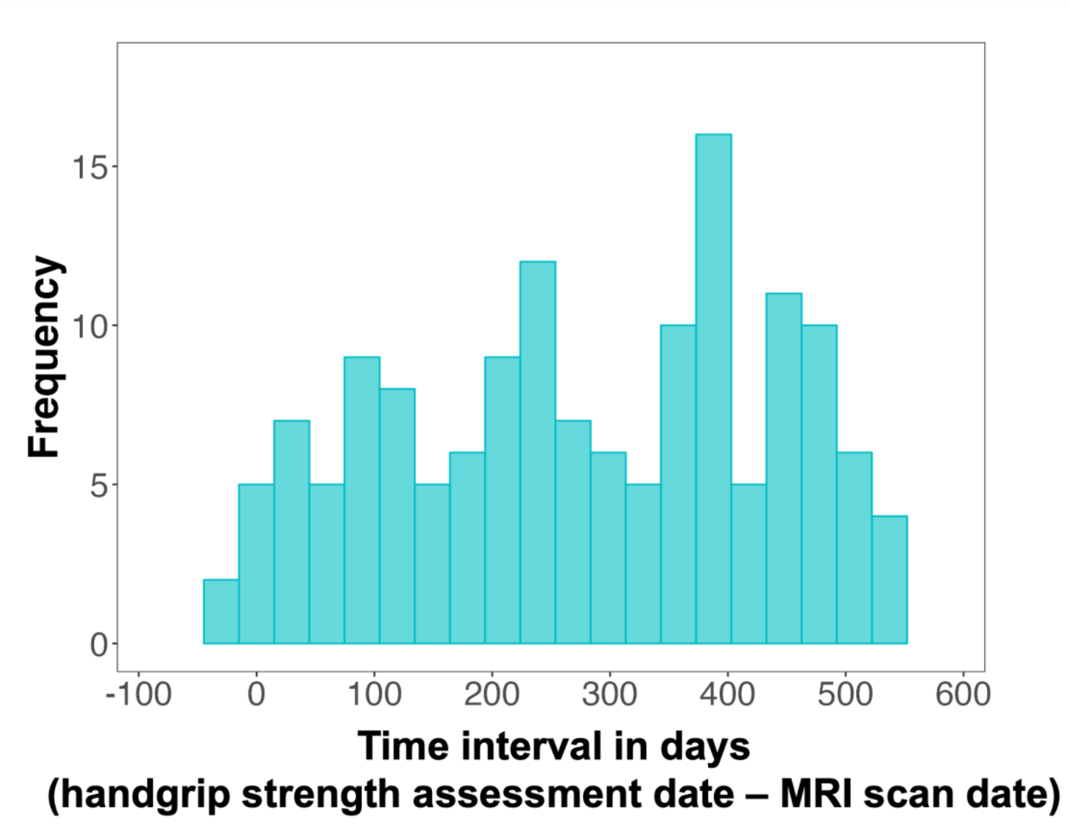

Supplementary Figure 1. Time interval between date of MRI scan and date of handgrip strength assessments. Histogram displays the time interval (in days) between the MRI scan and handgrip strength assessment (handgrip strength assessment date – MRI scan date). On average, handgrip strength assessments were performed 276.56 (SD = 156.64, range = -20 – 547) days after the MRI scans.

Our results also remained even after controlling for the time interval between MRI scan and handgrip strength assessment, suggesting little influence of the time interval

between MRI scan and handgrip strength assessment on associations between handgrip strength and functional connectivity. These results (both the histogram and validation analysis controlling for the time interval) have been included in the following sections: Methods: pp. 13-14, lines 295-300, Results: pp. 15, lines 343-345, Supplementary Figure 1 (see above figure), and Supplementary Tables 4-6. The revised sections and supplementary tables are also provided below (amendments are in bold):

Methods (pp. 13-14, lines 295-300)

"**Lastly, handgrip strength assessments were found to be conducted on average 276.56 (SD = 156.64, range = -20 – 547) days after the MRI scans (Supplementary Fig. 1). Given this time difference between the MRI scan and handgrip assessment, we repeated the handgrip strength-functional connectivity association analyses with time interval (days) between the MRI scan and handgrip strength assessment included as an additional covariate.**"

Results (pp. 15, lines 343-345)

"All these findings remained after controlling for SM-MMSE scores, Geriatric Depression Scale scores, body mass index, waist-hip ratio **or time interval between MRI scan and handgrip strength assessment** (Supplementary Tables 4-6)."

Supplementary Table 4: Validation analyses: associations between handgrip strength and global and network-level system segregation

**Measure**	**Coefficient**	**SE**	***t***	**Uncorr *p***	**FDR-adj *p***
**Analysis 1: Analysis additionally controlling for SM-MMSE (n = 148)**					
Global segregation	$1.80e^{-3}$	$8.86e^{-4}$	2.03	0.0439*	0.0439*
Network segregation					
Default	$2.41e^{-3}$	$1.53e^{-3}$	1.57	0.1187	0.2916
Control	$6.78e^{-4}$	$1.65e^{-3}$	0.41	0.6815	0.7667
Limbic	$1.25e^{-3}$	$1.83e^{-3}$	0.68	0.4971	0.6541
Salience/ventral attention	$4.89e^{-3}$	$1.49e^{-3}$	3.29	0.0013*	0.0115*
Dorsal attention	$1.27e^{-3}$	$1.92e^{-3}$	0.66	0.5088	0.6541
Somatomotor	$2.89e^{-3}$	$1.70e^{-3}$	1.70	0.0909	0.2916
Visual	$2.89e^{-4}$	$1.09e^{-3}$	0.27	0.7905	0.7905
Temporoparietal	$5.26e^{-3}$	$3.45e^{-3}$	1.52	0.1296	0.2916
Subcortical	$1.25e^{-3}$	$1.24e^{-3}$	1.01	0.3137	0.5646
**Analysis 2: Analysis additionally controlling for waist-hip ratio (n = 148)**					
Global segregation	$1.68e^{-3}$	$8.90e^{-4}$	1.89	0.0607	0.0607
Network segregation					
Default	$2.34e^{-3}$	$1.54e^{-3}$	1.52	0.1298	0.2920
Control	$8.55e^{-4}$	$1.65e^{-3}$	0.52	0.6041	0.6796
Limbic	$1.22e^{-3}$	$1.83e^{-3}$	0.67	0.5056	0.6796
Salience/ventral attention	$5.01e^{-3}$	$1.48e^{-3}$	3.37	0.0010*	0.0086*
Dorsal attention	$1.11e^{-3}$	$1.93e^{-3}$	0.57	0.5665	0.6796
Somatomotor	$2.84e^{-3}$	$1.70e^{-3}$	1.68	0.0960	0.2920

Visual	$1.44e^{-4}$	$1.08e^{-3}$	0.13	0.8946	0.8946
Temporoparietal	$5.46e^{-3}$	$3.44e^{-3}$	1.59	0.1145	0.2920
Subcortical	$9.79e^{-4}$	$1.24e^{-3}$	0.79	0.4319	0.6796
**Analysis 3: Analysis additionally controlling for body mass index (n = 146)**					
Global segregation	$1.68e^{-3}$	$9.45e^{-4}$	1.78	0.0774	0.0774
Network segregation					
Default	$2.00e^{-3}$	$1.64e^{-3}$	1.22	0.2254	0.4550
Control	$7.16e^{-4}$	$1.73e^{-3}$	0.41	0.6803	0.7654
Limbic	$1.26e^{-3}$	$1.95e^{-3}$	0.65	0.5183	0.6664
Salience/ventral attention	$4.88e^{-3}$	$1.56e^{-3}$	3.12	0.0022*	0.0199*
Dorsal attention	$2.31e^{-3}$	$2.01e^{-3}$	1.15	0.2528	0.4550
Somatomotor	$2.77e^{-3}$	$1.80e^{-3}$	1.54	0.1262	0.3787
Visual	$2.84e^{-4}$	$1.15e^{-3}$	0.25	0.8062	0.8062
Temporoparietal	$6.37e^{-3}$	$3.48e^{-3}$	1.83	0.0693	0.3121
Subcortical	$1.13e^{-3}$	$1.32e^{-3}$	0.86	0.3937	0.5906
**Analysis 4: Analysis additionally controlling for GDS scores (n = 148)**					
Global segregation	$1.66e^{-3}$	$8.80e^{-4}$	1.89	0.0613	0.0613
Network segregation					
Default	$2.10e^{-3}$	$1.52e^{-3}$	1.38	0.1704	0.3835
Control	$6.93e^{-4}$	$1.64e^{-3}$	0.42	0.6730	0.7571
Limbic	$1.43e^{-3}$	$1.82e^{-3}$	0.78	0.4343	0.6515
Salience/ventral attention	$4.87e^{-3}$	$1.48e^{-3}$	3.30	0.0012*	0.0112*
Dorsal attention	$1.04e^{-3}$	$1.92e^{-3}$	0.54	0.5897	0.7571
Somatomotor	$2.83e^{-3}$	$1.68e^{-3}$	1.69	0.0939	0.3726
Visual	$2.21e^{-4}$	$1.07e^{-3}$	0.21	0.8372	0.8372
Temporoparietal	$5.22e^{-3}$	$3.38e^{-3}$	1.55	0.1242	0.3726
Subcortical	$1.04e^{-3}$	$1.23e^{-3}$	0.84	0.4007	0.6515
**Analysis 5: Analysis additionally controlling for time interval between MRI scan and handgrip strength measurement (n = 148)**					
**Global segregation**	**$1.52e^{-3}$**	**$8.99e^{-4}$**	**1.69**	**0.0934**	**0.0934**
**Network segregation**					
**Default**	**$2.49e^{-3}$**	**$1.56e^{-3}$**	**1.60**	**0.1127**	**0.3203**
**Control**	**$-4.20e^{-5}$**	**$1.64e^{-3}$**	**-0.03**	**0.9796**	**0.9796**
**Limbic**	**$1.36e^{-3}$**	**$1.86e^{-3}$**	**0.73**	**0.4675**	**0.7344**
**Salience/ventral attention**	**$4.33e^{-3}$**	**$1.49e^{-3}$**	**2.91**	**0.0042***	**0.0378***
**Dorsal attention**	**$1.10e^{-3}$**	**$1.96e^{-3}$**	**0.56**	**0.5761**	**0.7406**
**Somatomotor**	**$3.31e^{-3}$**	**$1.71e^{-3}$**	**1.94**	**0.0549**	**0.2472**
**Visual**	**$-4.08e^{-4}$**	**$1.06e^{-3}$**	**-0.38**	**0.7015**	**0.7892**
**Temporoparietal**	**$5.16e^{-3}$**	**$3.50e^{-3}$**	**1.48**	**0.1424**	**0.3203**
**Subcortical**	**$8.72e^{-4}$**	**$1.26e^{-3}$**	**0.69**	**0.4896**	**0.7344**

Multiple comparison correction was performed across the nine network-level segregation measures. * indicates statistically significant effects ($p < 0.05$). Abbreviations: SE, standard error; Uncorr, uncorrected; FDR-adj, false discovery rate-adjusted; SM-MMSE, Singapore-modified version of the mini-mental state examination; GDS, geriatric depression scale scores.

Supplementary Table 5: Validation analyses: associations between handgrip strength and mean salience/ventral attention inter- and intra-network functional connectivity

Measure	Coefficient	SE	*t*	Uncorr *p*	FDR-adj *p*
**Analysis 1: Analysis additionally controlling for SM-MMSE (n = 148)**					
Intra-network S/VA	$4.21e^{-3}$	$1.47e^{-3}$	2.87	0.0048*	0.0428*
Inter-network					
S/VA – Default	$-3.04e^{-3}$	$1.40e^{-3}$	-2.18	0.0312*	0.0935
S/VA – Control	$-9.52e^{-4}$	$1.07e^{-3}$	-0.89	0.3740	0.4927
S/VA – Limbic	$-8.40e^{-4}$	$1.45e^{-3}$	-0.58	0.5636	0.5636
S/VA – Dorsal attention	$1.20e^{-3}$	$1.37e^{-3}$	0.87	0.3832	0.4927
S/VA – Somatomotor	$3.34e^{-3}$	$1.44e^{-3}$	2.32	0.0215*	0.0935
S/VA – Visual	$2.72e^{-3}$	$1.81e^{-3}$	1.50	0.1347	0.3031
S/VA – Temporoparietal	$1.32e^{-3}$	$1.94e^{-3}$	0.68	0.4991	0.5615
S/VA – Subcortical	$9.88e^{-4}$	$9.31e^{-4}$	1.06	0.2902	0.4927
**Analysis 2: Analysis additionally controlling for waist-hip ratio (n = 148)**					
Intra-network S/VA	$4.47e^{-3}$	$1.46e^{-3}$	3.05	0.0027*	0.0243*
Inter-network					
S/VA – Default	$-3.14e^{-3}$	$1.39e^{-3}$	-2.26	0.0253*	0.0758
S/VA – Control	$-9.73e^{-4}$	$1.07e^{-3}$	-0.91	0.3632	0.5067
S/VA – Limbic	$-8.59e^{-4}$	$1.45e^{-3}$	-0.59	0.5550	0.5550
S/VA – Dorsal attention	$1.57e^{-3}$	$1.33e^{-3}$	1.18	0.2414	0.4345
S/VA – Somatomotor	$3.45e^{-3}$	$1.44e^{-3}$	2.40	0.0176*	0.0758
S/VA – Visual	$2.66e^{-3}$	$1.81e^{-3}$	1.46	0.1452	0.3266
S/VA – Temporoparietal	$1.47e^{-3}$	$1.94e^{-3}$	0.76	0.4504	0.5067
S/VA – Subcortical	$7.69e^{-4}$	$9.23e^{-4}$	0.83	0.4062	0.5067
**Analysis 3: Analysis additionally controlling for body mass index (n = 146)**					
Intra-network S/VA	$4.37e^{-3}$	$1.56e^{-3}$	2.81	0.0057*	0.0510
Inter-network					
S/VA – Default	$-2.48e^{-3}$	$1.47e^{-3}$	-1.68	0.0946	0.2839
S/VA – Control	$-3.66e^{-4}$	$1.13e^{-3}$	-0.32	0.7475	0.7475
S/VA – Limbic	$-6.59e^{-4}$	$1.54e^{-3}$	-0.43	0.6683	0.7475
S/VA – Dorsal attention	$1.20e^{-3}$	$1.42e^{-3}$	0.84	0.4018	0.6925
S/VA – Somatomotor	$3.06e^{-3}$	$1.52e^{-3}$	2.01	0.0461*	0.2075
S/VA – Visual	$2.07e^{-3}$	$1.93e^{-3}$	1.07	0.2845	0.6401
S/VA – Temporoparietal	$1.15e^{-3}$	$2.04e^{-3}$	0.56	0.5743	0.7384
S/VA – Subcortical	$7.18e^{-4}$	$9.73e^{-4}$	0.74	0.4617	0.6925
**Analysis 4: Analysis additionally controlling for GDS scores (n = 148)**					
Intra-network S/VA	$4.30e^{-3}$	$1.46e^{-3}$	2.94	0.0038*	0.0345*
Inter-network					
S/VA – Default	$-2.74e^{-3}$	$1.39e^{-3}$	-1.98	0.0499*	0.1496
S/VA – Control	$-7.28e^{-4}$	$1.07e^{-3}$	-0.68	0.4961	0.5425
S/VA – Limbic	$-8.81e^{-4}$	$1.44e^{-3}$	-0.61	0.5425	0.5425
S/VA – Dorsal attention	$1.08e^{-3}$	$1.36e^{-3}$	0.80	0.4261	0.5425
S/VA – Somatomotor	$3.32e^{-3}$	$1.43e^{-3}$	2.33	0.0214*	0.0963
S/VA – Visual	$2.38e^{-3}$	$1.81e^{-3}$	1.31	0.1910	0.4298
S/VA – Temporoparietal	$1.33e^{-3}$	$1.91e^{-3}$	0.70	0.4879	0.5425
S/VA – Subcortical	$9.51e^{-4}$	$9.26e^{-4}$	1.03	0.3061	0.5425
**Analysis 5: Analysis additionally controlling for time interval between MRI scan and handgrip strength measurement (n = 148)**					
**Intra-network S/VA**	**$3.97e^{-3}$**	**$1.48e^{-3}$**	**2.67**	**0.0084***	**0.0753**

**Inter-network**					
**S/VA – Default**	**-2.65e^{-3}**	**1.43e^{-3}**	**-1.85**	**0.0658**	**0.1975**
**S/VA – Control**	**-7.25e^{-4}**	**1.09e^{-3}**	**-0.66**	**0.5081**	**0.5716**
**S/VA – Limbic**	**-1.21e^{-3}**	**1.47e^{-3}**	**-0.82**	**0.4117**	**0.5294**
**S/VA – Dorsal attention**	**1.19e^{-3}**	**1.39e^{-3}**	**0.85**	**0.3943**	**0.5294**
**S/VA – Somatomotor**	**3.05e^{-3}**	**1.45e^{-3}**	**2.10**	**0.0372***	**0.1674**
**S/VA – Visual**	**2.59e^{-3}**	**1.85e^{-3}**	**1.40**	**0.1623**	**0.3653**
**S/VA – Temporoparietal**	**7.60e^{-4}**	**1.96e^{-3}**	**0.39**	**0.6986**	**0.6986**
**S/VA – Subcortical**	**1.11e^{-3}**	**9.42e^{-4}**	**1.18**	**0.2399**	**0.4319**

Multiple comparison correction was performed across the nine salience/ventral attention inter- and intra-network functional connectivity measures. * indicates statistically significant effects ($p < 0.05$). Abbreviations: SE, standard error; Uncorr, uncorrected; FDR-adj, false discovery rate-adjusted; S/VA, salience/ventral attention; SM-MMSE, Singapore-modified version of the mini-mental state examination; GDS, geriatric depression scale scores.

Supplementary Table 6: Validation analyses: significant associations between handgrip strength and intra-network salience/ventral attention functional connections

Measure	Coefficient	SE	*t*	Uncorr *p*	FDR-adj *p*
**Analysis 1: Analysis additionally controlling for SM-MMSE (n = 148)**					
Right AI – Left PI/FO	1.54e^{-2}	3.86e^{-3}	3.99	0.0001*	0.0177*
Right AI – Right MCC/MPC	1.53e^{-2}	3.92e^{-3}	3.89	0.0002*	0.0177*
**Analysis 2: Analysis additionally controlling for waist-hip ratio (n = 148)**					
Right AI – Left PI/FO	1.62e^{-2}	3.81e^{-3}	4.24	<0.0001*	0.0046*
Right AI – Right MCC/MPC	1.66e^{-2}	3.87e^{-3}	4.30	<0.0001*	0.0046*
**Analysis 3: Analysis additionally controlling for body mass index (n = 146)**					
Right AI – Left PI/FO	1.57e^{-2}	4.09e^{-3}	3.84	0.0002*	0.0191*
Right AI – Right MCC/MPC	1.58e^{-2}	4.11e^{-3}	3.84	0.0002*	0.0191*
**Analysis 4: Analysis additionally controlling for GDS scores (n = 148)**					
Right AI – Left PI/FO	1.53e^{-2}	3.83e^{-3}	4.00	0.0001*	0.0126*
Right AI – Right MCC/MPC	1.55e^{-2}	3.90e^{-3}	3.98	0.0001*	0.0126*
**Analysis 5: Analysis additionally controlling for time interval between MRI scan and handgrip strength measurement (n = 148)**					
**Right AI – Left PI/FO**	**1.44e^{-2}**	**3.89e^{-3}**	**3.70**	**0.0003***	**0.0358***
**Right AI – Right MCC/MPC**	**1.51e^{-2}**	**3.98e^{-3}**	**3.79**	**0.0002***	**0.0358***

Multiple comparison correction was performed across 231 pairs of regional connections within the salience/ventral attention network. * indicates statistically significant effects ($p < 0.05$). Abbreviations: SE, standard error; Uncorr, uncorrected; FDR-adj, false discovery rate-adjusted; SM-MMSE, Singapore-modified version of the mini-mental state examination; GDS, geriatric depression scale scores; AI, anterior insula; PI, posterior insula; FO, frontal operculum; MCC, midcingulate cortex; MPC, medial parietal cortex.

4. Specify what frequencies were included in the bandpass filter.

Response: For the preprocessing of fMRI data, we performed bandpass filtering in the range of 0.009 to 0.1 Hz. We have revised the manuscript (Methods pp. 9, line 199) to include this range.

5. Line 241 specify how many tests are performed.

Response: We performed three functional decoding analyses, with separate analyses done for each of the three salience/ventral attention regions whose regional connections showed associations with handgrip strength: right anterior insula, left posterior insula/frontal operculum and right midcingulate/medial parietal cortex. We have included this information in the Methods (pp. 12 lines 265-277), which is also provided below (amendments are in bold).

"To understand the cognitive and behavioral relevance of brain regions whose functional connections showed associations with handgrip strength, we performed topic-based meta-analytic functional decoding on the binarized map of each brain region using a set of 50 topics extracted with latent Dirichlet allocation topic modelling on the abstracts of all articles in the Neurosynth database[72,73]. **This resulted in a total of three functional decoding analyses performed in the study, with separate analyses done for each of the three salience/ventral attention regions whose regional connections showed correlations with handgrip strength: right anterior insula, left posterior insula/frontal operculum and right midcingulate/medial parietal cortex.** Specifically, we used the Neurosynth ROI association approach on NiMARE v0.0.12 package for Python[74,75], which correlates the mean modeled activation values within the binarized ROI with the term weights of all 50 topics in the Neurosynth dataset. Visualization of these correlation values as word clouds were then performed using the wordcloud package on R[76]."

6. Line 274, the control analyses should be foreshadowed in the introduction and methods. Their motivation and relevance is unclear.

Response: We thank the reviewer for the comment. As described in the Methods (pp. 13-14, lines 288-300), we repeated the analyses including SM-MMSE scores as an additional covariate to ensure that the findings were not influenced by the extent of cognitive impairment in participants. Further, given that handgrip strength has been shown to be associated with depressive symptoms[77,78] as well as measures of obesity such as body mass index[79-82], we repeated the analyses with Geriatric Depression Scale scores, body mass index or waist-hip ratio included as an additional covariate to ensure that the findings were not influenced by depressive symptoms or indices of obesity. Finally, given the relatively long time interval between the handgrip strength measurement and MRI scan, we repeated the analyses controlling for the time interval (days) between the MRI scan and handgrip strength measurement.

We have additionally revised the Introduction (pp. 6-7, lines 129-140) to include a brief description of the validation analyses. The revised Introduction (pp. 6-7, lines 129-140) and Methods (pp. 13, lines 288-300) are provided below (amendments are in bold):

Introduction (pp. 6-7, lines 129-140)

"The aim of the study is therefore to examine the association between handgrip strength and functional segregation of the various networks across the brain in community-dwelling older adults, and how inter-individual differences in the functional segregation of handgrip strength-related networks may be linked to inter-individual differences in cognitive function. Specifically, we hypothesized that higher handgrip strength would be related to higher brain network segregation in the somatomotor and salience networks. Further, we expect that individuals with higher functional segregation in these handgrip strength-related networks would have better cognitive performance. **Finally, we hypothesized that these associations would remain even after accounting for the time difference between MRI scan and handgrip assessment, or the influence of other variables that have been reported to relate to handgrip strength, such as depressive symptoms, indices of obesity or the extent of cognitive impairment in participants.**"

Methods (pp. 13, lines 288-300)

"Validation analyses were also conducted to determine the robustness of the findings (Supplementary Table 2). To ensure that the findings were not influenced by the extent of cognitive impairment in participants, we repeated the analyses with SM-MMSE scores included as an additional covariate. Additionally, handgrip strength has been shown to be associated with depressive symptoms[77,78] as well as measures of obesity such as body mass index[79-82]. To ensure that the findings were not influenced by depressive symptoms or indices of obesity, we repeated the analyses with Geriatric Depression Scale scores, body mass index or waist-hip ratio included as an additional covariate. **Lastly, handgrip strength assessments were found to be conducted on average 276.56 (SD = 156.64, range = -20 – 547) days after the MRI scans (Supplementary Fig. 1). Given this time difference between the MRI scan and handgrip assessment, we repeated the handgrip strength-functional connectivity association analyses with time interval (days) between the MRI scan and handgrip strength assessment included as an additional covariate.**"

REFERENCES

1 Wearing, J., Konings, P., Stokes, M. & de Bruin, E. D. Handgrip strength in old and oldest old Swiss adults - a cross-sectional study. *BMC Geriatr*. **18**, 266, doi:10.1186/s12877-018-0959-0 (2018).

2 Taekema, D. G., Gussekloo, J., Maier, A. B., Westendorp, R. G. & de Craen, A. J. Handgrip strength as a predictor of functional, psychological and social health. A prospective population-based study among the oldest old. *Age Ageing*. **39**, 331-337, doi:10.1093/ageing/afq022 (2010).

3 McGrath, R. P., Kraemer, W. J., Snih, S. A. & Peterson, M. D. Handgrip Strength and Health in Aging Adults. *Sports Med*. **48**, 1993-2000, doi:10.1007/s40279-018-0952-y (2018).

4 Shaughnessy, K. A. *et al.* A Narrative Review of Handgrip Strength and Cognitive Functioning: Bringing a New Characteristic to Muscle Memory. *Journal of Alzheimer's disease : JAD*. **73**, 1265-1278, doi:10.3233/JAD-190856 (2020).

5 Peolsson, A., Hedlund, R. & Oberg, B. Intra- and inter-tester reliability and reference values for hand strength. *J Rehabil Med*. **33**, 36-41, doi:10.1080/165019701300006524 (2001).

6 Kunutsor, S. K., Isiozor, N. M., Voutilainen, A. & Laukkanen, J. A. Handgrip strength and risk of cognitive outcomes: new prospective study and meta-analysis of 16 observational cohort studies. *Geroscience*. **44**, 2007-2024, doi:10.1007/s11357-022-00514-6 (2022).

7 Leong, D. P. *et al.* Prognostic value of grip strength: findings from the Prospective Urban Rural Epidemiology (PURE) study. *Lancet*. **386**, 266-273, doi:10.1016/S0140-6736(14)62000-6 (2015).

8 Halaweh, H. Correlation between Health-Related Quality of Life and Hand Grip Strength among Older Adults. *Exp Aging Res*. **46**, 178-191, doi:10.1080/0361073X.2020.1716157 (2020).

9 Kwak, Y. & Kim, Y. Quality of life and subjective health status according to handgrip strength in the elderly: a cross-sectional study. *Aging Ment Health*. **23**, 107-112, doi:10.1080/13607863.2017.1387766 (2019).

10 Chua, K. Y., Lim, W. S., Lin, X., Yuan, J. M. & Koh, W. P. Handgrip Strength and Timed Up-and-Go (TUG) Test are Predictors of Short-Term Mortality among Elderly in a Population-Based Cohort in Singapore. *J Nutr Health Aging*. **24**, 371-378, doi:10.1007/s12603-020-1337-0 (2020).

11 Ling, C. H. *et al.* Handgrip strength and mortality in the oldest old population: the Leiden 85-plus study. *CMAJ*. **182**, 429-435, doi:10.1503/cmaj.091278 (2010).

12 Malhotra, R., Tareque, M. I., Tan, N. C. & Ma, S. Association of baseline hand grip strength and annual change in hand grip strength with mortality among

older people. *Arch Gerontol Geriatr*. **86**, 103961, doi:10.1016/j.archger.2019.103961 (2020).

13 McGrath, R. *et al.* Handgrip Strength Asymmetry and Weakness Together Are Associated With Functional Disability in Aging Americans. *J Gerontol A Biol Sci Med Sci*. **76**, 291-296, doi:10.1093/gerona/glaa100 (2021).

14 Rantanen, T. *et al.* Midlife hand grip strength as a predictor of old age disability. *Jama*. **281**, 558-560, doi:10.1001/jama.281.6.558 (1999).

15 Zammit, A. R. *et al.* A Coordinated Multi-study Analysis of the Longitudinal Association Between Handgrip Strength and Cognitive Function in Older Adults. *J Gerontol B Psychol Sci Soc Sci*. **76**, 229-241, doi:10.1093/geronb/gbz072 (2021).

16 Zammit, A. R., Robitaille, A., Piccinin, A. M., Muniz-Terrera, G. & Hofer, S. M. Associations Between Aging-Related Changes in Grip Strength and Cognitive Function in Older Adults: A Systematic Review. *J Gerontol A Biol Sci Med Sci*. **74**, 519-527, doi:10.1093/gerona/gly046 (2019).

17 Praetorius Bjork, M., Johansson, B. & Hassing, L. B. I forgot when I lost my grip-strong associations between cognition and grip strength in level of performance and change across time in relation to impending death. *Neurobiology of aging*. **38**, 68-72, doi:10.1016/j.neurobiolaging.2015.11.010 (2016).

18 Sternang, O. *et al.* Grip Strength and Cognitive Abilities: Associations in Old Age. *J Gerontol B Psychol Sci Soc Sci*. **71**, 841-848, doi:10.1093/geronb/gbv017 (2016).

19 Cui, M., Zhang, S., Liu, Y., Gang, X. & Wang, G. Grip Strength and the Risk of Cognitive Decline and Dementia: A Systematic Review and Meta-Analysis of Longitudinal Cohort Studies. *Frontiers in Aging Neuroscience*. **13**, 625551, doi:10.3389/fnagi.2021.625551 (2021).

20 Chupel, M. U. *et al.* Strength Training Decreases Inflammation and Increases Cognition and Physical Fitness in Older Women with Cognitive Impairment. *Frontiers in physiology*. **8**, 377, doi:10.3389/fphys.2017.00377 (2017).

21 Landrigan, J. F., Bell, T., Crowe, M., Clay, O. J. & Mirman, D. Lifting cognition: a meta-analysis of effects of resistance exercise on cognition. *Psychol Res*. **84**, 1167-1183, doi:10.1007/s00426-019-01145-x (2020).

22 Yoon, D. H., Lee, J. Y. & Song, W. Effects of Resistance Exercise Training on Cognitive Function and Physical Performance in Cognitive Frailty: A Randomized Controlled Trial. *J Nutr Health Aging*. **22**, 944-951, doi:10.1007/s12603-018-1090-9 (2018).

23 Carson, R. G. Get a grip: individual variations in grip strength are a marker of brain health. *Neurobiology of aging*. **71**, 189-222, doi:10.1016/j.neurobiolaging.2018.07.023 (2018).

24 Lammers, F. *et al.* Functional Connectivity of the Supplementary Motor Network Is Associated with Fried's Modified Frailty Score in Older Adults. *J Gerontol A Biol Sci Med Sci*. **75**, 2239-2248, doi:10.1093/gerona/glz297 (2020).

25 Lopez-Sanz, D. *et al.* Scoping Review of Neuroimaging Studies Investigating Frailty and Frailty Components. *Front Med (Lausanne)*. **5**, 284, doi:10.3389/fmed.2018.00284 (2018).

26 Yuan, J., Blumen, H. M., Verghese, J. & Holtzer, R. Functional connectivity associated with gait velocity during walking and walking-while-talking in aging: a resting-state fMRI study. *Human brain mapping*. **36**, 1484-1493, doi:10.1002/hbm.22717 (2015).

27 Crowell, C. A. *et al.* Older adults benefit from more widespread brain network integration during working memory. *Neuroimage*. **218**, 116959, doi:10.1016/j.neuroimage.2020.116959 (2020).

28 Grady, C., Sarraf, S., Saverino, C. & Campbell, K. Age differences in the functional interactions among the default, frontoparietal control, and dorsal attention networks. *Neurobiology of aging*. **41**, 159-172, doi:10.1016/j.neurobiolaging.2016.02.020 (2016).

29 Cassady, K. *et al.* Network segregation varies with neural distinctiveness in sensorimotor cortex. *Neuroimage*. **212**, 116663, doi:10.1016/j.neuroimage.2020.116663 (2020).

30 Cassady, K. *et al.* Sensorimotor network segregation declines with age and is linked to GABA and to sensorimotor performance. *Neuroimage*. **186**, 234-244, doi:10.1016/j.neuroimage.2018.11.008 (2019).

31 Chan, M. Y., Park, D. C., Savalia, N. K., Petersen, S. E. & Wig, G. S. Decreased segregation of brain systems across the healthy adult lifespan. *Proceedings of the National Academy of Sciences of the United States of America*. **111**, E4997-5006, doi:10.1073/pnas.1415122111 (2014).

32 Ferreira, L. K. *et al.* Aging Effects on Whole-Brain Functional Connectivity in Adults Free of Cognitive and Psychiatric Disorders. *Cerebral cortex*. **26**, 3851-3865, doi:10.1093/cercor/bhv190 (2016).

33 Zhang, H. Y. *et al.* Selective vulnerability related to aging in large-scale resting brain networks. *PloS one*. **9**, e108807, doi:10.1371/journal.pone.0108807 (2014).

34 Chong, J. S. X. *et al.* Longitudinal Changes in the Cerebral Cortex Functional Organization of Healthy Elderly. *The Journal of neuroscience : the official journal of the Society for Neuroscience*. **39**, 5534-5550, doi:10.1523/JNEUROSCI.1451-18.2019 (2019).

35 Betzel, R. F. *et al.* Changes in structural and functional connectivity among resting-state networks across the human lifespan. *Neuroimage*. **102 Pt 2**, 345-357, doi:10.1016/j.neuroimage.2014.07.067 (2014).

36 Geerligs, L., Renken, R. J., Saliasi, E., Maurits, N. M. & Lorist, M. M. A Brain-Wide Study of Age-Related Changes in Functional Connectivity. *Cerebral cortex*. **25**, 1987-1999, doi:10.1093/cercor/bhu012 (2015).

37 Ng, K. K., Lo, J. C., Lim, J. K., Chee, M. W. & Zhou, J. Reduced functional segregation between the default mode network and the executive control network in healthy older adults: A longitudinal study. *Neuroimage*. **133**, 321-330, doi:10.1016/j.neuroimage.2016.03.029 (2016).

38 Chua, K. Y., Lin, X., Lim, W. S. & Koh, W. P. Gender and physical frailty modify the association between abdominal obesity and functional disability in older adults. *Aging Clin Exp Res*. **34**, 2013-2021, doi:10.1007/s40520-022-02167-z (2022).

39 Caspi, Y. *et al.* Changes in the intracranial volume from early adulthood to the sixth decade of life: A longitudinal study. *Neuroimage*. **220**, 116842, doi:10.1016/j.neuroimage.2020.116842 (2020).

40 Du, W., Ding, C., Jiang, J. & Han, Y. Women Exhibit Lower Global Left Frontal Cortex Connectivity Among Cognitively Unimpaired Elderly Individuals: A Pilot Study from SILCODE. *Journal of Alzheimer's disease : JAD*. **83**, 653-663, doi:10.3233/JAD-210376 (2021).

41 Geerligs, L., Maurits, N. M., Renken, R. J. & Lorist, M. M. Reduced specificity of functional connectivity in the aging brain during task performance. *Human brain mapping*. **35**, 319-330, doi:10.1002/hbm.22175 (2014).

42 Hanggi, J. *et al.* Strength of Structural and Functional Frontostriatal Connectivity Predicts Self-Control in the Healthy Elderly. *Frontiers in Aging Neuroscience*. **8**, 307, doi:10.3389/fnagi.2016.00307 (2016).

43 Lin, C. *et al.* The effect of amyloid deposition on longitudinal resting-state functional connectivity in cognitively normal older adults. *Alzheimers Res Ther*. **12**, 7, doi:10.1186/s13195-019-0573-1 (2020).

44 Zonneveld, H. I. *et al.* Patterns of functional connectivity in an aging population: The Rotterdam Study. *Neuroimage*. **189**, 432-444, doi:10.1016/j.neuroimage.2019.01.041 (2019).

45 Rinne, P. *et al.* Motor dexterity and strength depend upon integrity of the attention-control system. *Proceedings of the National Academy of Sciences of the United States of America*. **115**, E536-E545, doi:10.1073/pnas.1715617115 (2018).

46 Chen, J. *et al.* Shared and unique brain network features predict cognitive, personality, and mental health scores in the ABCD study. *Nature communications*. **13**, 2217, doi:10.1038/s41467-022-29766-8 (2022).

47 He, T. *et al.* Deep neural networks and kernel regression achieve comparable accuracies for functional connectivity prediction of behavior and demographics. *Neuroimage*. **206**, 116276, doi:10.1016/j.neuroimage.2019.116276 (2020).

48 Ooi, L. Q. R. *et al.* Comparison of individualized behavioral predictions across anatomical, diffusion and functional connectivity MRI. *Neuroimage*. **263**, 119636, doi:10.1016/j.neuroimage.2022.119636 (2022).

49 Salthouse, T. A. Aging and measures of processing speed. *Biol Psychol*. **54**, 35-54, doi:10.1016/s0301-0511(00)00052-1 (2000).

50 Salthouse, T. A. & Ferrer-Caja, E. What needs to be explained to account for age-related effects on multiple cognitive variables? *Psychol Aging*. **18**, 91-110, doi:10.1037/0882-7974.18.1.91 (2003).

51 Verhaeghen, P. & Salthouse, T. A. Meta-analyses of age-cognition relations in adulthood: estimates of linear and nonlinear age effects and structural models. *Psychol Bull*. **122**, 231-249, doi:10.1037/0033-2909.122.3.231 (1997).

52 Jiang, R. *et al.* Associations between grip strength, brain structure, and mental health in > 40,000 participants from the UK Biobank. *BMC Med*. **20**, 286, doi:10.1186/s12916-022-02490-2 (2022).

53 Firth, J. *et al.* Association Between Muscular Strength and Cognition in People With Major Depression or Bipolar Disorder and Healthy Controls. *JAMA Psychiatry*. **75**, 740-746, doi:10.1001/jamapsychiatry.2018.0503 (2018).

54 Mather, M. *et al.* Isometric exercise facilitates attention to salient events in women via the noradrenergic system. *Neuroimage*. **210**, 116560, doi:10.1016/j.neuroimage.2020.116560 (2020).

55 Bachman, S. L., Attanti, S. & Mather, M. Isometric handgrip exercise speeds working memory responses in younger and older adults. *Psychol Aging*. **38**, 305-322, doi:10.1037/pag0000728 (2023).

56 Washio, T. *et al.* Effects of acute interval handgrip exercise on cognitive performance. *Physiol Behav*. **232**, 113327, doi:10.1016/j.physbeh.2021.113327 (2021).

57 Ruiz-Rizzo, A. L. *et al.* Decreased cingulo-opercular network functional connectivity mediates the impact of aging on visual processing speed. *Neurobiology of aging*. **73**, 50-60, doi:10.1016/j.neurobiolaging.2018.09.014 (2019).

58 Bundesen, C., Vangkilde, S. & Petersen, A. Recent developments in a computational theory of visual attention (TVA). *Vision research*. **116**, 210-218, doi:10.1016/j.visres.2014.11.005 (2015).

59 Penning, M. D. *et al.* Alertness Training Increases Visual Processing Speed in Healthy Older Adults. *Psychol Sci*. **32**, 340-353, doi:10.1177/0956797620965520 (2021).

60 Twait, E., Farah, R. & Horowitz-Kraus, T. Decreased functional connectivity of the salience network during narrative comprehension in children with reading difficulties: An fMRI study. *NeuroImage. Clinical*. **20**, 987-992, doi:10.1016/j.nicl.2018.10.006 (2018).

61 Schiwy, L. C. *et al.* Aberrant functional connectivity within the salience network is related to cognitive deficits and disorganization in psychosis. *Schizophr Res*. **246**, 103-111, doi:10.1016/j.schres.2022.06.008 (2022).

62 Schaie, K. W. *Intellectual Development in Adulthood: The Seattle Longitudinal Study*. (Cambridge University Press, 1996).

63 Zelinski, E. M. & Burnight, K. P. Sixteen-year longitudinal and time lag changes in memory and cognition in older adults. *Psychol Aging*. **12**, 503-513 (1997).

64 Shen, H. *et al.* Changes in functional connectivity dynamics associated with vigilance network in taxi drivers. *Neuroimage*. **124**, 367-378, doi:10.1016/j.neuroimage.2015.09.010 (2016).

65 Breckel, T. P., Giessing, C. & Thiel, C. M. Impact of brain networks involved in vigilance on processing irrelevant visual motion. *Neuroimage*. **55**, 1754-1762, doi:10.1016/j.neuroimage.2011.01.025 (2011).

66 Petersen, S. E. & Posner, M. I. The attention system of the human brain: 20 years after. *Annu Rev Neurosci*. **35**, 73-89, doi:10.1146/annurev-neuro-062111-150525 (2012).

67 Oken, B. S., Salinsky, M. C. & Elsas, S. M. Vigilance, alertness, or sustained attention: physiological basis and measurement. *Clin Neurophysiol*. **117**, 1885-1901, doi:10.1016/j.clinph.2006.01.017 (2006).

68 Schneider, M. *et al.* Spontaneous pupil dilations during the resting state are associated with activation of the salience network. *Neuroimage*. **139**, 189-201, doi:10.1016/j.neuroimage.2016.06.011 (2016).

69 Sadaghiani, S. & D'Esposito, M. Functional Characterization of the Cingulo-Opercular Network in the Maintenance of Tonic Alertness. *Cerebral cortex*. **25**, 2763-2773, doi:10.1093/cercor/bhu072 (2015).

70 Posner, M. I. & Petersen, S. E. The attention system of the human brain. *Annu Rev Neurosci*. **13**, 25-42, doi:10.1146/annurev.ne.13.030190.000325 (1990).

71 Seidler, R. *et al.* Associations between age, motor function, and resting state sensorimotor network connectivity in healthy older adults. *Neuroimage*. **108**, 47-59, doi:10.1016/j.neuroimage.2014.12.023 (2015).

72 Yarkoni, T., Poldrack, R. A., Nichols, T. E., Van Essen, D. C. & Wager, T. D. Large-scale automated synthesis of human functional neuroimaging data. *Nature methods*. **8**, 665-670, doi:10.1038/nmeth.1635 (2011).

73 Poldrack, R. A. *et al.* Discovering relations between mind, brain, and mental disorders using topic mapping. *PLoS computational biology*. **8**, e1002707, doi:10.1371/journal.pcbi.1002707 (2012).

74 Salo, T. *et al.* NiMARE: Neuroimaging Meta-Analysis Research Environment. *NeuroLibre Reproducible Preprint Server*. **1**, 7, doi:10.55458/neurolibre.00007 (2022).

75 neurostuff/NiMARE: 0.0.12 v. 0.0.12 (Zenodo, 2022).

76 Wordcloud: Word Clouds. (2018).

77 Fukumori, N. *et al.* Association between hand-grip strength and depressive symptoms: Locomotive Syndrome and Health Outcomes in Aizu Cohort Study (LOHAS). *Age Ageing*. **44**, 592-598, doi:10.1093/ageing/afv013 (2015).

78 Gu, Y. *et al.* Grip strength and depressive symptoms in a large-scale adult population: The TCLSIH cohort study. *J Affect Disord*. **279**, 222-228, doi:10.1016/j.jad.2020.08.023 (2021).

79 Jeong, S. M. *et al.* Association among handgrip strength, body mass index and decline in cognitive function among the elderly women. *BMC Geriatr*. **18**, 225, doi:10.1186/s12877-018-0918-9 (2018).

80 Lin, M. H. *et al.* Association between grip strength and anthropometric characteristics in the community-dwelling elderly population in Taiwan. *PloS one*. **16**, e0260763, doi:10.1371/journal.pone.0260763 (2021).

81 Palacio-Aguero, A., Diaz-Torrente, X. & Quintiliano Scarpelli Dourado, D. Relative handgrip strength, nutritional status and abdominal obesity in Chilean adolescents. *PloS one*. **15**, e0234316, doi:10.1371/journal.pone.0234316 (2020).

82 Shozi, S., Monyeki, M. A., Moss, S. J. & Pienaar, C. Relationships between physical activity, body mass index, waist circumference and handgrip strength amongst adults from the North West province, South Africa: The PURE study. *Afr J Prim Health Care Fam Med*. **14**, e1-e11, doi:10.4102/phcfm.v14i1.3206 (2022).

Reviewers' comments:

Reviewer #1 (Remarks to the Author):

I commend the authors for such a careful and thorough response and revision of the manuscript. I think everything looks quite good; I have just one last comment:

In the manuscript, the authors state that they correct for TIV (total intracranial volume). They state in the R2R that brain volume changes with age, hence they covaried for TIV. But, TIV includes CSF, not just brain volume, and typically does not change with age. If the authors corrected the analyses for whole brain volume, they need to amend the text to state this. It seems like a minor point, but covarying for TIV versus total brain volume would have rather different effects on the outcomes.

Reviewer #2 (Remarks to the Author):

The authors of the manuscript "Higher handgrip strength is linked to higher salience/ventral attention functional network segregation in older adults" have revised their manuscript and addressed most of my concerns. The manuscript is improved.

However, the authors have not addressed my main concern which is that the three main variables "handgrip strength", "cognition", and "functional connectivity" are not analysed jointly and therefore the analyses do not match the hypotheses and motivation for the study. The authors repeatedly state things like "Handgrip strength is linked to cognition in older adults, and this may be subserved by shared age-related changes in brain function and structure", "The interplay among handgrip strength, brain functional connectivity, and cognitive function remains poorly elucidated.", "Understanding the neural mechanisms underlying the handgrip strength-cognition relationship".

In order to test whether brain measures share variance with the handgrip-cognition association, I strongly recommend to add a linear regression to the existing series in which the analysis between handgrip strength and cognition is repeated and also includes salience/ventral attention intra-network functional connectivity as a predictor. Then, the coefficient for handgrip strength as a predictor for cognition should be compared between this model and the model you already have (i.e. how functional connectivity statistically explains the association between grip strength and cognition. Fully, partial, not at all?).

This does not imply making a new decision about dependent and independent variables so I do not understand the author response here. I agree that causality cannot be implied for this type of analysis but you already make predictions about which variables are your dependent and independent variables in your regressions based on (sensible) theory. The additional analysis will just be an extension of this that aligns with your hypotheses - my apologies if my comment was unclear in the first round.

There are many examples of this type of analysis. Consider for example https://bmcmedicine.biomedcentral.com/articles/10.1186/s12916-022-02490-2 Figure 5.

Responses to reviewers:

We would like to thank the editors and both reviewers for their insightful and constructive comments. We have conducted new analyses and revised the manuscript to address the additional comments raised by the reviewers. We believe that the revised manuscript has been further improved and the questions from the reviewers have been well addressed.

Detailed point-to-point responses to reviewers are as follows:

Reviewer #1:
1. I commend the authors for such a careful and thorough response and revision of the manuscript. I think everything looks quite good; I have just one last comment:

In the manuscript, the authors state that they correct for TIV (total intracranial volume). They state in the R2R that brain volume changes with age, hence they covaried for TIV. But, TIV includes CSF, not just brain volume, and typically does not change with age. If the authors corrected the analyses for whole brain volume, they need to amend the text to state this. It seems like a minor point, but covarying for TIV versus total brain volume would have rather different effects on the outcomes.

Response: We thank the reviewer for the comment. To clarify, we have controlled for total intracranial volume (TIV) (comprising white matter, grey matter and cerebrospinal fluid volumes) in the study, which itself has been demonstrated to decline with age in older adults[1], correlate with intrinsic brain activity in healthy young adults[2], and has been included as a nuisance covariate in previous functional connectivity studies in older adults[3-6]. Nevertheless, we acknowledge the reviewer's point that covarying for TIV versus total brain volume might have different effects on the outcomes. Given that our study examined the functional connectivity of grey matter regions-of-interest (ROIs), we have repeated the analyses specifically controlling for total grey matter volume following previous work[6-10]. All of our key findings remained after controlling for total grey matter volume (Supplementary Tables 4-6, 10), indicating the robustness of our findings. We have included the new analyses controlling for total grey matter volume in the Methods (pp. 14, lines 317-320), Results (pp. 16 lines 363-366, pp. 17 lines 390-392) and Supplementary Tables 2, 4-6, 10. The revised Methods and Supplementary Tables are provided below (amendments are in bold):

<u>Methods (pp. 14, lines 317-320)</u>
"**Lastly, to ensure that functional connectivity associations with handgrip strength and cognition were not influenced by variations in total grey matter volumes, we repeated the functional connectivity analyses with total grey matter volumes (instead of total intracranial volumes) included as a covariate.**"

Results (pp. 16, lines 363-366)

"All these findings remained after controlling for SM-MMSE scores, Geriatric Depression Scale scores, body mass index, waist-hip ratio, time interval between MRI scan and handgrip strength assessment, **or total grey matter volumes (instead of total intracranial volumes)** (Supplementary Tables 4-6)."

Results (pp. 17, lines 390-392)

"These findings remained after controlling for SM-MMSE scores, Geriatric Depression Scale scores, body mass index, waist-hip ratio, **or total grey matter volumes (instead of total intracranial volumes)** (Supplementary Table 10)."

Supplementary Table 2. List of validation analyses performed

Analysis	Dataset	Nuisance covariates included
1	Original dataset (n = 148)	Age, sex, education, TIV and SM-MMSE
2	Original dataset (n = 148)	Age, sex, education, TIV and waist-hip ratio
3	Original dataset, excluding 2 subjects with missing body mass index values (n = 146)	Age, sex, education, TIV and body mass index
4	Original dataset (n = 148)	Age, sex, education, TIV and GDS scores
5[#]	Original dataset (n = 148)	Age, sex, education, TIV and time interval between MRI scan and handgrip strength measurement
**6***	**Original dataset (n = 148)**	**Age, sex, education and total grey matter volumes**

[#]For analysis 5, only associations between handgrip strength and functional connectivity measures were examined.
***For analysis 6, only associations between handgrip strength and functional connectivity measures, and associations between functional connectivity measures and cognitive performance were examined.**
Abbreviations: TIV, total intracranial volumes; SM-MMSE, Singapore-modified version of the mini-mental state examination; GDS, geriatric depression scale.

Supplementary Table 4: Validation analyses: associations between handgrip strength and global and network-level system segregation

Measure	Coefficient	SE	*t*	Uncorr *p*	FDR-adj *p*
**Analysis 1: Analysis additionally controlling for SM-MMSE (n = 148)**					
Global segregation	$1.80e^{-3}$	$8.86e^{-4}$	2.03	0.0439*	0.0439*
Network segregation					
Default	$2.41e^{-3}$	$1.53e^{-3}$	1.57	0.1187	0.2916
Control	$6.78e^{-4}$	$1.65e^{-3}$	0.41	0.6815	0.7667
Limbic	$1.25e^{-3}$	$1.83e^{-3}$	0.68	0.4971	0.6541
Salience/ventral attention	$4.89e^{-3}$	$1.49e^{-3}$	3.29	0.0013*	0.0115*
Dorsal attention	$1.27e^{-3}$	$1.92e^{-3}$	0.66	0.5088	0.6541
Somatomotor	$2.89e^{-3}$	$1.70e^{-3}$	1.70	0.0909	0.2916
Visual	$2.89e^{-4}$	$1.09e^{-3}$	0.27	0.7905	0.7905
Temporoparietal	$5.26e^{-3}$	$3.45e^{-3}$	1.52	0.1296	0.2916
Subcortical	$1.25e^{-3}$	$1.24e^{-3}$	1.01	0.3137	0.5646
**Analysis 2: Analysis additionally controlling for waist-hip ratio (n = 148)**					
Global segregation	$1.68e^{-3}$	$8.90e^{-4}$	1.89	0.0607	0.0607
Network segregation					
Default	$2.34e^{-3}$	$1.54e^{-3}$	1.52	0.1298	0.2920
Control	$8.55e^{-4}$	$1.65e^{-3}$	0.52	0.6041	0.6796

Limbic	1.22e^{-3}	1.83e^{-3}	0.67	0.5056	0.6796
Salience/ventral attention	5.01e^{-3}	1.48e^{-3}	3.37	0.0010*	0.0086*
Dorsal attention	1.11e^{-3}	1.93e^{-3}	0.57	0.5665	0.6796
Somatomotor	2.84e^{-3}	1.70e^{-3}	1.68	0.0960	0.2920
Visual	1.44e^{-4}	1.08e^{-3}	0.13	0.8946	0.8946
Temporoparietal	5.46e^{-3}	3.44e^{-3}	1.59	0.1145	0.2920
Subcortical	9.79e^{-4}	1.24e^{-3}	0.79	0.4319	0.6796
**Analysis 3: Analysis additionally controlling for body mass index (n = 146)**					
Global segregation	1.68e^{-3}	9.45e^{-4}	1.78	0.0774	0.0774
Network segregation					
Default	2.00e^{-3}	1.64e^{-3}	1.22	0.2254	0.4550
Control	7.16e^{-4}	1.73e^{-3}	0.41	0.6803	0.7654
Limbic	1.26e^{-3}	1.95e^{-3}	0.65	0.5183	0.6664
Salience/ventral attention	4.88e^{-3}	1.56e^{-3}	3.12	0.0022*	0.0199*
Dorsal attention	2.31e^{-3}	2.01e^{-3}	1.15	0.2528	0.4550
Somatomotor	2.77e^{-3}	1.80e^{-3}	1.54	0.1262	0.3787
Visual	2.84e^{-4}	1.15e^{-3}	0.25	0.8062	0.8062
Temporoparietal	6.37e^{-3}	3.48e^{-3}	1.83	0.0693	0.3121
Subcortical	1.13e^{-3}	1.32e^{-3}	0.86	0.3937	0.5906
**Analysis 4: Analysis additionally controlling for GDS scores (n = 148)**					
Global segregation	1.66e^{-3}	8.80e^{-4}	1.89	0.0613	0.0613
Network segregation					
Default	2.10e^{-3}	1.52e^{-3}	1.38	0.1704	0.3835
Control	6.93e^{-4}	1.64e^{-3}	0.42	0.6730	0.7571
Limbic	1.43e^{-3}	1.82e^{-3}	0.78	0.4343	0.6515
Salience/ventral attention	4.87e^{-3}	1.48e^{-3}	3.30	0.0012*	0.0112*
Dorsal attention	1.04e^{-3}	1.92e^{-3}	0.54	0.5897	0.7571
Somatomotor	2.83e^{-3}	1.68e^{-3}	1.69	0.0939	0.3726
Visual	2.21e^{-4}	1.07e^{-3}	0.21	0.8372	0.8372
Temporoparietal	5.22e^{-3}	3.38e^{-3}	1.55	0.1242	0.3726
Subcortical	1.04e^{-3}	1.23e^{-3}	0.84	0.4007	0.6515
**Analysis 5: Analysis additionally controlling for time interval between MRI scan and handgrip strength measurement (n = 148)**					
Global segregation	1.52e^{-3}	8.99e^{-4}	1.69	0.0934	0.0934
Network segregation					
Default	2.49e^{-3}	1.56e^{-3}	1.60	0.1127	0.3203
Control	-4.20e^{-5}	1.64e^{-3}	-0.03	0.9796	0.9796
Limbic	1.36e^{-3}	1.86e^{-3}	0.73	0.4675	0.7344
Salience/ventral attention	4.33e^{-3}	1.49e^{-3}	2.91	0.0042*	0.0378*
Dorsal attention	1.10e^{-3}	1.96e^{-3}	0.56	0.5761	0.7406
Somatomotor	3.31e^{-3}	1.71e^{-3}	1.94	0.0549	0.2472
Visual	-4.08e^{-4}	1.06e^{-3}	-0.38	0.7015	0.7892
Temporoparietal	5.16e^{-3}	3.50e^{-3}	1.48	0.1424	0.3203
Subcortical	8.72e^{-4}	1.26e^{-3}	0.69	0.4896	0.7344
**Analysis 6: Analysis controlling for total grey matter volumes instead of TIV (n = 148)**					
**Global segregation**	**1.75e^{-3}**	**9.17e^{-4}**	**1.91**	**0.0580**	**0.0580**
**Network segregation**					
**Default**	**1.85e^{-3}**	**1.59e^{-3}**	**1.17**	**0.2456**	**0.5526**
**Control**	**7.52e^{-4}**	**1.69e^{-3}**	**0.44**	**0.6579**	**0.6653**
**Limbic**	**8.15e^{-4}**	**1.88e^{-3}**	**0.43**	**0.6653**	**0.6653**
**Salience/ventral attention**	**5.04e^{-3}**	**1.54e^{-3}**	**3.26**	**0.0014***	**0.0125***
**Dorsal attention**	**1.02e^{-3}**	**1.98e^{-3}**	**0.51**	**0.6090**	**0.6653**
**Somatomotor**	**2.90e^{-3}**	**1.74e^{-3}**	**1.66**	**0.0988**	**0.4448**
**Visual**	**6.55e^{-4}**	**1.11e^{-3}**	**0.59**	**0.5560**	**0.6653**
**Temporoparietal**	**5.15e^{-3}**	**3.55e^{-3}**	**1.45**	**0.1488**	**0.4464**

**Subcortical**	**$1.26e^{-3}$**	**$1.28e^{-3}$**	**0.99**	**0.3247**	**0.5845**

Multiple comparison correction was performed across the nine network-level segregation measures. * indicates statistically significant effects (p < 0.05). Abbreviations: SE, standard error; Uncorr, uncorrected; FDR-adj, false discovery rate-adjusted; SM-MMSE, Singapore-modified version of the mini-mental state examination; GDS, geriatric depression scale scores; **TIV, total intracranial volumes**.

Supplementary Table 5: Validation analyses: associations between handgrip strength and mean salience/ventral attention inter- and intra-network functional connectivity

Measure	Coefficient	SE	*t*	Uncorr *p*	FDR-adj *p*
**Analysis 1: Analysis additionally controlling for SM-MMSE (n = 148)**					
Intra-network S/VA	$4.21e^{-3}$	$1.47e^{-3}$	2.87	0.0048*	0.0428*
Inter-network					
S/VA – Default	$-3.04e^{-3}$	$1.40e^{-3}$	-2.18	0.0312*	0.0935
S/VA – Control	$-9.52e^{-4}$	$1.07e^{-3}$	-0.89	0.3740	0.4927
S/VA – Limbic	$-8.40e^{-4}$	$1.45e^{-3}$	-0.58	0.5636	0.5636
S/VA – Dorsal attention	$1.20e^{-3}$	$1.37e^{-3}$	0.87	0.3832	0.4927
S/VA – Somatomotor	$3.34e^{-3}$	$1.44e^{-3}$	2.32	0.0215*	0.0935
S/VA – Visual	$2.72e^{-3}$	$1.81e^{-3}$	1.50	0.1347	0.3031
S/VA – Temporoparietal	$1.32e^{-3}$	$1.94e^{-3}$	0.68	0.4991	0.5615
S/VA – Subcortical	$9.88e^{-4}$	$9.31e^{-4}$	1.06	0.2902	0.4927
**Analysis 2: Analysis additionally controlling for waist-hip ratio (n = 148)**					
Intra-network S/VA	$4.47e^{-3}$	$1.46e^{-3}$	3.05	0.0027*	0.0243*
Inter-network					
S/VA – Default	$-3.14e^{-3}$	$1.39e^{-3}$	-2.26	0.0253*	0.0758
S/VA – Control	$-9.73e^{-4}$	$1.07e^{-3}$	-0.91	0.3632	0.5067
S/VA – Limbic	$-8.59e^{-4}$	$1.45e^{-3}$	-0.59	0.5550	0.5550
S/VA – Dorsal attention	$1.57e^{-3}$	$1.33e^{-3}$	1.18	0.2414	0.4345
S/VA – Somatomotor	$3.45e^{-3}$	$1.44e^{-3}$	2.40	0.0176*	0.0758
S/VA – Visual	$2.66e^{-3}$	$1.81e^{-3}$	1.46	0.1452	0.3266
S/VA – Temporoparietal	$1.47e^{-3}$	$1.94e^{-3}$	0.76	0.4504	0.5067
S/VA – Subcortical	$7.69e^{-4}$	$9.23e^{-4}$	0.83	0.4062	0.5067
**Analysis 3: Analysis additionally controlling for body mass index (n = 146)**					
Intra-network S/VA	$4.37e^{-3}$	$1.56e^{-3}$	2.81	0.0057*	0.0510
Inter-network					
S/VA – Default	$-2.48e^{-3}$	$1.47e^{-3}$	-1.68	0.0946	0.2839
S/VA – Control	$-3.66e^{-4}$	$1.13e^{-3}$	-0.32	0.7475	0.7475
S/VA – Limbic	$-6.59e^{-4}$	$1.54e^{-3}$	-0.43	0.6683	0.7475
S/VA – Dorsal attention	$1.20e^{-3}$	$1.42e^{-3}$	0.84	0.4018	0.6925
S/VA – Somatomotor	$3.06e^{-3}$	$1.52e^{-3}$	2.01	0.0461*	0.2075
S/VA – Visual	$2.07e^{-3}$	$1.93e^{-3}$	1.07	0.2845	0.6401
S/VA – Temporoparietal	$1.15e^{-3}$	$2.04e^{-3}$	0.56	0.5743	0.7384
S/VA – Subcortical	$7.18e^{-4}$	$9.73e^{-4}$	0.74	0.4617	0.6925
**Analysis 4: Analysis additionally controlling for GDS scores (n = 148)**					
Intra-network S/VA	$4.30e^{-3}$	$1.46e^{-3}$	2.94	0.0038*	0.0345*
Inter-network					
S/VA – Default	$-2.74e^{-3}$	$1.39e^{-3}$	-1.98	0.0499*	0.1496
S/VA – Control	$-7.28e^{-4}$	$1.07e^{-3}$	-0.68	0.4961	0.5425
S/VA – Limbic	$-8.81e^{-4}$	$1.44e^{-3}$	-0.61	0.5425	0.5425
S/VA – Dorsal attention	$1.08e^{-3}$	$1.36e^{-3}$	0.80	0.4261	0.5425
S/VA – Somatomotor	$3.32e^{-3}$	$1.43e^{-3}$	2.33	0.0214*	0.0963
S/VA – Visual	$2.38e^{-3}$	$1.81e^{-3}$	1.31	0.1910	0.4298
S/VA – Temporoparietal	$1.33e^{-3}$	$1.91e^{-3}$	0.70	0.4879	0.5425
S/VA – Subcortical	$9.51e^{-4}$	$9.26e^{-4}$	1.03	0.3061	0.5425

<u>Analysis 5: Analysis additionally controlling for time interval between MRI scan and handgrip strength measurement (n = 148)</u>					
Intra-network S/VA	$3.97e^{-3}$	$1.48e^{-3}$	2.67	0.0084*	0.0753
Inter-network					
S/VA – Default	$-2.65e^{-3}$	$1.43e^{-3}$	-1.85	0.0658	0.1975
S/VA – Control	$-7.25e^{-4}$	$1.09e^{-3}$	-0.66	0.5081	0.5716
S/VA – Limbic	$-1.21e^{-3}$	$1.47e^{-3}$	-0.82	0.4117	0.5294
S/VA – Dorsal attention	$1.19e^{-3}$	$1.39e^{-3}$	0.85	0.3943	0.5294
S/VA – Somatomotor	$3.05e^{-3}$	$1.45e^{-3}$	2.10	0.0372*	0.1674
S/VA – Visual	$2.59e^{-3}$	$1.85e^{-3}$	1.40	0.1623	0.3653
S/VA – Temporoparietal	$7.60e^{-4}$	$1.96e^{-3}$	0.39	0.6986	0.6986
S/VA – Subcortical	$1.11e^{-3}$	$9.42e^{-4}$	1.18	0.2399	0.4319
<u>Analysis 6: Analysis controlling for total grey matter volumes instead of TIV (n = 148)</u>					
**Intra-network S/VA**	**$4.19e^{-3}$**	**$1.53e^{-3}$**	**2.73**	**0.0071***	**0.0643**
**Inter-network**					
**S/VA – Default**	**$-2.47e^{-3}$**	**$1.46e^{-3}$**	**-1.69**	**0.0926**	**0.2778**
**S/VA – Control**	**$-8.80e^{-4}$**	**$1.11e^{-3}$**	**-0.79**	**0.4283**	**0.6382**
**S/VA – Limbic**	**$-8.65e^{-4}$**	**$1.50e^{-3}$**	**-0.58**	**0.5652**	**0.6382**
**S/VA – Dorsal attention**	**$6.71e^{-4}$**	**$1.42e^{-3}$**	**0.47**	**0.6382**	**0.6382**
**S/VA – Somatomotor**	**$3.62e^{-3}$**	**$1.49e^{-3}$**	**2.43**	**0.0162***	**0.0728**
**S/VA – Visual**	**$2.44e^{-3}$**	**$1.88e^{-3}$**	**1.30**	**0.1955**	**0.4398**
**S/VA – Temporoparietal**	**$1.00e^{-3}$**	**$2.00e^{-3}$**	**0.50**	**0.6155**	**0.6382**
**S/VA – Subcortical**	**$9.99e^{-4}$**	**$9.57e^{-4}$**	**1.04**	**0.2987**	**0.5376**

Multiple comparison correction was performed across the nine salience/ventral attention inter- and intra-network functional connectivity measures. * indicates statistically significant effects (p < 0.05). Abbreviations: SE, standard error; Uncorr, uncorrected; FDR-adj, false discovery rate-adjusted; S/VA, salience/ventral attention; SM-MMSE, Singapore-modified version of the mini-mental state examination; GDS, geriatric depression scale scores; **TIV, total intracranial volumes**.

Supplementary Table 6: Validation analyses: significant associations between handgrip strength and intra-network salience/ventral attention functional connections

Measure	Coefficient	SE	*t*	Uncorr *p*	FDR-adj *p*
<u>Analysis 1: Analysis additionally controlling for SM-MMSE (n = 148)</u>					
Right AI – Left PI/FO	$1.54e^{-2}$	$3.86e^{-3}$	3.99	0.0001*	0.0177*
Right AI – Right MCC/MPC	$1.53e^{-2}$	$3.92e^{-3}$	3.89	0.0002*	0.0177*
<u>Analysis 2: Analysis additionally controlling for waist-hip ratio (n = 148)</u>					
Right AI – Left PI/FO	$1.62e^{-2}$	$3.81e^{-3}$	4.24	<0.0001*	0.0046*
Right AI – Right MCC/MPC	$1.66e^{-2}$	$3.87e^{-3}$	4.30	<0.0001*	0.0046*
<u>Analysis 3: Analysis additionally controlling for body mass index (n = 146)</u>					
Right AI – Left PI/FO	$1.57e^{-2}$	$4.09e^{-3}$	3.84	0.0002*	0.0191*
Right AI – Right MCC/MPC	$1.58e^{-2}$	$4.11e^{-3}$	3.84	0.0002*	0.0191*
<u>Analysis 4: Analysis additionally controlling for GDS scores (n = 148)</u>					
Right AI – Left PI/FO	$1.53e^{-2}$	$3.83e^{-3}$	4.00	0.0001*	0.0126*
Right AI – Right MCC/MPC	$1.55e^{-2}$	$3.90e^{-3}$	3.98	0.0001*	0.0126*
<u>Analysis 5: Analysis additionally controlling for time interval between MRI scan and handgrip strength measurement (n = 148)</u>					
Right AI – Left PI/FO	$1.44e^{-2}$	$3.89e^{-3}$	3.70	0.0003*	0.0358*
Right AI – Right MCC/MPC	$1.51e^{-2}$	$3.98e^{-3}$	3.79	0.0002*	0.0358*
<u>Analysis 6: Analysis controlling for total grey matter volumes instead of TIV (n = 148)</u>					
**Right AI – Left PI/FO**	**$1.43e^{-2}$**	**$3.96e^{-3}$**	**3.62**	**0.0004***	**0.0468***
**Right AI – Right MCC/MPC**	**$1.74e^{-2}$**	**$4.07e^{-3}$**	**4.27**	**<0.0001***	**0.0084***

Multiple comparison correction was performed across 231 pairs of regional connections within the salience/ventral attention network. * indicates statistically significant effects ($p < 0.05$). Abbreviations: SE, standard error; Uncorr, uncorrected; FDR-adj, false discovery rate-adjusted; SM-MMSE, Singapore-modified version of the mini-mental state examination; GDS, geriatric depression scale scores; AI, anterior insula; PI, posterior insula; FO, frontal operculum; MCC, midcingulate cortex; MPC, medial parietal cortex; **TIV, total intracranial volumes**.

Supplementary Table 10: Validation analyses: associations between cognitive performance and functional connectivity measures

FC measure	Cognitive measure	Coefficient	SE	*t*	Uncorr *p*	FDR-adj *p*
**Analysis 1: Analysis additionally controlling for SM-MMSE (n = 148)**						
S/VA segregation	Global	8.54	4.07	2.10	0.0376*	0.0752
	Processing speed	16.29	5.66	2.88	0.0046*	0.0184*
	Attention	2.97	5.39	0.55	0.5829	0.5829
	Executive function	6.36	4.97	1.28	0.2025	0.2700
Intra-network S/VA	Global	10.76	4.12	2.61	0.0100*	0.0199*
	Processing speed	18.03	5.74	3.14	0.0021*	0.0083*
	Attention	5.76	5.49	1.05	0.2960	0.2960
	Executive function	8.49	5.05	1.68	0.0948	0.1264
Right AI – Left PI/FO	Global	4.01	1.53	2.62	0.0098*	0.0196*
	Processing speed	6.88	2.13	3.23	0.0015*	0.0062*
	Attention	2.29	2.04	1.12	0.2638	0.2638
	Executive function	2.85	1.88	1.52	0.1310	0.1746
Right AI – Right MCC/MPC	Global	3.35	1.52	2.21	0.0287*	0.0575
	Processing speed	5.09	2.13	2.39	0.0184*	0.0575
	Attention	2.21	2.01	1.10	0.2734	0.2734
	Executive function	2.76	1.85	1.49	0.1378	0.1837
**Analysis 2: Analysis additionally controlling for waist-hip ratio (n = 148)**						
S/VA segregation	Global	9.03	4.30	2.10	0.0374*	0.0934
	Processing speed	17.15	5.85	2.93	0.0040*	0.0198*
	Attention	3.41	5.57	0.61	0.5415	0.6769
	Executive function	6.54	5.03	1.30	0.1950	0.3251
	SM-MMSE	0.76	1.97	0.39	0.6997	0.6997
Intra-network S/VA	Global	11.70	4.35	2.69	0.0080*	0.0200*
	Processing speed	19.84	5.93	3.35	0.0010*	0.0052*
	Attention	6.55	5.67	1.15	0.2501	0.3126
	Executive function	8.71	5.11	1.70	0.0905	0.1508
	SM-MMSE	1.48	2.01	0.74	0.4630	0.4630
Right AI – Left PI/FO	Global	4.17	1.63	2.56	0.0116*	0.0289*
	Processing speed	7.53	2.21	3.41	0.0009*	0.0043*
	Attention	2.32	2.12	1.10	0.2747	0.3434
	Executive function	2.65	1.92	1.38	0.1692	0.2821
	SM-MMSE	0.28	0.75	0.37	0.7137	0.7137
Right AI – Right MCC/MPC	Global	3.94	1.61	2.46	0.0152*	0.0380*
	Processing speed	6.36	2.20	2.89	0.0045*	0.0223*
	Attention	2.66	2.08	1.28	0.2028	0.2115
	Executive function	2.81	1.88	1.49	0.1378	0.2115
	SM-MMSE	0.92	0.74	1.26	0.2115	0.2115
**Analysis 3: Analysis additionally controlling for body mass index (n = 146)**						
S/VA segregation	Global	9.31	4.37	2.13	0.0350*	0.0876
	Processing speed	17.48	5.92	2.96	0.0037*	0.0183*
	Attention	4.06	5.70	0.71	0.4778	0.5972
	Executive function	6.40	5.19	1.23	0.2198	0.3664
	SM-MMSE	0.45	1.99	0.23	0.8222	0.8222
Intra-network S/VA	Global	11.70	4.39	2.67	0.0085*	0.0214*
	Processing speed	18.84	5.96	3.16	0.0019*	0.0096*

	Attention	7.29	5.74	1.27	0.2060	0.2575
	Executive function	8.96	5.22	1.72	0.0884	0.1473
	SM-MMSE	1.09	2.01	0.54	0.5894	0.5894
Right AI – Left PI/FO	Global	4.23	1.63	2.59	0.0107*	0.0268*
	Processing speed	7.19	2.21	3.25	0.0015*	0.0073*
	Attention	2.62	2.14	1.22	0.2229	0.2786
	Executive function	2.88	1.95	1.48	0.1419	0.2365
	SM-MMSE	0.23	0.75	0.31	0.7551	0.7551
Right AI – Right MCC/MPC	Global	4.24	1.63	2.61	0.0101*	0.0253*
	Processing speed	6.43	2.22	2.90	0.0043*	0.0217*
	Attention	3.06	2.12	1.44	0.1511	0.1850
	Executive function	3.22	1.93	1.66	0.0986	0.1643
	SM-MMSE	0.98	0.74	1.33	0.1850	0.1850
Analysis 4: Analysis additionally controlling for GDS scores (n = 148)						
S/VA segregation	Global	8.80	4.29	2.05	0.0420*	0.1051
	Processing speed	16.38	5.85	2.80	0.0058*	0.0291*
	Attention	3.48	5.59	0.62	0.5346	0.6682
	Executive function	6.54	5.08	1.29	0.2002	0.3336
	SM-MMSE	0.65	1.97	0.33	0.7420	0.7420
Intra-network S/VA	Global	11.88	4.32	2.75	0.0067*	0.0167*
	Processing speed	19.42	5.89	3.30	0.0012*	0.0062*
	Attention	6.85	5.67	1.21	0.2289	0.2861
	Executive function	9.38	5.14	1.82	0.0702	0.1171
	SM-MMSE	1.52	2.00	0.76	0.4488	0.4488
Right AI – Left PI/FO	Global	4.11	1.61	2.55	0.0120*	0.0300*
	Processing speed	6.92	2.20	3.14	0.0020*	0.0102*
	Attention	2.48	2.11	1.17	0.2428	0.3034
	Executive function	2.92	1.92	1.52	0.1303	0.2171
	SM-MMSE	0.24	0.75	0.32	0.7485	0.7485
Right AI – Right MCC/MPC	Global	3.84	1.59	2.42	0.0167*	0.0419*
	Processing speed	5.55	2.19	2.54	0.0123*	0.0419*
	Attention	2.84	2.07	1.37	0.1714	0.2143
	Executive function	3.14	1.88	1.67	0.0981	0.1635
	SM-MMSE	0.86	0.73	1.18	0.2397	0.2397
Analysis 5: Analysis controlling for total grey matter volumes instead of TIV (n = 148)						
**S/VA segregation**	**Global**	**8.33**	**4.21**	**1.98**	**0.0496***	**0.1240**
	**Processing speed**	**15.94**	**5.80**	**2.75**	**0.0068***	**0.0339***
	**Attention**	**3.04**	**5.48**	**0.55**	**0.5799**	**0.6499**
	**Executive function**	**6.02**	**4.98**	**1.21**	**0.2286**	**0.3810**
	**SM-MMSE**	**0.88**	**1.94**	**0.45**	**0.6499**	**0.6499**
**Intra-network S/VA**	**Global**	**10.59**	**4.25**	**2.49**	**0.0139***	**0.0346***
	**Processing speed**	**17.75**	**5.87**	**3.02**	**0.0030***	**0.0148***
	**Attention**	**5.95**	**5.56**	**1.07**	**0.2861**	**0.3577**
	**Executive function**	**8.05**	**5.04**	**1.60**	**0.1126**	**0.1877**
	**SM-MMSE**	**1.55**	**1.97**	**0.79**	**0.4335**	**0.4335**
**Right AI – Left PI/FO**	**Global**	**3.78**	**1.62**	**2.33**	**0.0211***	**0.0528**
	**Processing speed**	**6.75**	**2.23**	**3.02**	**0.0030***	**0.0150***
	**Attention**	**2.09**	**2.12**	**0.99**	**0.3256**	**0.4070**
	**Executive function**	**2.50**	**1.93**	**1.30**	**0.1960**	**0.3266**
	**SM-MMSE**	**0.19**	**0.75**	**0.25**	**0.8057**	**0.8057**
**Right AI – Right MCC/MPC**	**Global**	**3.88**	**1.55**	**2.51**	**0.0132***	**0.0331***
	**Processing speed**	**5.63**	**2.15**	**2.61**	**0.0099***	**0.0331***
	**Attention**	**2.83**	**2.02**	**1.40**	**0.1623**	**0.1623**
	**Executive function**	**3.18**	**1.83**	**1.73**	**0.0855**	**0.1425**
	**SM-MMSE**	**1.02**	**0.71**	**1.42**	**0.1569**	**0.1623**

For each functional connectivity measure, multiple comparison correction was performed across the five cognitive measures. * indicates statistically significant effects ($p < 0.05$). Abbreviations: FC, functional connectivity; SE, standard error; Uncorr, uncorrected; FDR-adj, false discovery rate-adjusted; SM-MMSE, Singapore-modified

version of the mini-mental state examination; GDS, geriatric depression scale scores; S/VA, salience/ventral attention network; R, right; L, left; AI, anterior insula; PI, posterior insula; FO, frontal operculum; MCC, midcingulate cortex; MPC, medial parietal cortex; **TIV, total intracranial volumes**.

Reviewer #2 (Remarks to the Author):

1. The authors of the manuscript "Higher handgrip strength is linked to higher salience/ventral attention functional network segregation in older adults" have revised their manuscript and addressed most of my concerns. The manuscript is improved.

However, the authors have not addressed my main concern which is that the three main variables "handgrip strength", "cognition", and "functional connectivity" are not analysed jointly and therefore the analyses do not match the hypotheses and motivation for the study. The authors repeatedly state things like "Handgrip strength is linked to cognition in older adults, and this may be subserved by shared age-related changes in brain function and structure", "The interplay among handgrip strength, brain functional connectivity, and cognitive function remains poorly elucidated.", "Understanding the neural mechanisms underlying the handgrip strength-cognition relationship".

In order to test whether brain measures share variance with the handgrip-cognition association, I strongly recommend to add a linear regression to the existing series in which the analysis between handgrip strength and cognition is repeated and also includes salience/ventral attention intra-network functional connectivity as a predictor. Then, the coefficient for handgrip strength as a predictor for cognition should be compared between this model and the model you already have (i.e. how functional connectivity statistically explains the association between grip strength and cognition. Fully, partial, not at all?).

This does not imply making a new decision about dependent and independent variables so I do not understand the author response here. I agree that causality cannot be implied for this type of analysis but you already make predictions about which variables are your dependent and independent variables in your regressions based on (sensible) theory. The additional analysis will just be an extension of this that aligns with your hypotheses - my apologies if my comment was unclear in the first round.

There are many examples of this type of analysis. Consider for example https://bmcmedicine.biomedcentral.com/articles/10.1186/s12916-022-02490-2 Figure 5.

Response: We thank the reviewer for the insightful comment. To clarify, due to the cross-sectional nature of our study, we were originally not able to directly establish from the study the dependent and independent variables for the mediation analyses. However, given that several papers have reported a longitudinal effect of handgrip strength on future or rate of cognitive decline[11-14], we agree with the reviewer that such mediation analyses on cross-sectional data would be informative as well.

As recommended, we ran several new mediation analyses with functional connectivity measure as mediator, cognitive measure as dependent variable and handgrip strength as independent variable. Age, sex, education and total intracranial volumes were regressed out from all variables prior to conducting the mediation analyses. We found a significant indirect effect of the right anterior insula – left posterior insula/frontal operculum functional connectivity on the association between handgrip strength and processing speed (proportion of effect size mediated = 27.1%, p = 0.038) (Supplementary Table 11), indicating a partial mediation. Indirect effects of the other salience/ventral attention network functional connectivity measures (i.e., segregation, intra-network functional connectivity, and right anterior insula – right midcingulate/medial parietal cortex connectivity) on the handgrip strength-processing speed association were also observed, however these effects only showed a trend towards significance (proportion mediated = 20.4% – 21.5%, p = 0.063 – 0.102) (Supplementary Table 11). No indirect effects of the functional connectivity measures on the association between handgrip strength and global cognition were found.

In addition, given that grey matter volumes have been associated with handgrip strength[12], we ran the mediation analyses with total grey matter volumes (instead of total intracranial volumes) regressed out from the variables. Similar results were obtained (Supplementary Table 12).

Our findings thus indicate the possibility that common inter-individual differences in salience/ventral attention network functional connectivity could underlie inter-individual differences in both handgrip strength and cognition, although our findings based on cross-sectional data are still preliminary and future longitudinal studies with larger sample sizes are required to confirm these mediation effects (particularly those with smaller effect sizes).

We have revised the Methods (pp. 13-14, lines 287-303), Results (pp. 17-18, lines 393-410), Discussion (pp. 19 lines 433-436, pp. 21-22 lines 493-503) and Supplementary Tables 11-12 accordingly. The revised sections and tables are provided below.

<u>Methods (pp. 13-14, lines 287-303)</u>

“**Mediation analyses examining the mediation effects of functional connectivity on the association between handgrip strength and cognitive performance**

As an exploratory analysis, for functional connectivity measures that showed significant associations with both handgrip strength and cognitive performance, we examined if such functional connectivity measures mediated the relationship between handgrip strength and cognitive performance. Given that several studies have

previously reported a longitudinal effect of handgrip strength on future or rate of cognitive decline[11-14], mediation analyses were conducted with functional connectivity measure as the mediating variable, cognitive score as the dependent variable and handgrip strength as the independent variable. Age, sex, education, and total intracranial volumes were first regressed out from the mediating, independent and dependent variables, and the residuals of these variables were subsequently used in the mediation analyses. Additionally, given that grey matter volumes have been associated with handgrip strength[12], we repeated the mediation analyses with total grey matter volumes (instead of total intracranial volumes) regressed out from the mediating, dependent and independent variables. All mediation analyses were conducted using the 'lavaan' package on R[15], and the significance of the indirect effects was evaluated using bootstrapped (5000 bootstrap iterations) confidence intervals."

Results (pp. 17-18, lines 393-410)

"**Mediation effect of salience/ventral attention network functional connectivity on the association between handgrip strength and cognitive performance**

For functional connectivity measures that showed significant associations with both handgrip strength and cognitive performance, we additionally ran exploratory mediation analyses to examine if these functional connectivity measures mediated the relationship between handgrip strength and cognitive performance. We found a significant indirect effect of the right anterior insula functional connectivity to the left posterior insula/frontal operculum on the association between handgrip strength and processing speed (proportion of effect size mediated = 27.1%, $p = 0.038$) (Supplementary Table 11), indicating a partial mediation. Indirect effects of the other salience/ventral attention network functional connectivity measures (i.e., segregation, intra-network functional connectivity, and right anterior insula connectivity to the right midcingulate/medial parietal cortex) on the handgrip strength-processing speed association were also observed, however these effects only showed a trend towards significance (proportion mediated = 20.4% – 21.5%, $p = 0.063 – 0.102$) (Supplementary Table 11). For the relationship between handgrip strength and global cognition, no significant indirect effects of the functional connectivity measures were found. These findings remained even after total grey matter volumes (instead of total intracranial volumes) were regressed out from the variables (Supplementary Table 12)."

Discussion (pp. 19, lines 433-436)

"Moreover, exploratory analyses revealed some mediation effects of salience/ventral attention network functional connectivity, particularly the connectivity between the right anterior insula and the left posterior insula/frontal operculum, on the relationship between handgrip strength and processing speed."

Discussion (pp. 21-22, lines 493-503)

"Further, although preliminary at this juncture due to the cross-sectional nature of the study, we demonstrated that functional connectivity between the right anterior insula and the left posterior insula/frontal operculum significantly partially mediated the relationship between handgrip strength and processing speed, even after accounting for variations in total grey matter volume. Given that the mediation effects of other salience/ventral attention network functional connectivity measures also showed a trend towards significance, these findings indicate that inter-individual differences in salience/ventral attention network connectivity may possibly underpin the relationship between handgrip strength and cognition, although future longitudinal studies with larger sample sizes are required to confirm these mediation effects (particularly those with smaller effect sizes)."

Supplementary Table 11: Mediation effects of functional connectivity measures on the association between handgrip strength and cognitive performance (controlling for total intracranial volumes)

Effect	Coeff.	SE	95% CI		z	*p*
			Lower	Upper		
Mediator: S/VA segregation, cognitive measure: global cognition						
a: handgrip str. → FC	$4.90e^{-3}$	$1.55e^{-3}$	$1.85e^{-3}$	$7.99e^{-3}$	3.17	0.0015*
b: FC → cognition	5.97	4.64	-2.63	15.56	1.28	0.1990
c (total): handgrip str. → cognition	0.241	0.075	0.086	0.385	3.20	0.0014*
c' (direct): handgrip str. → cognition	0.212	0.078	0.052	0.360	2.71	0.0068*
ab (indirect): handgrip str. → FC → cognition	0.029	0.027	-0.012	0.094	1.07	0.2828
Proportion mediated	12.1%					
Mediator: S/VA segregation, cognitive measure: processing speed						
a: handgrip str. → FC	$4.90e^{-3}$	$1.55e^{-3}$	$1.87e^{-3}$	$7.89e^{-3}$	3.15	0.0016*
b: FC → cognition	13.22	5.97	2.18	25.54	2.21	0.0268*
c (total): handgrip str. → cognition	0.317	0.096	0.128	0.506	3.31	0.0009*
c' (direct): handgrip str. → cognition	0.252	0.097	0.061	0.444	2.59	0.0096*
ab (indirect): handgrip str. → FC → cognition	0.065	0.039	0.006	0.159	1.65	0.0990
Proportion mediated	20.4%					
Mediator: intra-network S/VA, cognitive measure: global cognition						
a: handgrip str. → FC	$4.29e^{-3}$	$1.51e^{-3}$	$1.28e^{-3}$	$7.17e^{-3}$	2.84	0.0045*
b: FC → cognition	9.09	4.67	-0.02	18.27	1.94	0.0518
c (total): handgrip str. → cognition	0.241	0.075	0.090	0.388	3.20	0.0014*
c' (direct): handgrip str. → cognition	0.202	0.072	0.061	0.342	2.80	0.0052*
ab (indirect): handgrip str. → FC → cognition	0.039	0.025	-0.001	0.096	1.57	0.1167
Proportion mediated	16.2%					
Mediator: intra-network S/VA, cognitive measure: processing speed						
a: handgrip str. → FC	$4.29e^{-3}$	$1.50e^{-3}$	$1.28e^{-3}$	$7.14e^{-3}$	2.85	0.0043*
b: FC → cognition	15.91	6.14	3.93	28.37	2.59	0.0095*
c (total): handgrip str. → cognition	0.317	0.096	0.127	0.501	3.30	0.0010*
c' (direct): handgrip str. → cognition	0.249	0.090	0.070	0.426	2.75	0.0060*
ab (indirect): handgrip str. → FC → cognition	0.068	0.037	0.009	0.150	1.86	0.0629

Proportion mediated	21.5%					
Mediator: R AI – L PI/FO, cognitive measure: global cognition						
a: handgrip str. → FC	$1.54e^{-2}$	$3.59e^{-3}$	$8.25e^{-3}$	$2.22e^{-2}$	4.28	<0.0001*
b: FC → cognition	2.90	1.73	-0.49	6.28	1.68	0.0938
c (total): handgrip str. → cognition	0.241	0.076	0.092	0.386	3.18	0.0015*
c' (direct): handgrip str. → cognition	0.196	0.079	0.041	0.350	2.49	0.0127*
ab (indirect): handgrip str. → FC → cognition	0.045	0.030	-0.007	0.110	1.48	0.1377
Proportion mediated	18.5%					
Mediator: R AI – L PI/FO, cognitive measure: processing speed						
a: handgrip str. → FC	$1.54e^{-2}$	$3.59e^{-3}$	$8.16e^{-3}$	$2.23e^{-2}$	4.28	<0.0001*
b: FC → cognition	5.57	2.16	1.34	9.78	2.58	0.0098*
c (total): handgrip str. → cognition	0.317	0.096	0.132	0.507	3.29	0.0010*
c' (direct): handgrip str. → cognition	0.231	0.096	0.035	0.416	2.40	0.0162*
ab (indirect): handgrip str. → FC → cognition	0.086	0.041	0.017	0.176	2.08	0.0377*
Proportion mediated	27.1%					
Mediator: R AI – R MCC/MPC, cognitive measure: global cognition						
a: handgrip str. → FC	$1.56e^{-2}$	$4.40e^{-3}$	$6.97e^{-3}$	$2.42e^{-2}$	3.55	0.0004*
b: FC → cognition	2.68	1.62	-0.57	5.81	1.65	0.0980
c (total): handgrip str. → cognition	0.241	0.075	0.090	0.385	3.23	0.0012*
c' (direct): handgrip str. → cognition	0.199	0.074	0.050	0.342	2.70	0.0070*
ab (indirect): handgrip str. → FC → cognition	0.042	0.029	-0.009	0.106	1.44	0.1492
Proportion mediated	17.4%					
Mediator: R AI – R MCC/MPC, cognitive measure: processing speed						
a: handgrip str. → FC	$1.56e^{-2}$	$4.51e^{-3}$	$6.54e^{-3}$	$2.43e^{-2}$	3.47	0.0005*
b: FC → cognition	4.18	2.19	-0.05	8.44	1.91	0.0557
c (total): handgrip str. → cognition	0.317	0.096	0.129	0.510	3.30	0.0010*
c' (direct): handgrip str. → cognition	0.251	0.090	0.075	0.423	2.79	0.0053*
ab (indirect): handgrip str. → FC → cognition	0.065	0.040	-0.001	0.157	1.64	0.1017
Proportion mediated	20.7%					

Age, sex, education and total intracranial volumes were regressed from all variables prior to performing the mediation analyses. * indicates statistically significant effects ($p < 0.05$). Abbreviations: Coeff., coefficient; SE, standard error; CI, confidence intervals; S/VA, salience/ventral attention network; handgrip str., handgrip strength; FC, functional connectivity; R, right; L, left; AI, anterior insula; PI, posterior insula; FO, frontal operculum; MCC, midcingulate cortex; MPC, medial parietal cortex.

Supplementary Table 12: Mediation effects of functional connectivity measures on the association between handgrip strength and cognitive performance (controlling for total grey matter volumes)

**Effect**	**Coeff.**	**SE**	**95% CI**		**z**	***p***
			**Lower**	**Upper**		
Mediator: S/VA segregation, cognitive measure: global cognition						
a: handgrip str. → FC	$5.04e^{-3}$	$1.71e^{-3}$	$1.51e^{-3}$	$8.21e^{-3}$	2.95	0.0032*
b: FC → cognition	5.71	4.60	-2.87	15.26	1.24	0.2143
c (total): handgrip str. → cognition	0.218	0.077	0.068	0.365	2.85	0.0044*
c' (direct): handgrip str. → cognition	0.190	0.078	0.034	0.340	2.42	0.0157*
ab (indirect): handgrip str. → FC → cognition	0.029	0.028	-0.013	0.096	1.04	0.2997
Proportion mediated	13.2%					

Mediator: S/VA segregation, cognitive measure: processing speed						
a: handgrip str. → FC	$5.04e^{-3}$	$1.73e^{-3}$	$1.40e^{-3}$	$8.31e^{-3}$	2.92	0.0035*
b: FC → cognition	12.66	5.95	1.79	25.03	2.13	0.0332*
c (total): handgrip str. → cognition	0.301	0.102	0.099	0.498	2.95	0.0031*
c' (direct): handgrip str. → cognition	0.237	0.102	0.031	0.437	2.31	0.0208*
ab (indirect): handgrip str. → FC → cognition	0.064	0.041	0.004	0.162	1.56	0.1183
Proportion mediated	21.2%					
Mediator: intra-network S/VA, cognitive measure: global cognition						
a: handgrip str. → FC	$4.19e^{-3}$	$1.65e^{-3}$	8.14^{-4}	$7.32e^{-3}$	2.54	0.0110*
b: FC → cognition	8.41	4.55	-0.55	17.39	1.85	0.0647
c (total): handgrip str. → cognition	0.218	0.077	0.064	0.365	2.85	0.0044*
c' (direct): handgrip str. → cognition	0.183	0.074	0.033	0.323	2.46	0.0140*
ab (indirect): handgrip str. → FC → cognition	0.035	0.024	-0.004	0.090	1.47	0.1425
Proportion mediated	16.1%					
Mediator: intra-network S/VA, cognitive measure: processing speed						
a: handgrip str. → FC	$4.19e^{-3}$	$1.70e^{-3}$	$8.58e^{-4}$	$7.49e^{-3}$	2.47	0.0136*
b: FC → cognition	14.92	6.04	3.46	26.99	2.47	0.0136*
c (total): handgrip str. → cognition	0.301	0.102	0.106	0.501	2.94	0.0033*
c' (direct): handgrip str. → cognition	0.238	0.098	0.051	0.431	2.44	0.0147*
ab (indirect): handgrip str. → FC → cognition	0.062	0.037	0.005	0.146	1.71	0.0876
Proportion mediated	20.8%					
Mediator: R AI – L PI/FO, cognitive measure: global cognition						
a: handgrip str. → FC	$1.43e^{-2}$	$3.60e^{-3}$	$7.03e^{-3}$	$2.11e^{-2}$	3.98	0.0001*
b: FC → cognition	2.72	1.69	-0.72	5.93	1.61	0.1083
c (total): handgrip str. → cognition	0.218	0.077	0.061	0.364	2.84	0.0045*
c' (direct): handgrip str. → cognition	0.179	0.079	0.021	0.331	2.26	0.0235*
ab (indirect): handgrip str. → FC → cognition	0.039	0.028	-0.010	0.099	1.41	0.1590
Proportion mediated	17.9%					
Mediator: R AI – L PI/FO, cognitive measure: processing speed						
a: handgrip str. → FC	$1.43e^{-2}$	$3.59e^{-3}$	$7.21e^{-3}$	$2.13e^{-2}$	3.99	0.0001*
b: FC → cognition	5.43	2.16	1.21	9.70	2.52	0.0118*
c (total): handgrip str. → cognition	0.301	0.100	0.099	0.491	3.02	0.0025*
c' (direct): handgrip str. → cognition	0.223	0.100	0.020	0.413	2.23	0.0258*
ab (indirect): handgrip str. → FC → cognition	0.078	0.039	0.014	0.166	1.97	0.0485*
Proportion mediated	25.9%					
Mediator: R AI – R MCC/MPC, cognitive measure: global cognition						
a: handgrip str. → FC	$1.74e^{-2}$	$4.55e^{-3}$	$8.48e^{-3}$	$2.65e^{-2}$	3.82	0.0001*
b: FC → cognition	2.77	1.66	-0.42	6.04	1.67	0.0958
c (total): handgrip str. → cognition	0.218	0.076	0.063	0.363	2.86	0.0043*
c' (direct): handgrip str. → cognition	0.170	0.074	0.024	0.314	2.30	0.0215*
ab (indirect): handgrip str. → FC → cognition	0.048	0.033	-0.007	0.120	1.47	0.1426
Proportion mediated	22.0%					
Mediator: R AI – R MCC/MPC, cognitive measure: processing speed						
a: handgrip str. → FC	$1.74e^{-2}$	$4.63e^{-3}$	$7.91e^{-3}$	$2.63e^{-2}$	3.75	0.0002*
b: FC → cognition	4.13	2.22	-0.27	8.53	1.86	0.0629

c (total): handgrip str. → cognition	0.301	0.102	0.095	0.499	2.94	0.0033*
c’ (direct): handgrip str. → cognition	0.229	0.097	0.032	0.414	2.36	0.0183*
ab (indirect): handgrip str. → FC → cognition	0.072	0.044	-0.004	0.168	1.62	0.1057
Proportion mediated	23.8%					

Age, sex, education and total grey matter volumes were regressed from all variables prior to performing the mediation analyses. * indicates statistically significant effects ($p < 0.05$). Abbreviations: Coeff., coefficient; SE, standard error; CI, confidence intervals; S/VA, salience/ventral attention network; handgrip str., handgrip strength; FC, functional connectivity; R, right; L, left; AI, anterior insula; PI, posterior insula; FO, frontal operculum; MCC, midcingulate cortex; MPC, medial parietal cortex.

REFERENCES

1 Caspi, Y. *et al.* Changes in the intracranial volume from early adulthood to the sixth decade of life: A longitudinal study. *Neuroimage*. **220**, 116842, doi:10.1016/j.neuroimage.2020.116842 (2020).

2 Qing, Z. & Gong, G. Size matters to function: Brain volume correlates with intrinsic brain activity across healthy individuals. *Neuroimage*. **139**, 271-278, doi:10.1016/j.neuroimage.2016.06.046 (2016).

3 Du, W., Ding, C., Jiang, J. & Han, Y. Women Exhibit Lower Global Left Frontal Cortex Connectivity Among Cognitively Unimpaired Elderly Individuals: A Pilot Study from SILCODE. *Journal of Alzheimer's disease : JAD*. **83**, 653-663, doi:10.3233/JAD-210376 (2021).

4 Hanggi, J. *et al.* Strength of Structural and Functional Frontostriatal Connectivity Predicts Self-Control in the Healthy Elderly. *Frontiers in Aging Neuroscience*. **8**, 307, doi:10.3389/fnagi.2016.00307 (2016).

5 Lin, C. *et al.* The effect of amyloid deposition on longitudinal resting-state functional connectivity in cognitively normal older adults. *Alzheimers Res Ther*. **12**, 7, doi:10.1186/s13195-019-0573-1 (2020).

6 Zonneveld, H. I. *et al.* Patterns of functional connectivity in an aging population: The Rotterdam Study. *Neuroimage*. **189**, 432-444, doi:10.1016/j.neuroimage.2019.01.041 (2019).

7 Benson, G. *et al.* Functional connectivity in cognitive control networks mitigates the impact of white matter lesions in the elderly. *Alzheimers Res Ther*. **10**, 109, doi:10.1186/s13195-018-0434-3 (2018).

8 Stumme, J. *et al.* Interrelating differences in structural and functional connectivity in the older adult's brain. *Human brain mapping*. **43**, 5543-5561, doi:10.1002/hbm.26030 (2022).

9 Takagi, Y. *et al.* Intergenerational transmission of the patterns of functional and structural brain networks. *iScience*. **24**, 102708, doi:10.1016/j.isci.2021.102708 (2021).

10 Zhao, W. *et al.* Depression mediates the association between insula-frontal functional connectivity and social interaction anxiety. *Human brain mapping*. **43**, 4266-4273, doi:10.1002/hbm.25952 (2022).

11 Chou, M. Y. *et al.* Role of gait speed and grip strength in predicting 10-year cognitive decline among community-dwelling older people. *BMC Geriatr*. **19**, 186, doi:10.1186/s12877-019-1199-7 (2019).

12 Jiang, R. *et al.* Associations between grip strength, brain structure, and mental health in > 40,000 participants from the UK Biobank. *BMC Med*. **20**, 286, doi:10.1186/s12916-022-02490-2 (2022).

13 Sternang, O. *et al.* Grip Strength and Cognitive Abilities: Associations in Old Age. *J Gerontol B Psychol Sci Soc Sci*. **71**, 841-848, doi:10.1093/geronb/gbv017 (2016).

14 Kunutsor, S. K., Isiozor, N. M., Voutilainen, A. & Laukkanen, J. A. Handgrip strength and risk of cognitive outcomes: new prospective study and meta-analysis of 16 observational cohort studies. *Geroscience*. **44**, 2007-2024, doi:10.1007/s11357-022-00514-6 (2022).

15 Rosseel, Y. lavaan: An R Package for Structural Equation Modeling. *Journal of Statistical Software*. **48**, 1 - 36, doi:10.18637/jss.v048.i02 (2012).

REVIEWERS' COMMENTS:

Reviewer #1 (Remarks to the Author):

My concerns have been addressed.

Reviewer #2 (Remarks to the Author):

All my comments have now been addressed.
